# A phase I clinical trial of RNF43 peptide-related immune cell therapy combined with low-dose cyclophosphamide in patients with advanced solid tumors

**DOI:** 10.1371/journal.pone.0187878

**Published:** 2018-01-02

**Authors:** Yasuki Hijikata, Toshihiko Okazaki, Yoshihiro Tanaka, Mutsunori Murahashi, Yuichi Yamada, Kazunari Yamada, Atsushi Takahashi, Hiroyuki Inoue, Junji Kishimoto, Yoichi Nakanishi, Yoshinao Oda, Yusuke Nakamura, Kenzaburo Tani

**Affiliations:** 1 Department of Advanced Cell and Molecular Therapy, Kyushu University Hospital, Fukuoka, Japan; 2 ARO Advanced Medical Center, Kyushu University Hospital, Fukuoka, Japan; 3 Department of Anatomic Pathology, Pathological Sciences, Kyushu University, Fukuoka, Japan; 4 Research Institute of Diseases of Chest, Kyushu University, Fukuoka, Japan; 5 Human genome center, Institute of medical science, University of Tokyo, Tokyo, Japan; 6 Project Division of ALA Advanced Medical Research, Advanced Medical Science of Internal Medicine, The Institute of Medical Science, The University of Tokyo, Tokyo, Japan; Okayama Daigaku, JAPAN

## Abstract

The objective of this study was to investigate the safety and the tolerability of combined cellular immunotherapy with low-dose cyclophosphamide (CPA) in patients with advanced solid tumors. This study targeted a novel tumor-associated antigen, ring finger protein 43 (RNF43). Eligible patients were resistant to standard therapy, HLA-A*24:02- or A*02:01-positive and exhibiting high RNF43 expression in their tumor cells. They were administered 300 mg/m^2^ CPA followed by autologous lymphocytes, preliminarily cultured with autologous RNF43 peptide-pulsed dendritic cells (DCs), RNF43 peptide-pulsed DCs and systemic low dose interleukin-2. The primary endpoint was safety whereas the secondary endpoint was immunological and clinical response to treatment. Ten patients, in total, were enrolled in this trial. Primarily, no adverse events greater than Grade 3 were observed. Six out of 10 patients showed stable disease (SD) on day 49, while 4 other patients showed progressive disease. In addition, one patient with SD exhibited a partial response after the second trial. The frequency of regulatory T cells (Tregs) in patients with SD significantly decreased after CPA administration. The ratio of interferon-γ-producing, tumor-reactive CD8^+^ T cells increased with time in patients with SD. We successfully showed that the combination of immune cell therapy and CPA was safe, might induce tumor-specific immune responses and clinical efficacy, and was accompanied by a decreased ratio of Tregs in patients with RNF43-positive advanced solid tumors.

## Introduction

Cancer is one of the leading causes of death worldwide. There have been significant improvements to date, in standard treatment of cancer patients including surgery, chemotherapy, and radiotherapy. However, the emergence of resistance and subsequent relapse remains a major challenge for the long-term survival of these patients. Immune therapy is expected to play a crucial role to overcome this limitation. Therefore clinical development of new treatment strategies has become a priority for basic and clinical science.

Immune therapies have been developed to actively and specifically stimulate the host immune system using cytokines including high-dose interleukin (IL)-2 [1]; molecular vaccines targeting tumor-associated antigen (TAA) [2]; cellular immunotherapies including dendritic cell (DC) vaccines [3]; and adoptive T-cell therapy such as tumor-infiltrating lymphocyte (TIL), cytotoxic T lymphocyte (CTL), transgenic T-cell receptors, and chimeric antigen receptors [4]. Although effective anti-tumor immune responses have been documented in previous clinical trials, several impediments including the reduced or lost molecular expressions of tumor antigen and class I, and the production of immunosuppressive cytokines, immunosuppressive cells including regulatory T cells (Tregs) and immune checkpoint molecules have been found to inhibit anti-tumor immunity [3,5,6]. DCs, the most potent antigen-presenting cells, play a central role in the induction of antigen-specific CTLs. DCs pulsed with TAAs are considered to induce CTLs which target malignant cells expressing the respective TAA protein. The United States Food and Drug Administration (US-FDA) has approved Sipuleucel-T, containing DCs pulsed with TAAs and granulocyte-macrophage colony-stimulating factor of chimeric origin. However, this treatment showed no effect on time-to-disease- progression in clinical trials and is too expensive to justify its use by the National Health Service in the United Kingdom [7,8]. A phase III trial of autologous peptide-pulsed DCs as first-line treatment for patients with metastatic melanoma was not found to be more effective than standard dacarbazine chemotherapy [9]. Clinically effective DC monotherapy for solid tumors remains a distant goal. However, there is emerging evidence that the advantage of DC-based immunotherapy could be more effectively utilized in combination with other anticancer therapies by suppressing immune tolerance and enhancing antitumor T cells activity [3]. One potential candidate is cyclophosphamide (CPA), which was demonstrated to deplete Tregs in preclinical, adoptive T-cell and vaccine models [10], promising an enhanced antitumor immune response via other mechanisms [11]. Additionally, immune checkpoint blockades are proof-of-principle that immunotherapy can overcome immune tolerance [6], but these new modalities are expensive because of the requirement for continual administration. Another candidate therapy is the combined administration of adoptive T cells and DCs [12]. IL-2 has also been shown to significantly prolong T cell survival in vivo [13].

We previously conducted a phase I clinical study using autologous tumor lysate-pulsed, monocyte-derived, mature DC vaccinations combined with low-dose IL-2 in patients with stage IV malignant melanoma, which showed that the treatment was safe [14]. Additionally, we recently reported the efficacy of multiplex vaccination with HLA-A*24:02-restricted TAAs, preceded by the administration of low-dose CPA to eliminate Tregs [15]. These previous studies verified the feasibility of using CPA, DCs, and low-dose IL-2 in patients with advanced cancer.

Ring finger protein 43 (RNF43), overexpressed in colon cancer and involved in tumor growth, was first identified using cDNA microarray profiling [16,17]. RNF43 is up-regulated in > 80% colorectal cancer tissues as compared to the corresponding noncancerous mucosa. Previous studies report that RNF43 is expressed at higher levels in tumors of various histological origins as compared to normal tissue, except for the testes. Therefore, RNF43 is considered a valid target for anti-tumor, immunotherapeutic treatment [18,19] and the combination with DC vaccination is expected to synergistically increase the antitumor immunity targeting RNF43.

Here we report a phase I clinical trial of combined immune cell therapy consisting of autologous RNF43 peptide-pulsed DCs and DC-activated killer lymphocytes (DAKs) with CPA and systemic low-dose IL-2 in patients with advanced solid tumors. This study was designated as a dose-escalation study of DAKs, targeting the novel HLA-A*02:01- or HLA-A*24:02-restricted TAAs of RNF43 [18]. The purpose of this trial was to investigate the safety and tolerability of the proposed method as primary endpoint in addition to efficacy, including overall survival and immune response, as the secondary endpoints.

## Materials and methods

### Patients

The eligibility criteria were as follows:

1. Patients with advanced solid tumors, previously treated with available standard therapies;
2. Eastern Cooperative Oncology Group (ECOG) performance status of 0 to 1;
3. HLA-A*24:02- or A*02:01-positive status;
4. Polymerase chain reaction (PCR)-confirmed RNF43-antigen expression of tumor cells;
5. Age between 20 and 70 years;
6. No prior therapy within 4 weeks of enrollment;
7. Adequate hematology (Hemoglobin, > 8 g/dL; WBC, > 3000 cells/μl; Platelets, > 10^5^/μl), renal (creatinine, < 1.5 mg/dL) and hepatic function (total bilirubin < 2.0 mg/dL, aspartate aminotransferase < 99 U/L, alanine aminotransferase < 90 U/L);
8. Presence of measurable tumor, to allow assessment of clinical response;
9. Life expectancy of at least 3 months;
10. Negative for hepatitis B antigen, anti-hepatitis C antibody, anti-HIV antibody, anti-HTLV-1 antibody, and syphilis serodiagnosis;
11. Written informed consent obtained at the time of enrollment.

The following exclusion criteria were applied:

1. Patients with severe pre-existing diseases;
2. Presence of autoimmune disease, active infectious disease, cardiovascular disorders, respiratory disorders, renal dysfunction, immunodeficiency, and hematological disorders;
3. Pregnant, lactating, or possibly pregnant women, or those willing to be pregnant, or willing male partner;
4. Presence of brain metastases;
5. Patients who required systemic administration of steroid or immunosuppressive agents;
6. Patients who were inappropriate for study entry, as judged by the attending physician.

### Study design and treatment

**Fig 1:**
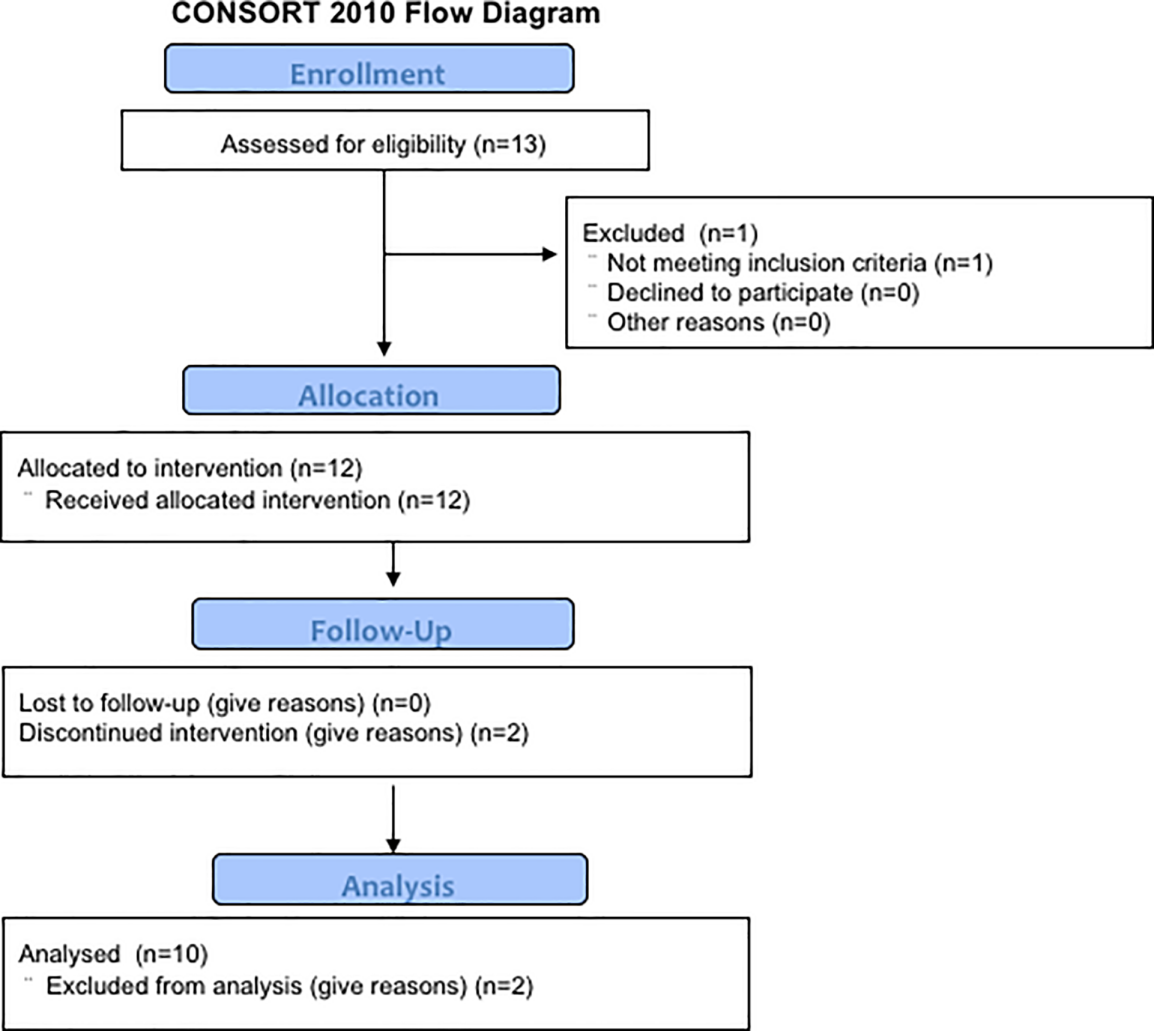
Study CONSORT flow diagram. Two of the twelve enrolled patients did not complete the trial and therefore were not evaluable by CT imaging and immunological analyses. One of the two discontinued because the one chose other treatments, and the other died from rapid disease progression.

**Fig 2:**
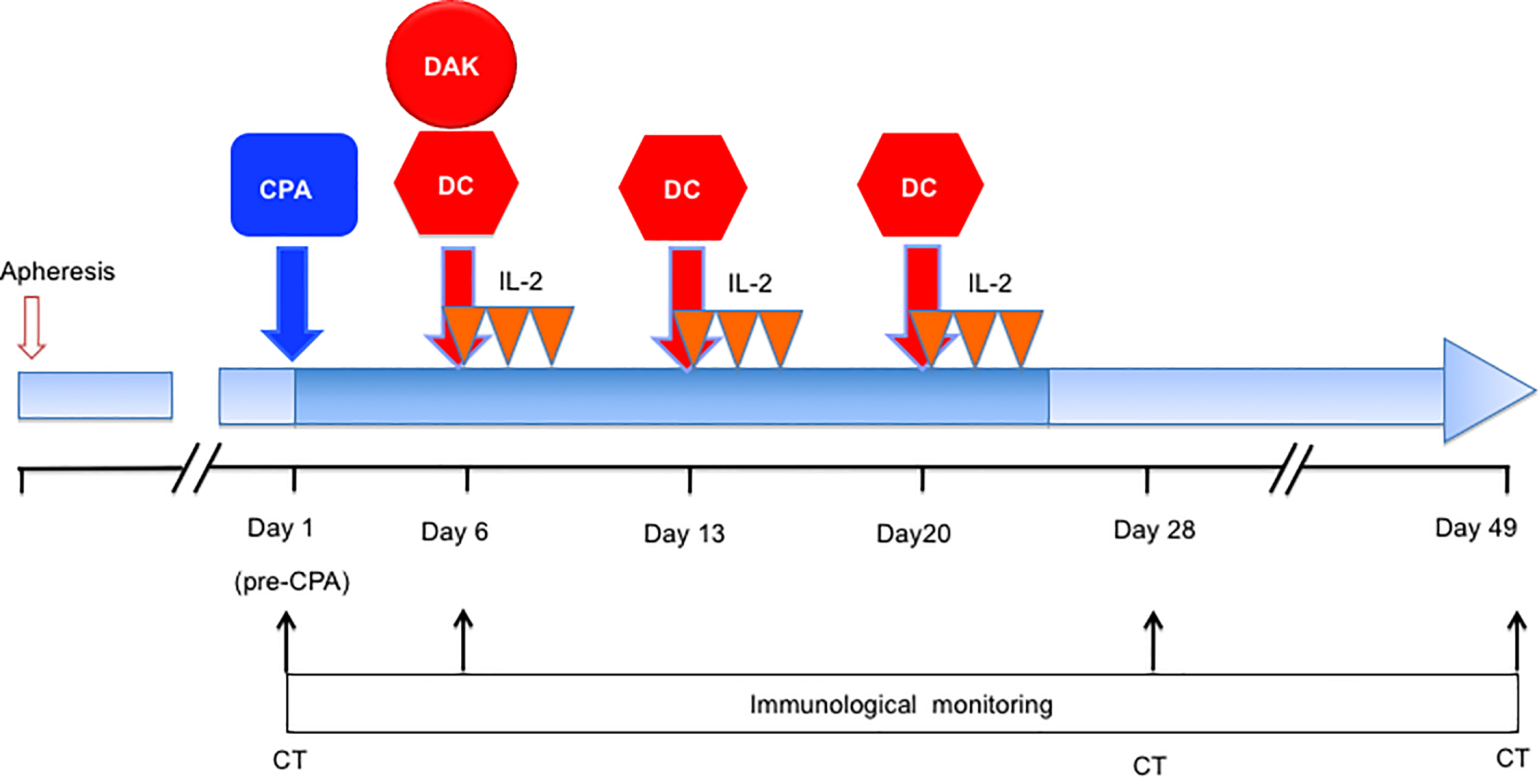
Clinical protocol of RNF43 peptide-related immune cell therapy combined with low-dose cyclophosphamide (CPA). CPA was administered on day 1. DC-activated killer lymphocytes (DAKs) were administered intravenously on Day 6. DCs were injected subcutaneously on days 6, 13, and 20. IL-2 was administered sequentially for 3 days after each DC vaccination. Immunological monitoring was performed on days 1, 6, 28, and 49. Computed tomography (CT) was performed on days 1, 28, and 49.

This was a nonrandomized, open-label, phase I clinical trial, with dose escalation of the DAKs in patients with advanced solid tumors, to assess safety of the treatment. Subjects were enrolled into 2 cohorts (levels 1 and 2) in this dose-escalation study, wherein 5 patients each in levels 1 and 2 respectively received 5 × 10^7^ and 2 × 10^8^ DAKs (see Fig 1 for CONSORT diagram). The protocol for this trial and supporting TREND checklist are available as supporting information; see S1–S3 Files. Immunological responses, clinical responses, and overall survival were examined as secondary endpoints. Leukapheresis was performed 23 days prior to the administration of CPA. Low-dose CPA at 300 mg/m^2^ was administered to patients on Day 1. DAKs were administered on Day 6 intravenously and 1 × 10^7^ DCs were injected subcutaneously once a week, on Days 6, 13, and 20. Low-dose IL-2, at 3.5 × 10^5^ IU, was injected subcutaneously on Days 6, 7, and 8, sequentially after each DC vaccination (Fig 2). All of these procedures were done by physicians in charge. The evaluation was assessed on Days 1, 28, and 49 (Fig 2). Additional courses of the treatment were allowed after receiving approval from the case management committee in case the patient showed no severe adverse events and a clinical response higher than stable disease (SD). The study protocol was approved by the Institutional Review Board (IRB) of the Kyushu University Hospital, and written informed consent was obtained from all patients at the time of enrollment. The first version of this clinical study protocol was approved by IRB of Kyushu University Hospital on 18 January 2006 (approval number is 299), when the registration to the University Hospital Medical Information Network Clinical Trials Registry (UMIN-CTR) was not demanded by the Administration of Health, Labor and Welfare that excluded the obligation of registration about the clinical trials approved before 1 April 2009 as well as the IRB of Kyushu University Hospital, and carried out in the Kyushu University Hospital between March, 2009 and February, 2015. As we optionally registered to the UMIN-CTR from 25 July 2010, 8 of 10 cases were registered. The name of the registry is a phase 1 study of adoptive immunotherapy using autologous RNF43 peptide pulse dendritic cells and RNF43 peptide specifically activated lymphocytes in patient with advanced solid tumors. The registry number is UMIN000003945. The patients were recruited by Japanese clinicians through or not through UMIN-CTR.

### Blood sample and cell lines

Peripheral blood mononuclear cells (PBMCs) and serum were sampled and analyzed in the Kyushu University Hospital on days 1 (Pre-CPA), 6, 28, and 49. The samples were cryopreserved in liquid nitrogen or at -150°C until use. The tumor cell line HCT-15, which expresses high levels of *RNF43* as verified by reverse transcription-PCR (RT-PCR), was purchased from the American Type Culture Collection (ATCC CCL-225^TM^; Manassas, USA). HCT-15 cells were cultured at 37°C with 5% CO_2_, in RPMI-1640 medium (Nacalai Tesque; Tokyo, Japan) containing 10% fetal bovine serum (Sigma-Aldrich, St. Louis, USA), 50 U/ml penicillin (Nacalai Tesque; Tokyo, Japan) and 50 μg/ml streptmycine (Nacalai Tesque; Tokyo, Japan), and these cells were used as positive control of RNF43 expressing cells in the following studies.

### Quantitative real-time PCR analysis for *RNF43* mRNA from patient tumors

*RNF43* gene expression was analyzed by real-time, quantitative RT-PCR of biopsies or surgical specimens of tumors collected from all patients after obtaining the written informed consents. Total RNA was extracted from tumor tissue using RNeasy Mini Kit (Qiagen GmbH, Hilden, Germany). The concentrations of extracted RNA and ratio of absorbance at 260 to that at 280 nm (*A*_260_/*A*_280_ ratio) were measured with a NanoDrop ND-1000 spectrophotometer (Thermo Fisher Scientific, MA, USA). Single-stranded cDNA was synthesized from 1 μg total RNA using QuantiTect Reverse Transcription Kit (Qiagen GmbH, Hilden, Germany). *RNF43* gene expression analysis by real-time RT-PCR was assessed using QuantiTect SYBR Green PCR Kits (Qiagen GmbH, Hilden, Germany) and LightCycler 1.5 (Roche Diagnostics, Mannheim, Germany). In addition, glyceraldehyde-3-phosphate dehydrogenase (*GAPDH*) gene was used as internal control. The primers used for *RNF43* (Hs_FLJ20315_1_SG QuantiTect Primer Assay [Cat. No. QT00075915]) and *GAPDH* (Hs_GAPDH_2_SG QuantiTect Primer Assay [Cat. No. QT01192646]) were purchased from Qiagen GmbH (Hilden, Germany). Gene expression level of *RNF43* was calculated by *RNF43/GAPDH* and relative expression ratio (versus HCT-15 cells) for tumor.

### Preparation of DCs and DAKs

Leukapheresis was performed using a cell separator (COBE® Spectra Apheresis System) (TERUMOBCT, Lakewood, CO). PBMCs were isolated from resulting peripheral blood with Ficoll-Hypaque plus (GE Healthcare, Buckinghamshire, England). PBMCs were suspended in X-VIVO™ 15 medium (Lonza, MD) and allowed to adhere on to plastic plates for 2 h. Non-adherent cells were collected and frozen at -80°C in KM Banker II (Cosmo Bio, Tokyo, Japan) for later use as effector cells. The adherent cells were subsequently cultured for 5 days with 1,000 U/ml granulocyte macrophage colony-stimulating factor (GM-CSF; Bayer HealthCare, Leverkusen, Germany) and 1,000 U/ml CellGro® Good Manufacturing Practice (GMP)-grade IL-4 (CellGenix, Freiburg, Germany) in GMP serum-free DC medium (CellGenix, Freiburg, Germany). On Day 6, 50% of the DC medium was removed and fresh DC medium containing 50 ng/μl CellGro® GMP tumor necrosis factor-α (TNF-α; CellGenix) and 25 μg/ml keyhole limpet hemocyanin (Merck Millipore Darmstadt, Germany) was added. On day 7, 0.1 KE/ml OK432 (Chugai Pharmaceutical, Osaka, Japan) was added to the culture medium to induce terminal maturation of the DCs.

On day 8, harvested cells were pulsed with 20 μg/ml of HLA-A*24:02-restricted RNF43 peptide (RNF43-A24-9-721, sequence: NSQPVWLCL) for HLA-A*24:02-positive patients and 20 μg/ml of HLA-A*02:01-restricted RNF43 peptide (RNF43-A02-10-11, sequence: ALWPWLLMAT) for HLA-A*02:01-positive patients, for 2 h. The RNF43 peptides were synthesized by PolyPeptide Laboratories (San Diego, CA, USA). Mature, RNF43-pulsed DCs were obtained from each patient and prepared as described above, washed, and frozen until use for in vitro stimulation of non-adherent cells or injection into patients. A part of the DCs were analyzed for phenotype using flow cytometry. An adequate quantity of DCs were prepared so that >4.5 × 10^7^ cells were available for each course of therapy. The remaining portion of the 5 × 10^6^ RNF43-pulsed DCs were used to stimulate 1×10^8^ non-adherent cells, which were then co-cultured with cytokines such as 100 IU/ml IL-2 (Novartis, Basel, Switzerland), 25 ng/ml IL-7 (R&D Systems, Minneapolis, USA), 400 pg/ml IL-12 (R&D Systems, Minneapolis, USA), and 25 ng/ml IL-15 (Thermo Fisher Scientific, MA, USA) every 2 to 3 days in X-VIVO™ 10 (Lonza, MD) to induce long-term survival of DAKs [20,21]. After three cycles of weekly stimulation, more than 2.0 × 10^8^ DAKs were harvested and analyzed phenotypically by flow cytometry. All cells were prepared as per GMP grade at the Kyushu University Molecular and Cell Processing Center (KU-MCPC) and endotoxin levels as well as bio-burden of these products were confirmed as within acceptable levels of GMP-grade immune cell therapy.

### Flow cytometry

Cells including patients' PBMCs, manufactured DCs, and DAKs were analyzed using the following mouse anti-human monoclonal antibodies: fluorescein 5-isothiocyanate (FITC)-labeled anti-CD8 (Cat. No. 347313), anti-CD62L (Cat. No. 555543), and lineage cocktail 1 (CD3, CD14, CD16, CD19, CD20, and CD56) (Cat. No. 340546); phycoerythrin (PE)-labeled anti-CD40 (Cat. No. 555589), anti-CD80 (Cat. No. 557227), anti-CD83 (Cat. No. 556855), anti-CD86 (Cat. No. 555658), anti-HLA-A, B and C (Cat. No. 555553), anti-CD4 (Cat. No. 555347), anti-CD25 (Cat. No. 555432), anti-interferon-γ (IFN-γ) (Cat. No. 340452), anti-IL-2 (Cat. No. 340450), and anti-TNF-α (Cat. No. 340512); PE-Cy7-labeled anti-HLA-DR (Cat. No. 335795) and anti-CD45RA (Cat. No. 337167); allophycocyanin (APC)-labeled anti-CD11c (Cat. No. 559877), anti-CD62L (Cat. No. 559772), and anti-CD8 (Cat. No. 340584); APC-H7-labeled anti-CD3 (Cat. No. 641397) and anti-CD4 (Cat. No. 560158); PerCP-Cy5.5-labeled anti-CD4 (Cat. No. 341654); Alexa Fluor 488-labeled anti-3 (Cat. No. 560047); and Alexa Fluor 647-labeled anti-IL-17A (Cat. No. 560491). All antibodies and the appropriate isotype controls were obtained from BD Biosciences (San Jose, CA, USA). The measurement and analysis of flow cytometry was performed using BD FACS Canto™ II and BD FACS Diva software v6.1.2 (BD Biosciences, San Jose, CA, USA) according to the manufacturer’s protocol.

### Detection of Tregs and Th17 cells in patient PBMCs

PBMCs (2.5 × 10^5^ cells) collected from each patient at the four time points (days 1, 6, 28, and 49) were incubated with anti-CD4 (APC-H7) and anti-CD25 (PE) for 20 min at room temperature. Samples were permeabilized, and fixed with human Foxp3 buffer set (BD Biosciences, San Jose, CA, USA). The cells were then stained with anti-Foxp3 and anti-IL-17A for 40 min at room temperature. Activated Tregs were defined as CD4^+^ CD25^high^ Foxp3^+^ (S5 Fig).

### Intracellular cytokine staining (ICS) assay and PBMC phenotype analysis

Direct ICS assay was performed as previously described [22–24] using thawed PBMCs obtained on days 1 (pre-CPA), 28, and 49. For each sample, 5.0 × 10^5^ PBMCs were stimulated with the RNF43 peptide (10 μg/mL). Cultures were incubated for 2 h at 37°C, followed by a 4-h incubation in phosphate-buffer saline (PBS; Thermo Fisher Scientific, MA, USA) containing Brefeldin A (BFA; 10 μg/ml, Sigma-Aldrich, St. Louis, USA) and GolgiStop (BD Biosciences, San Jose, CA, USA. 1:1500 dilution). The cells were then stained with anti-CD62L (FITC), anti-CD4 (PerCP-Cy5.5), anti-CD45RA, anti-CD8 (APC), and anti-CD3 for 20 min at room temperature. Cells were then permeabilized and fixed with human Foxp3 buffer set (BD Biosciences), followed by ICS using anti-human mouse antibodies for anti-IFN-γ, anti-IL-2, and anti-TNF-α for 30 min at room temperature. Cells were acquired through a standard lymphocyte light-scatter gate and gated CD3^+^CD8^+^ T cells were analyzed for cytokines using flow cytometry (BD FACS Canto™ II, BD Biosciences). The background levels of these expressed cytokines, measured by stimulation with negative-control peptides of HLA-A*24:02 HIV env gp160 peptide or the HLA-A*02:01 HIV gag peptide (Medical & Biological Laboratories, Nagoya, Japan), were subtracted from those levels induced by the corresponding RNF43 peptide (RNF43-A24-9-721 or RNF43-A02-10-11, (PolyPeptide Laboratories, Torrance, CA, USA)).

### Measurement of serum cytokine levels

Serum levels of 8 cytokines, namely IFN-γ, TNF-α, IL-1β, IL-2, IL-4, IL-6, IL-10, and IL-17A (BD Biosciences, San Jose, CA, USA), from patient peripheral blood, obtained at days 1 (pre-CPA), 28, and 49 were quantified by Cytometric Bead Array (CBA) Flex system (BD Biosciences, San Jose, CA, USA).

### Immunohistochemistry

To detect RNF43 protein and infiltrating lymphocytes in tumor tissues, formalin-fixed, paraffin-embedded tissue was cut to obtain 3-μm sections. Antigen retrieval was carried out by boiling the slides with 10 mM sodium citrate (pH 6.0) or Target Retrieval Solution (DAKO, Carpinteria, CA). Anti-RNF43 (1:100; Sigma-Aldrich, St. Louis, MO, USA), anti-Foxp3 (1:100; Abcam, Cambridge, UK), anti-HLA class 1 (1:1000; Hokudo, Sapporo, Japan), anti-CD3 (Nichirei, Hiroshima, Japan), anti-CD4 (1:100; Carpinteria, CA, USA) and anti-CD8 (1:100; MBL Aichi, Japan) monoclonal antibodies were used. The immune complex was detected using the DAKO EnVision Detection System. The percentage of RNF43-positive carcinoma cells were evaluated in the biopsied specimens at the most representative areas prior to this treatment. We assessed the immunoreactivity of anti-RNF43 antibody with labeling index (LI). To determine the LI, the positively stained nuclei in every 100 tumor cells were counted in three different areas of each tumor. RNF43 LI > 10% was defined as positive. The stained lymphocytes in the area of the tumor and representative invasive margin are counted per high-power fields [25].

### DNA sequencing of *RNF43* transcripts from tumors

Total RNA was extracted from patient tumor tissues using RNeasy Mini Kit (Qiagen GmbH, Hilden, Germany) and cDNA was synthesized using QuantiTect Reverse Transcription Kit (Qiagen GmbH), according to the manufacturer’s protocols. PCR samples were amplified using MyCycler Thermal Cycler (BioRad Laboratories, California, USA) and KOD FX Neo (Toyobo, Osaka, Japan). We performed *RNF43* transcript analysis by RT-PCR of tumor mRNA focusing on two recurrent hotspot mutations of G659fs and R117fs, accounting for 41.7% to 48.0% and 8.3% to 12.0% of *RNF43* mutations identified in colon cancer and endometrial cancer [26]. Primer sequences used were as follows: mutation 1 seq S (5′-AGCGGTGGAGTCTGAAAGAT-3′), mutation 1 seq AS (5′-TCAGCTCAATCCTCACATGG-3′), mutation 2 seq S (5′ -CAGAGCCACCTTCTCCTGAT-3′) and mutation 2 seq AS (5′-CCACACTGGCTGTGAATTTG-3′). DNA sequencing and mutation analysis of *RNF43* were performed by Sanger sequencing on an ABI3130xl Genetic Analyzer (Applied Biosystems, CA, USA) using BigDye® Terminator v3.1 Cycle Sequencing Kit (Thermo Fisher Scientific, MA, USA) [26].

### Statistical analysis

All of the measurements were performed by specialists at the Kyushu University hospital who were unaware of the objective of this trial (S4 File). Based on the RECIST criteria, the patients were categorized into 2 groups showing different therapeutic effects at the end of treatment (Day 49), namely the treatment-effective group (complete response [CR], PR, and SD) and the treatment-ineffective group (PD). Changes in biomarker levels from the baseline were assessed with paired *t*-tests, unpaired *t*-tests for clinical response and logistic-regression analysis was performed to evaluate corrective factors for clinical responses. Because these statistical analyses were conducted for explorative purposes, the significance levels were not adjusted for multiple comparisons. All statistical analyses were performed using JMP 11 software (SAS Institute Inc., Cary, NC) by specialists.

## Results

### Patient characteristics

To meet the goal of 10 patients completing this trial, 12 patients were recruited from January, 2006 to February, 2015. Ten patients completed the 10-week run-in period of the trial, comprising of 6 males and 4 females. The ages of patients were between 38 and 68 years old, with a median age of 57 years. Of the 10 enrolled patients, 7 had colorectal cancer and the other 3 had either small cell lung cancer, esophageal cancer, or cervical cancer. A total of 6 out of 10 patients achieved SD and 5 out of 10 patients lived more than 300 days with or without chemotherapy. Patient characteristics are presented in Table 1. Two of the twelve enrolled patients did not complete the trial and therefore were not evaluable by CT imaging and immunological analyses. One of the two discontinued because the one chose other treatments, and the other died from rapid disease progression.

**Table 1:** Patient characteristics and clinical responses.

Patient No.	Age/ Sex	DAKs dose level	Diagnosis	HLA-typing	RECIST Day28	RECIST Day49	OS(days)	Post-treatment
KU-1	62/M	1	SCLC	A*24:02	PD	PD	65	Chemotherapy
KU-2	68/M	1	CRC	A*02:01	SD	PD	329	Chemotherapy
KU-3	38/M	1	CRC	A*24:02	SD	SD	675	2nd trial, Chemo
KU-4	48/F	1	CC	A*24:02	SD	SD	141	2nd trial
KU-5	64/M	1	EC	A*24:02	SD	SD	266	2nd trial
KU-6	40/M	2	CRC	A*24:02	SD	PD	387	Chemotherapy
KU-7	58/F	2	CRC	A*24:02	SD	SD	265	-
KU-8	67/M	2	CRC	A*02:01	SD	SD	788	2nd trial, Chemo
KU-9	57/F	2	CRC	A*24:02	SD	PD	90	-
KU-10	49/F	2	CRC	A*24:02	SD	SD	Alive (1250)	3 additional trials, Chemo +Hyperthermia

CRC, colorectal cancer; SCLC, small cell lung carcinoma; EC, esophageal cancer; CC, uterine cervical cancer; SD, stable disease; PD, progressive disease; RECIST, Response Evaluation Criteria in Solid Tumors; OS, overall survival; DAKs, DC-activated killer lymphocytes; Chemo, chemotherapy

### The immune phenotypes of DAKs and DCs

Cell processing for both RNF43-pulsed DCs and DAKs was performed safely and successfully at the KU-MCPC. The percentage expression levels of cell surface markers on RNF43-pulsed DCs and DAKs were analyzed (S1 and S2 Figs). The viability of all samples was > 90%. The final yield of DAKs was between 1.49 ×10^8^ cells and 5.72 × 10^8^ cells (average of 3.31 × 10^8^ cells). The final yield of mature RNF43-pulsed DCs was between 7.12 × 10^7^ cells and 2.08 × 10^8^ cells (average of 1.15 × 10^8^ cells). Representative results are shown in Table 2A and 2B. These results showed that the central memory CD8^+^ T cells (CD45RA^-^CD62L^+^) in DAKs tended to be expanded in patients with SD more so than in patients with PD, whereas the effector memory CD8^+^ T cells (CD45RA^-^CD62L^-^) in DAKs tended to be expanded in patients with PD more so than in patients with SD (Tables 1 and 2A). The DCs were determined by expression of CD11c, CD40, CD80, CD83, CD86, HLA-ABC, and HLA-DR (S2 Fig). The DCs showed almost mature immune phenotypes in all patients (Table 2B).

**Table 2:** Immunophenotypes of administered DAKs and RNF43 peptide-pulsed DC.

**a**						DAKs			
Clinical Response	Patient	CD3+CD8+(%)				CD3+CD4+(%)			
	No.	Naïve	CM	EM	TE	Naïve	CM	EM	TE
	Mean	1.39	5.92	34.95	3.5	1.65	12.09	29.6	2.23
	KU-1	0.1	0.47	17.9	1.2	0.16	1.33	74.4	2.5
PD	KU-2	0.36	1.21	57.4	1.76	0.58	1.61	24.6	3.58
(day 49)	KU-6	2.99	10.8	23.9	1.05	3.54	35.4	8.88	0.78
	KU-9	2.11	11.2	40.6	10	2.32	10	10.5	2.04
	Mean	2.75	10.78	20.78	2.15	2.37	24.87	27.08	1.32
	KU-3	0.14	0.18	29.7	5.05	0.24	2.65	55.7	0.35
SD	KU-4	9.55	11.2	26.3	0.91	5.67	9.17	4.2	0.86
(day 49)	KU-5	0.2	9.11	19.2	0.64	0.07	11.7	56.3	0.62
	KU-7	2.28	10.9	12.1	0.33	2.9	58.1	17	0.39
	KU-8	1.64	19.5	24.3	2.6	2.71	30.2	17.2	1.18
	KU-10	2.66	13.8	13.1	3.39	2.6	37.4	12.1	4.53
**b**							RNF43 peptide-pulsed DC		
Clinical Response	Patient	CD11c+	CD11c+						
	No.	Cells (%)	CD40+ (%)	CD80+ (%)	CD83+ (%)	CD86+ (%)	HLA-ABC+ (%)	HLA-DR+ (%)	
	Mean	98.98	98.98	93.15	74.55	99.53	84.55	99.88	
	KU-1	99.1	100	73.2	4.5	98.5	52.7	99.9	
PD	KU-2	97.1	99.6	99.4	98.1	99.6	91.3	99.6	
(day 49)	KU-6	99.9	99.6	100	98.4	100	99.1	100	
	KU-9	99.8	96.7	100	97.2	100	95.1	100	
	Mean	99.38	96.15	99.72	84.78	99.97	85.33	99.53	
	KU-3	97.3	99.9	98.3	30.4	99.9	52.5	99.7	
	KU-4	99.9	87.6	100	91.5	99.9	83.5	100	
SD	KU-5	100	97	100	99.5	100	92.6	100	
(day 49)	KU-7	99.5	98.7	100	91.8	100	92.5	100	
	KU-8	99.9	94.3	100	95.7	100	91.1	97.5	
	KU-10	99.7	99.4	100	99.8	100	99.8	100	

Naïve, (CD45RA+CD62L+); CM, central memory (CD45RA-CD62L+); EM, effector memory (CD45RA-CD62L-); TE, terminal effector (CD45RA+CD62L-);

### Feasibility and safety

Our results demonstrated the feasibility of producing and administering at least a total of 3× 10^7^ DCs and 5 × 10^7^ to 2 × 10^8^ DAKs in adequate quantity for a 2-dose escalation study, from patients with advanced solid tumors, using clinically relevant and safe cell manufacturing procedures at KU-MCPC. Adverse events were evaluated according to the Common Terminology Criteria for Adverse Events v4.0 after the administration of CPA. The results are summarized for all patients in Table 3. No severe adverse events more than Grade 3 were observed. All patients showed Grade 1 or 2 dermal reaction at the injection site of DC or IL-2. No treatment-related hematologic, hepatic, renal, or neurological toxicities were observed.

**Table 3:** Therapy-associated toxicities (CTCTAE version 4.0).

Toxicity	DAKs dose level 1 (n = 5)			DAKs dose level 2 (n = 5)			Total
	Grade1	Grade2	Grade3	Grade1	Grade2	Grade3	
Injection site reaction	3	2	0	4	1	0	10
Fatigue	0	0	0	2	0	0	2
Anorexia	1	0	0	1	0	0	2
Fever	1	0	0	1	0	0	2
Headach	0	0	0	0	0	0	1
Hot flashes	1	0	0	0	0	0	1
Concentration impairment	0	0	0	1	0	0	1

### Clinical evaluation

**Fig 3:**
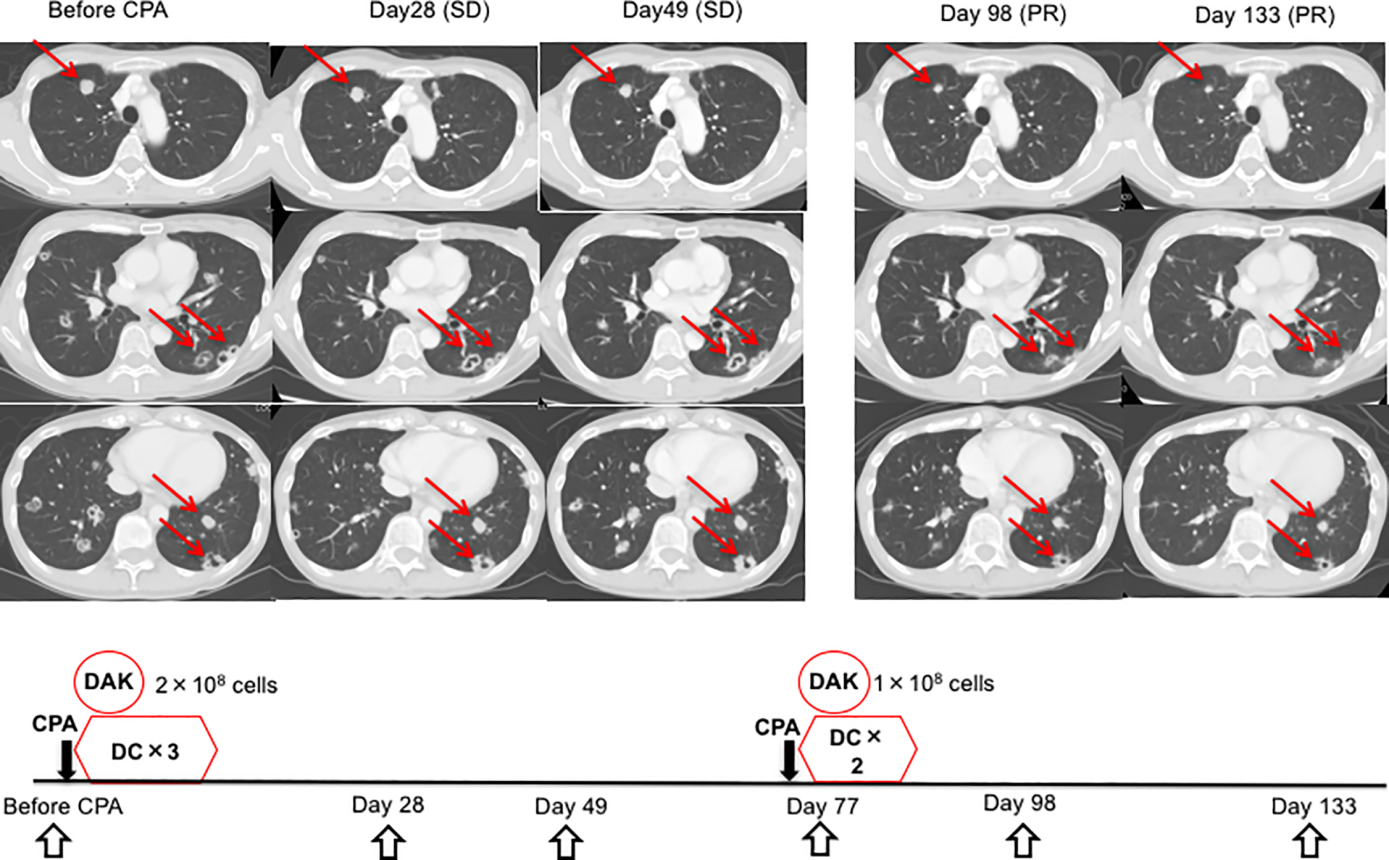
The regression of multiple metastases of colon cancer in KU-10 after the administration of DCs and DAKs. Computerized tomography images at day 93 and day 133 showed partial response (PR) of multiple lung metastasis of colon cancer. Arrows point at responding lesions. PR lasted more than 1250 days.

SD was observed in 6 out of the 10 patients on day 49 (Table 1). Of these, 2 patients (KU-4 and KU-5) experienced a decrease of serum tumor marker levels without any change of tumor size during the observation period (S3 and S4 Figs). In addition, 1 patient (KU-10) achieved a partial response against lung metastasis after the second course of DCs and DAKs, which lasted more than 1250 days (Fig 3 and Table 1). This patient was evaluated as showing SD on day 49, followed by an increase in a part of the tumor burden for a short period, but subsequently evaluated as having achieved an objective response (Fig 3). Four other patients, however, showed PD at day 49 (Table 1).

### Reduced frequency of peripheral blood Tregs by CPA

**Fig 4:**
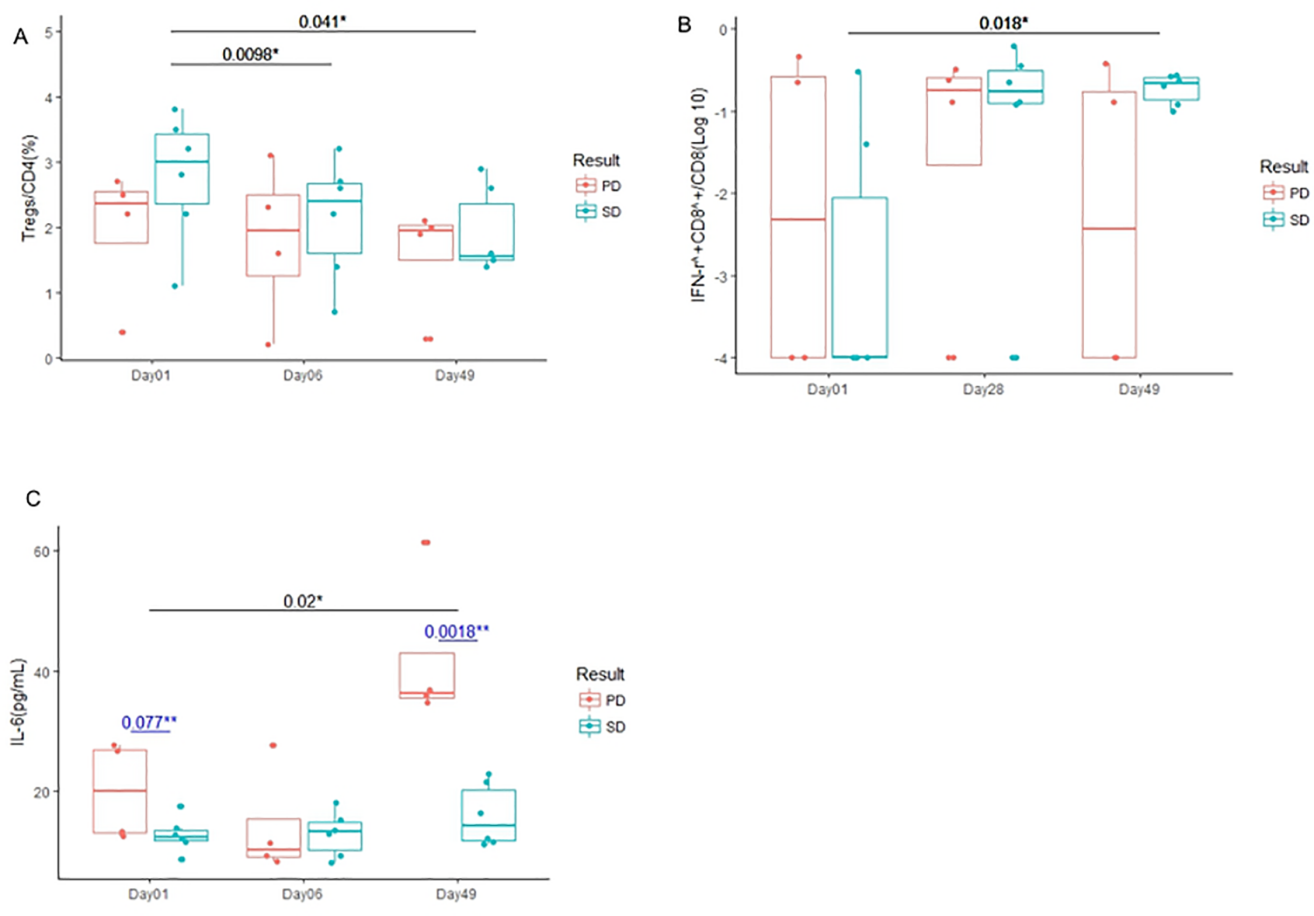
Comparison of patients' immune status between SD and PD. Box plots represent median and range of changes in immune status in patients with SD (blue) and PD (red) during this trial period. *, paired t test; **, unpaired t test. (**A**) The frequency of peripheral blood Tregs in patients with SD decreased significantly after systemic CPA administration and this treatment (p = 0.0098 on day 6 and p = 0.041 on day 49). (**B**) Intracellular cytokine staining demonstrated that IFN-γ produced by tumor-reactive CD8^+^ T cells in patients with SD after this treatment increased significantly compared with pre-CPA (p = 0.018). (**C**) Serum IL-6 levels in patients with PD increased significantly after this treatment (p = 0.020). IL-6 levels in patients with PD were higher than those with SD at the pre-CPA time point (p = 0.077; 95% CI, -0.993 to 15.548).

The frequency of peripheral blood Tregs in all patients significantly decreased at 6 days (p = 0.013; 95% confidence interval [CI], -0.1197 to -0.7603) and 49 days (p = 0.009; 95% CI, -0.2073 to -1.1127) after systemic CPA administration (S5 Fig) and decreased further in patients with SD as compared to those with PD (p = 0.0098 on Day 6; 95% CI, 0.231 to 1.035 and p = 0.041 on Day 49; 95% CI, 0.05421 to 1.6458) (Fig 4A). There was no statistically significant change in the frequency of Th17 cell between before and after treatment, SD and PD.

### Cytokine production by T cells after stimulation with the RNF43-peptide

The ICS assay has been reported as a potentially sensitive and functional assay that can generate quantitative phenotypic and polyfunctional data about responding T cell populations, making the assay valuable for functional profiling of low-level vaccine-induced T cell responses [23]. We performed ICS assays to detect tumor-reactive CD4^+^ or CD8^+^ T cells. It was found that the ratio of IFN-γ produced by tumor-reactive CD8^+^ T cells in patients with SD was significantly higher than those with PD between pre-CPA (day 1) and day 49 (p = 0.018; 95% CI, -3.922 to -0.580) (Fig 4B).

### Correlation between the serum IL-6 level and clinical outcome

Among serum inflammatory cytokine levels examined, the serum IL-6 level was significantly increased in patients with PD at day 49 compared with that at day 1 (p = 0.020; 95% CI, -37.74 to -6.66; Fig 4C). In addition, the level of IL-6 was higher in patients with PD than those with SD at day 49 (p = 0.0018; 95% CI, 13.025 to 39.531; Fig 4C).

### Immunohistochemical analysis of biopsied tumor specimens

**Fig 5:**
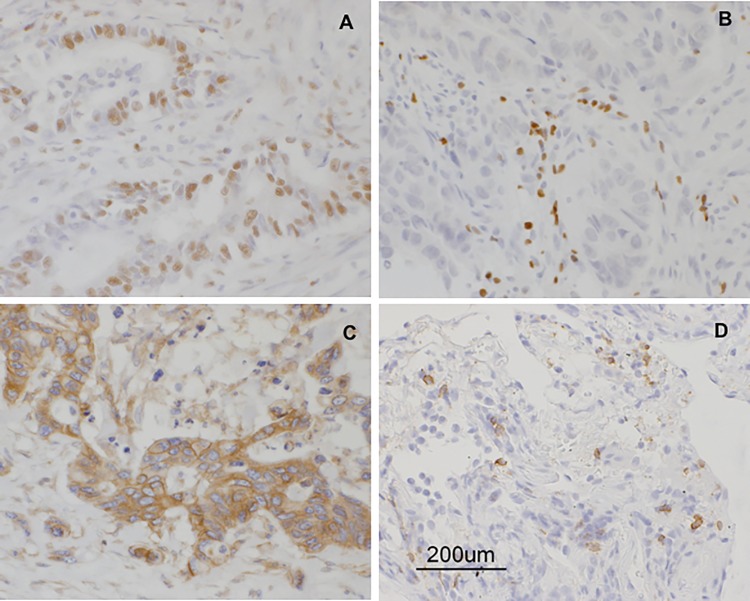
Immunohistochemical analysis of biopsied tumor specimens from patients. (**A**) RNF43-positive cells in the tumor from patient KU-2. (**B**) Foxp3-positive lymphocytes in the tumor from patient KU-5. (**C**) HLA-Class1-positive cells in the tumor from patient KU-10. (**D**) CD8-positive lymphocytes in the tumor from patient KU-10 after treatment.

**Fig 6:**
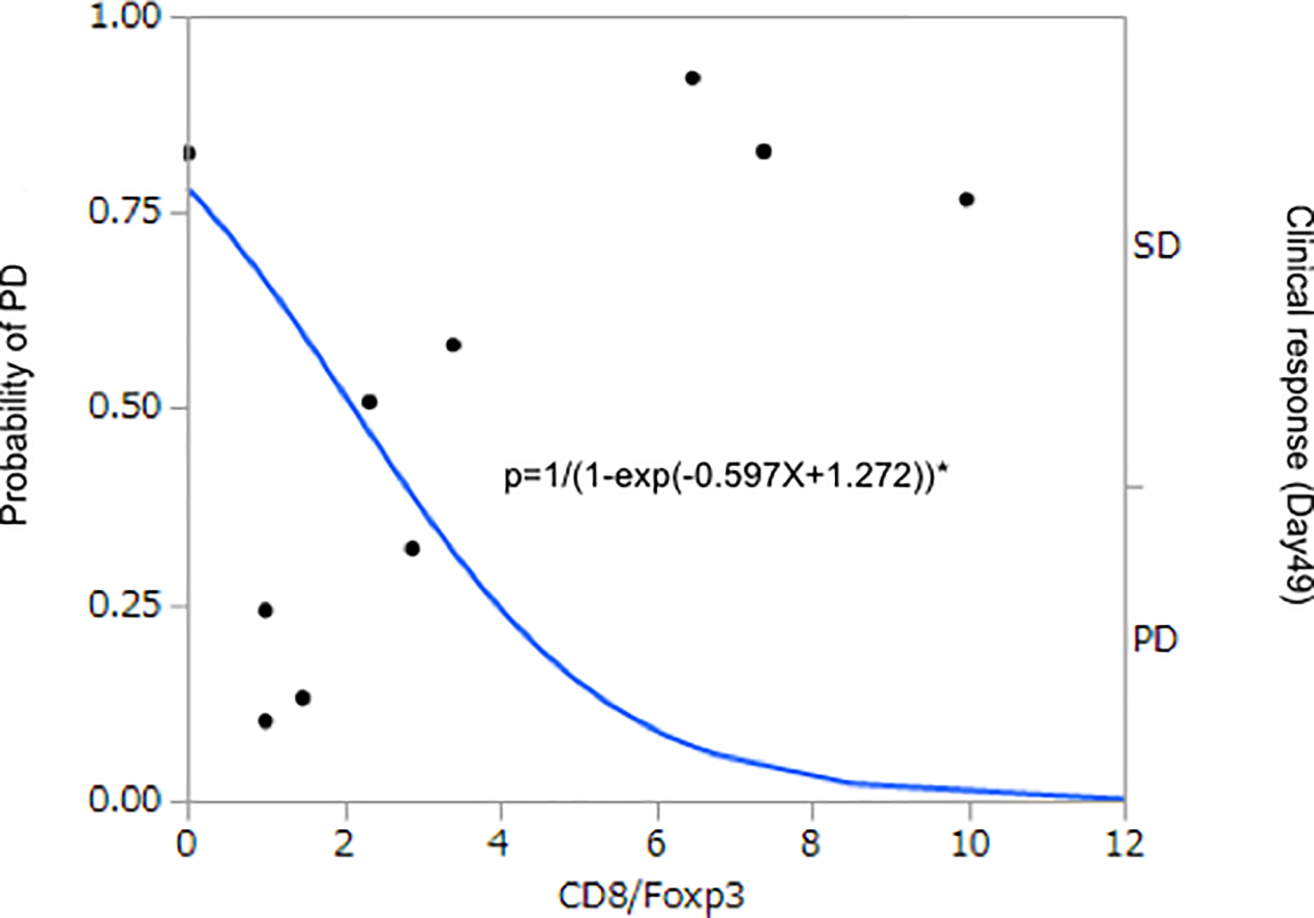
Logistic model applied to determine the predictive ability of CD8/Foxp3. PD/(PD+SD) was regressed on CD8/Foxp3. The regression parameter was estimated to be -0.390 (P = 0.165), and odds ratio was 0.677. Dots denote the clinical response, SD or PD. Solid line is predicted probability of PD. *, logistic-regression model. P, Probability.

Immunohistochemical analysis of RNF43 expression in tumor biopsies obtained before the trial demonstrated abundant expression of RNF43 in 8 of the 10 patients (Table 4 and Fig 5). The tumors in the remaining 2 patients (KU-1 and KU-6) demonstrated relatively lower RNF43 expression levels and were evaluated clinically as PD (Tables 1 and 4). Additionally, very low numbers of CD3^+^, CD4^+^, CD8^+^, and Foxp3^+^ lymphocytes were noted (Table 4). In patient KU-10, the tumor was immunohistochemically evaluated before and after the trial. Tumor biopsy was collected on Day 230 and showed that the population of CD8^+^ lymphocytes in the tumor increased compared with that prior to initial treatment (Table 4 and Fig 5). The logistic model was applied to determine the ability of CD8/Foxp3 ratio as a biomarker to correlate the CD8/Foxp3 ratio with the efficacy of this treatment (PD/PD+SD). The odds ratio was estimated to be 0.667, with the 95% confidence interval between 0.335 and 1.080 (Fig 6).

**Table 4:** Characteristics of RNF43 and immunohistochemical analysis in tumor biopsies.

Patient No.	mRNF43 /HCT15	RNF43 mutation*	anti- RNF43	Foxp3[/HPF]	HLA- class1	CD8[/HPF]	CD4[/HPF]	CD8/Foxp3
KU-1	1	-	-	0	+	0	0	1
KU-2	11	-	+	25	+	37	110	1.48
KU-3	54.8	-	+	5	+	37	8	7.4
KU-4	1.3	-	+	6	+	60	NA	10
KU-5	1.5	-	+	61	+	208	135	3.41
KU-6	1.4	-	-	0	+	0	0	1
KU-7	2.4	-	+	4	+	0	0	0.002
KU-8	6.1	-	+	15	+	35	74	2.33
KU-9	13.2	-	+	59	+	170	339	2.88
KU-10	1.6	-	+	2	+	13	13	6.5
KU-10**		-	+	51	+	28	83	0.55

*Frameshift mutations encoding p.Gly659fs and p.Arg117fs

Immunohistochemistry was performed on paraffin embedded tissue from tumor biopsy specimens in each patient before the treatment and after the treatment in KU-10**. NA, not available

### DNA sequencing of *RNF43* transcripts from tumors

Because of the paucity of patient's tumor samples for genomic sequencing in our clinical study, we performed *RNF43* transcript analysis by RT-PCR of tumor mRNA focusing on two recurrent hotspot mutations of G659fs and R117fs, accounting for 41.7% to 48.0% and 8.3% to 12.0% of *RNF43* frameshift mutations identified in colon cancer and endometrial cancer [26]. The cDNA sequencing results showed no frameshift mutations in all of ten samples (Table 4).

## Discussion

We conducted a phase I clinical trial of cellular immunotherapy consisting of intravenously administered DAKs and subcutaneous vaccination of DCs followed by systemic administration of low-dose IL-2, combined with administration of low-dose CPA in patients with advanced, solid tumors. The results showed that the treatment was safe. Several previous studies suggested that the combination of DC vaccination with adoptive T cell transfer was superior to individual administration [27–29]. Regulatory T cells were thought to dampen T-cell immune responses to TAAs and comprised the main hurdle to successful immunotherapy and active vaccination [30]. CPA was reported to eliminate Tregs without adversely affecting vaccine-induced CD8^+^ T-cell function in mice [31]. Our results also showed that CPA reduced the frequency of peripheral blood Tregs in association with good clinical responses and antitumor immunity. A single, pre-conditioning dose of 300 mg/m^2^ CPA prior to immune cell administration was suggested to enhance antitumor immunity induced by multiple vaccinations using five tumor-specific antigen peptides [15]. CPA has long been used as treatment for cancer, with proven safety and is cost-effectively eliminates Tregs. The molecular basis explaining the selectively targeting of Tregs by low-dose CPA is unclear. Previous data demonstrated that the intracellular ATP level were much lower in Tregs compared with those in conventional T cells or other cell types [32]. The low levels of ATP attenuated glutathione synthesis, leading to decreased CPA detoxification and increasing sensitivity of Tregs to low-dose CPA [32].

Additionally, the administration of low-dose IL-2 maintained or further increased the number of antigen-specific CD8^+^ T cells [33]. IL-2 also increased the clinical response of the administered DC vaccine [34] and was not detrimental to the functional activities of vaccine-primed CD8+ T cell effectors despite the expansion of Tregs, because IL-2-boosted Tregs fraction was functionally modulated to a Th1-like phenotype in the vaccinated patients [35].

In this dose-escalation study of DAKs, all patients administered with both dose levels tolerated treatment well, showing toxicities less than Grade 2. We considered that the higher dose would be superior to the lower dose in terms of efficacy. Patient KU-10, who received the higher dose of DAKs, was evaluated as showing SD on day 49, followed by a transient increase in partial tumor mass, but subsequently showed a successive decrease in tumor size, thus demonstrating long-term objective response. Combined with pathological findings at the tumor site, tumor-specific immune responses were considered to be induced by the administration of immune cells.

The simultaneous administration of DC and IL-2 may have boosted the function of a relatively lower number of DAKs in vivo [36,37]. Conversely, the robust proliferation of DAKs was associated with the subsequent decrease in CD8+ T cell survival, leading to a disruption of the critical, proliferative hierarchy that is necessary to maintain antitumor cell populations over a long term [38]. The low number of DAKs incubated with common γ-chain cytokines such as IL-2, IL-7 and IL-15 in this study resulted in a low number of exhausted T cells (CD45RA^+^CD62L^-^) (Table 2A) [38].

To investigate the immunological changes and predictive biomarkers required to select patients for this treatment, PBMCs and serum from 10 patients were examined before and after administration of the immune cells. First of all, RNF43 peptide-reactive CD8^+^ T-cell responses correlated well with good clinical responses. Secondly, an association was observed between peripheral serum IL-6 level as well as frequency of Tregs with clinical response. Logistic regression analysis was performed, between pretreatment immunological parameters and clinical responses to identify the predictive factor. Although no significant results were observed in blood samples due to the small number of cases analyzed in this study, the odds ratio for clinically resistant patients with higher IL-6 ratios was 0.772 (0.54–1.11, 95% confidence interval, p = 0.157). Previous studies also suggested that serum IL-6 was a useful predictor for cancer immunotherapy [39,40]. IL-6 was considered to stimulate inflammatory cytokine production, tumor angiogenesis, and the tumor macrophage infiltrate as well as inhibit the differentiation of localized T cells to effector cells [40]. ATP also acts as the second messenger during inflammation and stimulates IL-6 synthesis. Extracellular ATP was able to activate Tregs and increase their suppressive capacity [41]. Thus, the patients with PD who showed higher serum levels of IL-6 without an increase in Th17 cells might be expected to augment serum ATP concentrations as a warning sign, followed by increased intracellular ATP levels in Tregs and decreased sensitivity of Tregs to low-dose CPA [35], resulting in tumor progression.

Moreover, the immunohistological CD8/Foxp3 ratio and RNF43 expression prior to the administration of immune cells might be a useful predictor of the clinical response, and our results suggested that an adequate balance between CD8^+^ T cells and Tregs is necessary to obtain strong antitumor effects.

Recent reports showed that *RNF43* encoded an E3 ubiquitin ligase that negatively regulated Wnt signaling and played a crucial role in the development of various cancers [42–44]. Somatic mutations of *RNF43* in 14% to 19% of colorectal, pancreatic, and endometrial cancers were recently reported [26,44]. These showed frameshift mutations encoding G659fs and R117fs, constituting insertions or deletions of 1 bp in homopolymeric tracts of the *RNF43* gene. *RNF43* was mutated in approximately 80% of microsatellite instability colorectal tumors and 4.8% of microsatellite stable (MSS) tumors [26]. Although RNF43-based immune therapies suggested its clinical potential [45,46], the precise role of *RNF43* mutation in tumor cells in immunotherapy remains to be clarified. We targeted tumor cells expressing relatively high levels of RNF43 without major gene mutations, possibly included in MSS tumors. The function of ZNRF3/RNF43 is counteracted by R-spondin; R-spondin binds to LGR4/5 and ZNRF3/RNF43 and induces ubiquitination and degradation of ZNRF3/RNF43. To achieve high and sustained Wnt/β-catenin signaling, cancer cells need to overcome this strong negative feedback control, which can be achieved through mutations of ZNRF3/RNF43 or translocations/overexpression of R-spondin [47]. As we could detect no major *ZNRF3/RNF43* gene mutations in our patients' tumor, the latter might be functional in our cases. Although the significance of higher RNF43 expression in our cases still unknown, tumor specific expression of RNF43 was considered to be a reliable target for immune therapy. Further examination is required to prove this hypothesis.

A deeper understanding of immune responses in the tumor microenvironment is required for recently proposed combination therapies to provide a survival benefit towards a greater number of cancer patients [48,49]. Namely, non-immunogenic tumor microenvironments that are resistant to immune-checkpoint therapy may require combination therapies to create an immunogenic tumor microenvironment [48,49]. Our new combination treatment was feasible in patients with advanced solid tumors, especially MSS tumors that were refractory to standard therapy, and may have the potential to induce an immunogenic tumor microenvironment. A clinical study of combined immune cell therapy with immune-checkpoint inhibitors for patients with refractory colorectal cancer is being planned [49].

In conclusion, we demonstrated that RNF43 peptide-related immune cell therapy combined with low-dose cyclophosphamide and IL-2 was safe, and that the reduction of peripheral blood Tregs and the immune response correlated well, resulting in good clinical response in patients with advanced solid tumors. Further immunological studies and a role of *RNF43* mutation and translocations/overexpression of R-spondin in the progression of colon cancer is planned, to explore valid biomarkers that predict clinical efficacy of immune therapy.

## Supporting information

S1 File CONSORT checklist. (PDF)

S2 File Original trial protocol in Japanese. (PDF)

S3 File Original trial protocol in English. (PDF)

S4 File Case Report form (Immunological results). (PDF)

S5 File Case Report form (Blood study results). (XLSX)

S1 Fig Immunophenotypes of DAKs analyzed by flow cytometry. DAKs were analyzed after gating lymphocytes using forward scatter and side scatter. Representative flow plot of CD3+CD4+ and CD8+ T cells are separated into naïve (CD45RA+CD62L+), effector memory (CD45RA-CD62L-), central memory (CD45RA-CD62L+) and terminal effector (CD45RA+CD62L-).
(TIF)

S2 Fig Immunophenotypes of DCs analyzed by flow cytometry. The cell surface phenotypes of each gated DCs population were analyzed after gating monocytes using forward scatter and side scatter by flow cytometry. Matured CD11c+DCs were indicated in each panel expressing high levels of CD40, CD80, CD83, CD86, HLA-ABC and HLA-DR.
(TIF)

S3 Fig Clinical responses in patient KU-4 with uterine cervix cancer after completing this trial. Although the size of the tumor mass in the pelvis and the levels of the two tumor markers decreased slightly, bilateral ureterohydronephrosis improved after this trial.
(TIF)

S4 Fig Clinical responses in patient KU-5 with esophageal cancer after completing this trial. The red arrows indicate the metastasized tumors in the mediastinum. They were enhanced by positron emission tomography-computed tomography (the bottom of the image before CPA). The target lesions did not change during the observation period. The serum level of tumor marker of squamous cell carcinoma (SCC) decreased and was maintained at a reduced level after both treatments.
(TIF)

S5 Fig Identification and proportional change of Tregs in peripheral blood samples between Day1 and Day49. a) PBMCs were stained for CD4, CD25, Foxp3 and analyzed by means of flow cytometry. Tregs are illustrated by costaining of CD4+, CD25 high and Foxp3+. b) The frequency of peripheral blood Tregs in all patients decreased significantly between the pre-CPA time point and Day 6 (p = 0.013) or Day 49 (P = 0.009).
(TIF)
