# Supplementary material for: A phase I clinical trial of RNF43 peptide-related immune cell therapy combined with low-dose cyclophosphamide in patients with advanced solid tumors
Source: PLoS One. 2018 Jan 2;13(1):e0187878. doi: 10.1371/journal.pone.0187878 (PMC5749706; doi:10.1371/journal.pone.0187878)
Supplement: S2 File — (PDF) [file pone.0187878.s002.pdf]

実施計画書

RNF43 ペプチドパルス樹状細胞ならびRNF43 ペプチド特異的活性化リンパ球を用いた進行固形腫瘍患者に対する強化養子免疫療法　第一相臨床研究

Version 3.0

試験責任医師
九州大学病院
先端分子・細胞治療科　教授
谷　憲三朗

1．課題名

RNF43ペプチドパルス樹状細胞ならびRNF43ペプチド特異的活性化リンパ球を用いた進行固形腫瘍患者に対する強化養子免疫療法第一相臨床研究

2．目的

強力な抗原提示細胞(professional antigen presenting cell: professional APC)である樹状細胞に腫瘍抗原ペプチドである Ring finger protein (RNF) 43 ペプチドを in vitro で提示させ末梢血リンパ球と共培養することにより RNF43 ペプチド特異的活性化リンパ球（活性化リンパ球）を誘導する。その後この活性化リンパ球および RNF43 ペプチドパルス樹状細胞を患者に投与し、本治療法の安全性および抗腫瘍免疫誘導効果の有無を in vitro 検査ならびに臨床効果の観点から検討する。

3．背景

近年の集学的治療法の進歩により、癌の治療成績は年々向上している。しかし、手術療法、化学療法、放射線療法等既存の一般的治療法に対する治療抵抗例や再発例などに対しては有効な治療法は無く、症状緩和療法主体の対症療法に留まっているのが現状である[1)]。したがって、これら現在の治療法では治癒が望めない症例に対して、新しい治療法を開発することが社会的にも強く要請されておりその候補として免疫療法が挙げられる。

RNF43 は、23,040 個の遺伝子情報からマイクロアレイ法にて大腸癌に対して高発現している新規遺伝子として同定された。22 番染色体の短腕にコードされ、783 個のアミノ酸から成る。RNF43 は腫瘍細胞の核および細胞質に局在し、一部分泌タンパクの特徴も有する。正常細胞には発現しておらず、大腸癌の 85%以上に高発現しており[2)]、DNA マイクロアレイにて膀胱癌、非小細胞性肺癌などにも高発現を認めている（古川ら私信）。また、機能的にも腫瘍増殖に関与している可能性が示唆されている[2)]。田原らは RNF43 由来で HLA-A*0201 に結合能を持つペプチド(ALWPWLLMA) および HLA-A*2402 に結合能を持つペプチド(NSQPVWLCL)を用い、CD8 陽性 T 細胞を刺激し、ペプチドをパルスした標的細胞に強力な細胞障害活性を認める CTL クローンを樹立することができた。この CTL クローンは、RNF43 を内因性に発現しかつ HLA-A*0201 または HLA-A*2402 を保持している大腸癌細胞株に対して、それぞれ強い細胞障害活性を示した。さらに

細胞障害活性の特異性は、cold target inhibition assay および blocking assay によって確認された[3)]。以上の知見より、RNF43 は免疫原性を有しており、RNF43 を認識・障害する CTL を誘導するがんワクチン療法に応用できる可能性が示唆された。

これまでのペプチドを用いた抗腫瘍免疫療法のうちで安定した成績を報告したものは少ないが、その原因として癌部において制御性T細胞等による投与した活性化免疫細胞の非活性化[4)]があげられるが、近年、シクロホスファミドの投与により腫瘍免疫に対し抑制的に働く制御性T細胞が特異的に排除できることが報告され、 それにより免疫細胞療法の効果が増強される可能性が示唆されている[5)]。更にもう一つの原因として生体に投与した活性化免疫細胞クローンの消失が示唆されており[6)]、抗腫瘍免疫療法が有効であった症例では投与した活性化免疫細胞クローンが生体内にて長期間維持できていたとの報告もある[7)]。このような長期間生体内に存続し腫瘍抗原に対する強力な反応性を示すCTLを得るため、effector細胞およびmemory細胞の誘導が必要である。ナイーブT細胞が抗原提示細胞による抗原刺激を受ける(initiation phase)と抗原特異的なT細胞クローンは顕著な増殖(clonal expansion)の後effector細胞となるが、その後活性化したeffector T細胞の大半はアポトーシスを起す(contraction phase)[8)]。抗原に対して高親和性のT細胞受容体を持つ一部のT細胞は、2回目以降の抗原刺激に対してより強力な反応性を獲得したCD44^{high} memory T細胞として生存が維持される(maintenance phase)。これらのeffector細胞およびmemory細胞の維持にIL-7及びIL-15が必要であることが報告されている[9)]。またマウスモデルにおいて樹状細胞の単独投与よりも活性化リンパ球を投与し、その後樹状細胞を追加投与することで、活性化免疫細胞クローンが生体内にて長期間維持でき、樹状細胞療法単独よりも強力な抗腫瘍免疫の誘導が可能であったとの報告がなされている[10)]。

4. これまでの成果とこれからの期待

我々はこれまでに本邦において初めての悪性腫瘍に対する遺伝子治療臨床研究として、東京大学医科学研究所附属病院において「第 IV 期腎癌に対する免疫遺伝子治療臨床研究」を完遂した経験を有している[11)]。我々の研究室では以下のような本臨床研究推進に対する基盤技術を有しており、本臨床研究の完遂は十分に可能であると考えている。一方 RNF43 に関しては東京大学医科学研究所

において進行大腸癌に対する新規癌関連抗原遺伝子 RNF43 由来 HLA-A*2402 拘束性エピトープペプチドを用いた腫瘍特異的ワクチン療法（第 I 相臨床研究）が行われ、安全性が確認されている。今回申請する進行固形腫瘍患者に対する強化養子免疫療法第一相臨床研究に関しては体外においてあらかじめ RNF43 ペプチドパルス樹状細胞を用いて誘導した強力な活性化リンパ球を体内に戻す際にシクロホスファミドを用いて制御性 T 細胞を排除し、さらに活性化リンパ球を維持活性化させる目的で RNF43 ペプチドパルス樹状細胞、IL-2 を投与する。この様な臨床研究は他に報告はなく、新規性の高い抗腫瘍効果を十分に期待し得る方法であると考える

4.1.　樹状細胞の分離法

非公開情報のため本文削除

4.2.　活性化Tリンパ球の誘導法

テーラーメード・マルチペプチドパルス成熟樹状細胞を用いた癌免疫療法臨床研究を行っており、本研究の実施に付随したin vitroでの抗腫瘍免疫学的解析にも習熟している。例えば以前より腫瘍崩壊産物貪食樹状細胞と末梢血由来Tリンパ球の共培養による活性化リンパ球の誘導およびその細胞障害能の検討、パルス成熟樹状細胞と末梢血単核球の共培養による誘導細胞の表面マーカーの検討などを行ってきた。その結果、3回以上の抗原提示が有効な活性化リンパ球の誘導には必要なこと、ペプチドパルス成熟樹状細胞と末梢血単核球の共培養によりCD8陽性細胞を有意に増殖させることが可能であることを確認している。

4.3.　シクロホスファミドを用いた制御性T細胞除去による抗腫瘍効果の増強

我々はこれまで血液悪性腫瘍に対する一般的治療法としてシクロホスファミドの安全な投与には多くの経験を有している。近年、シクロホスファミドの投与により、腫瘍免疫に対して抑制的に働く制御性 T 細胞が特異的に排除されることが報告され、 その後の免疫細胞療法の効果が増強される可能性が示唆されている。さらにこの効果は少量のシクロホスファミド投与にて得ることができ[12)]、活性化リンパ球4～7日前に投与するのが有効であることが判明している[13)]。このような背景の元、制御性 T 細胞除去目的での低量シクロホスファミドの使用は十分に可能である。

5.安全性

5.1.　発熱物質・微生物の混入

試薬に関してはすべて臨床グレードもしくはそれに準ずるものを用いる。また培養の一連の操作は清潔区域(九州大学病院セルプロセッシングルーム)で実施する。樹状細胞の誘導にはGM-CSF、IL-4、TNF-α、KLH、OK432を、活性化リンパ球の誘導には、IL-2、IL-7、IL-12、IL-15を培養に用いる。これらは免疫賦活物質として知られている。また輸注した活性化リンパ球を体内に長期間維持させるため、IL-2の全身投与を行う。IL-2は我が国でも製薬として認可を受けており多くの投与実績がある。副作用としては発熱が最も多いが非ステロイド系の消炎剤投与により十分な対処が可能である。その他の副作用として浮腫等の水分貯留、うっ血性心不全、精神症状、肝、腎機能障害、自己免疫反応な

どの報告があるが、今回用いる投与量は通常投与量の半分量であり注意深い観察を行いながら十分投与可能と考えられる。OK432も消化器癌患者及び肺癌患者における癌性胸・腹水や他剤無効の頭頸部癌(上顎癌，喉頭癌，咽頭癌，舌癌)及び甲状腺癌などの製薬として認可を受けており多くの投与実績がある。副作用としては過敏症、ショック、間質性肺炎、急性腎不全などの報告がある。GM-CSFは米国等の海外で同種及び自家末梢血幹細胞採取時の単独投与による動員や化学療法後の白血球増加を目的とした急性骨髄性白血病製剤として認可を受けており数多くの投与実績があるが、副作用として浮腫等の水分貯留、呼吸器障害、上室性不整脈、肝・腎機能障害などの報告がある(医療用医薬品添付文書より)。また、IL-4、IL-7、IL-12、IL-15、TNF-α、KLHは国内外で製薬としての認可を受けていないため研究用試薬（GMPグレード）をin vitroにて使用することとなる。KLHはマウスアレルギー性気道収縮モデル作製に用いられており[14)]アレルギー反応誘導の可能性があるが、悪性骨髄腫や膀胱癌に対する治療に用いられ、接種局所の発赤、疼痛、硬結は認めているもののGrade３以上の有害事象の報告はない[15)]。また原発性肝癌や腎癌においてKLHを用いて樹状細胞の誘導を行い患者に投与した報告では、副作用発生の報告はない[16,17)]。また、これらの薬剤、サイトカインで培養した細胞は3回の洗浄後、患者に輸注および皮下注することとなる。つまりリンパ球を500mlの生理食塩水で洗浄すると、細胞ペレットは2ml以下であり、1回の洗浄で混入物質の濃度は1/250以下、3回の洗浄で$1/10\times10^7$以下となる。また樹状細胞を10mlの生理食塩水で洗浄した場合、細胞ペレットは100μl以下であり一回の洗浄で混入物質の濃度は1/100以下、3回の洗浄で$1/10^6$以下となることがこれまでの報告から示されている。従っていずれの薬剤およびサイトカインも患者に投与される血液中ではほとんど無視できる濃度になっているものと考えられる。

5.2.　自己免疫反応誘導の可能性

樹状細胞に提示された自己抗原による自己反応性 T 細胞の活性化による自己免疫疾患誘導の可能性が指摘されているが、元々胸腺での negative selection を介して自己反応性の T 細胞は取り除かれており可能性は低いものと考えられる。またメラノーマに対する非破壊的骨髄前処置後の TIL 療法により尋常性白斑やぶどう膜炎発症の報告があるが、尋常性白斑は臨床上特に重篤な副作用ではなく、ぶどう膜炎に対してもステロイド点眼にて改善し、視力障害も生じて

いない[18]。なおこれらの有害事象はメラノーマに対する免疫療法に際して特有なものであると考えられている。また培養に用いた血清製剤やサイトカイン等への抗体産生の危険はあるが原疾患の重篤性から無視できる範囲と考えられ、実際にヒトへの in vivo 投与を行った文献を含めて現在のところそのような自己免疫疾患誘導を報告した文献はない。

また、我々が現在まで行ってきたテーラーメード・マルチペプチドパルス成熟樹状細胞を用いた癌免疫療法臨床研究における各種腫瘍抗原パルス樹状細胞投与においても副作用は出現していない。しかしながら、安全を期すために細胞ならびに IL-2 の患者への投与は入院観察下で行い、投与終了後も患者には定期的な外来受診をおこなってもらい、副作用発現のモニターならびに副作用発現時の早急な対処を十分に可能な状態にして臨床研究を進める。

5.3.　細胞数

当臨床研究にて患者への投与予定樹状細胞数は１回あたり 1×10^7 個である。これまでの報告では樹状細胞の皮下投与では 1×10^8 個の細胞が安全に投与されており[19]、また当科において行われたテーラーメード・マルチペプチドパルス成熟樹状細胞を用いた癌免疫療法臨床研究でこれまで 1×10^7 個の細胞投与を行った患者で特に有害事象は発生しなかった。本研究でも同数の樹状細胞数を投与予定であり安全に投与し得るものと考えられる。

本療法にて抗原として使用する RNF43 は正常細胞には発現が低いため、活性化リンパ球により正常組織が障害を受ける可能性は極めて少ないと考えられるが、これ迄に活性化リンパ球と樹状細胞との投与が同時に行われたことはない。

これまでに同種骨髄移植後の再発慢性骨髄性白血病に対する同種ドナーリンパ球輸注療法の報告にて 6×10^7 個(60kg)の CD3+細胞の注入にて Grade２､6×10^8 個(60kg)の注入にて Grade３の GVHD の発症を認めたとの報告がある[20]。一方自己活性化リンパ球療法においては一般に 1×10^9 個のリンパ球投与が重篤な有害事象を起すことなく投与されている。本臨床研究ではこれらの細胞投与を参考に、安全性を期して 5×10^7 個と 2×10^8 個の 2 段階漸増研究を行う。

5.4.　シクロホスファミドの前投与による副作用

シクロホスファミドの投与による副作用として一般的に骨髄抑制、出血性膀胱炎等があげられるが、本臨床研究では従来の化学療法の投与量(約 750mg/m^2)

よりも少量投与予定(300mg/m^2)であり、重度の副作用発現の可能性は低いと考えられる。

5.5.　アフェレーシス

本処置に由来する副作用については全身倦怠感（30%前後）のほか、四肢のしびれ、めまい、吐き気、嘔吐など血管迷走神経反射や一過性の脱水による症状がみられる。これらに対してはアフェレーシスに習熟した医師が ECG モニター監視下で速やかに対処できる体制にて行う。また血球採取後に血小板数の減少が認められることもあるため終了後適宜血小板数をチェックし、採取前値への回復を確認する。

5.6.　RNF43 ペプチドの安全性

東京大学医科学研究所外科(責任医師：田原秀晃、金本彰)にて HLA-A*2402 を有する進行大腸癌患者に対して、HLA 拘束性 RNF43 由来エピトープペプチドを用いた腫瘍特異的ワクチン療法(第 I 相臨床研究)が行われ、8 例の患者が参加し、完遂された。関連する有害事象は接種部位の発赤硬結のみで Grade３以上の有害事象は生じていない。第 I 相臨床研究の主目的であるこの臨床研究の安全性は確認できている。

5.7.　樹状細胞の安全性

当科にてテーラーメード・マルチペプチドパルス成熟樹状細胞を用いた癌免疫療法臨床研究を行っており現在まで 5 名の患者に接種を終了した。有害事象としては接種部位の発赤を認めたのみで、Grade３以上の有害事象は認められなかった。

6. 細胞作製基準

誘導する樹状細胞や活性化リンパ球に関しては作成ロットごとに培養上清の病原性チェックと各種免疫細胞表面マーカーのチェックを行ない、前者においては病原性のないことが判明した細胞のみを患者に投与する。また後者においては免疫細胞表面マーカーの情報と各臨床データ（各種免疫反応検査、腫瘍縮小効果、有害事象の有無など）との関連性について比較検討する。

7. 計 画

【概要】

手術による根治や、抗癌剤・放射線など既存の治療での効果が望めず予後が極めて不良であると考えられる進行固形腫瘍患者のうちで HLA-A*0201 または HLA-A*2402 陽性が確認されており、かつ腫瘍細胞または腫瘍組織に RNF43 が発現している患者を対象とする。病名や病期、病態に対して十分な説明を受けていることを原則とし、本研究スケジュールや副作用を理解し他の代替治療の可能性なども議論し、本研究は必ずしも効果が得られない可能性を十分納得した上で、文書による同意を取ることを原則とする。

【症例数】

対象患者数は、活性化リンパ球ならびに 3 回の DC 接種後 4 週目の評価を行えた症例を評価可能症例として計 10 名とする。

【経費】

この臨床研究に用いる細胞製剤の調製および投与に関連する経費、臨床研究期間内の治療および入院費は患者請求しない。なお臨床研究期間内とは細胞投与を行う期間とする。その臨床研究期間外の原疾患の症状判定を目的とする通常検査（レントゲン、CT スキャンなどの画像検査、血液検査など）や外科的治療やそれに伴って生じる入院費に関しては通常の支払い方法（例えば健康保険など）となる。

7.1. 投与スケジュールと観察スケジュール

投与スケジュールは、Day1 の化学療法（シクロフォスファミド）投与後、Day6 から細胞製剤の投与を２週間に渡り計３回投与を行なう。初回 Day6 に活性化リンパ球および樹状細胞の投与を行い、同時に IL-2 接種を３日間連日で行う。その後２回目 Day13、３回目 Day20 に樹状細胞の投与を行い、同時に IL-2 接種を３日間連日で行う。

当臨床研究で投与予定の細胞数は活性化リンパ球がレベル１：5×10^7 個、レベル２：2×10^8 個、レベル１、２ともに樹状細胞数が 1×10^7 個である。活性化リンパ球は 2%患者血清含有生理食塩水 100ml に懸濁し、約 1 時間をかけ点滴静脈内投与する。樹状細胞は生理食塩水 1.0ml に懸濁し皮下（もしくは皮内）投与する。また IL-2 は１回あたり[35×10^4IU/350μl 注射用蒸留水]を皮下投与する。シクロホスファミドは 300mg/m^2 を生理食塩水液 500ml に加え点滴静脈内投

与(約2～3時間での投与)する。

7.2.　細胞調製スケジュール

非公開情報のため本文削除

7.3.　適応基準

九州大学病院先進医療適応評価委員会にて以下の条件をすべて満たすと認められた患者を対象とする。

1)生存期間を延長する治療法がない進行固形腫瘍患者で、かつ一般状態評価が良好である症例（ECOG のPerformance Statusにて0～1)。

2) HLA typing 検査によりHLA-A*0201またはHLA-A*2402陽性が確認されており、かつ腫瘍細胞または腫瘍組織にRT-PCR法にてRNF43の発現がRNF43発現陽性細胞株と同等以上認められる患者。

3) 前治療（化学療法、免疫療法、放射線療法、外科手術など）からのwash-outにはシクロホスファミド投与日より 4 週間以上を必要とし、前治療の効果や有害事象による影響を持ち越していないと判断される患者であること。なお、採血、アフェレーシス前2週間は化学療法、放射線療法を行わない。

4)アフェレーシスを行いうる患者。

WBC ＞ 3,000/mm^3

Hb ＞ 8.0 g/dl

Platelet ＞100,000/mm^3または＜600,000/mm^3

最高血圧90mmHg以上

5)WHO-RECIST判定における評価可能病変の存在する患者。

6)登録時より3ヶ月以上の生存が期待できる患者。

7)血液、生化学検査にて以下の基準を満たす患者。

WBC ＞ 3,000/mm^3

Hb ＞ 8.0 g/dl

Platelet ＞ 100,000/mm^3

Serum creatinine ＜ 1.5 mg/dl

Total bilirubin ＜ 2.0mg/dl

ASTおよびALT 施設内基準値の上限3倍以下

8）B 型肝炎ウィルス抗原陰性かつ C 型肝炎、ATL、HIV ウィルス、梅毒陰性の患者。

9）20 歳以上 70 歳以下の患者。

10）患者本人から文書による同意が得られている患者。

【除外基準】

以下のいずれかの条件に該当する患者は対象としない。

1）重篤な基礎疾患（活動性重症感染症、循環器障害、呼吸器障害、腎障害、免疫不全、血液凝固能障害など）を有する患者。

2）脳転移を認める患者。（ガンマナイフ後などで増大傾向のない患者をのぞく。）

3）重篤なアレルギー疾患あるいは活動性の自己免疫疾患のある患者。

4）免疫抑制剤を使用中の患者。

5）妊婦、授乳婦又は妊娠している可能性のある患者および妊娠を希望している患者。

6）培養に際して T リンパ球の増殖不良により規定数以上の活性化リンパ球が得られない患者。

7）アフェレーシス採取単核球より誘導した樹状細胞の増殖不良により規定数以上の細胞数が得られず、再アフェレーシスもできない患者。

8）その他、研究責任医師又は研究分担医師が本研究の対象として不適当と判断した患者。

9）活性化リンパ球および誘導樹状細胞の出荷基準を満たさない患者

7.4.　同意方法

別添の「RNF43 パルス樹状細胞ならび活性化リンパ球を用いた進行固形腫瘍患者に対する強化養子免疫療法第一相臨床研究」説明および同意書にて説明を行い、同意書による文書同意を得る。

7.5. 治療の実際

7.5.1. 対象癌患者の選択

本治療の対象患者は、前治療を受け、標準治療法の確立していない段階での進行固形腫瘍患者および既存の治療に抵抗性の進行固形腫瘍患者で、

HLA-A*0201またはHLA-A*2402陽性であり、かつ腫瘍細胞または腫瘍組織において RNF43 が発現しているものとする。これらの判定は以下の方法で行う。

① HLA-A*0201 および HLA-A*2402 発現検査法

HLA-DNA タイピング法は WAKFlow HLA タイピング試薬（別添マニュアル参照）（湧永製薬、広島）を用いて湧永製薬においてタイピングを行う。

② RNF43 発現の検査法

Real-time Reverse Transcription Polymerase Chain Reaction (real-time RT-PCR)法を用いる。生検（経内視鏡的生検、経皮的生検等）により採取した腫瘍細胞または腫瘍組織より total RNA を抽出する。total RNA の一部を RNF43 遺伝子特異的プライマ

FW:CCAGTGTGGTTGTGCCTGAC;

RV:CATGAAGGATCTTCTGTGACCT

を用いて RT-PC 法により cDNA を合成し、SYBR Green I を用いた real-time PCR 法により RNF43 の相対的発現量の解析を行う。また新たな生検が困難な患者については以前に採取保存されているホルマリン固定標本等にても同様の検査を本検査でおこなう。

7.5.2. 治療直前画像診断評価

細胞製剤投与直前に画像診断（コンピュータ断層法（CT）を含む）にて WHO-RECIST 基準に準拠し標的病変の評価を行う。

7.5.3. アフェレーシスまたは採血による末梢血単核球の採取

アフェレーシスまたは採血によって得られた患者末梢血より末梢血単核球を採取する。なお PBMC 採取のための処理血液量は 6000ml（細胞数 1.5×10^{9}）を目標とする。

7.5.4. RNF43 ペプチド

Multiple Peptide Systems, San Diego, CA から購入された GMP grade のペプチドを用いる。所定の方法にて溶解、分注したのちセルプロセッシングルーム内の冷凍庫に施錠の上保存する。所定の冷凍庫（-80℃）に施錠の上保存する。

7.5.5.　患者自己末梢血単核球由来樹状細胞の作成

患者末梢血単核球よりプラスチックプレートを用いて接着性細胞と非接着性細胞を分離し、前者より樹状細胞を作成する。作成した樹状細胞に最終濃度20μg/ml の RNF43 ペプチドをパルスしたものを末梢血単核球との共培養および患者皮下（内）注に用いる。もしくは必要時まで凍結保存する。

7.5.6.　RNF43 ペプチド特異的活性化リンパ球の作成

患者末梢血単核球よりプラスチックプレートを用いて分離した非接着細胞と作成したRNF43ペプチドパルス樹状細胞とを共培養することにより活性化リンパ球を誘導する。RNF43 ペプチドパルス樹状細胞による抗原提示は計 3 回行うものとする。

7.5.7.　RNF43 ペプチド特異的活性化リンパ球の経静脈注

活性化リンパ球は日本薬局生理食塩水にて洗浄を行ったのち生理食塩水100ml に浮遊させ点滴静注する。

7.5.8.　RNF43 ペプチド特異的活性化リンパ球の初期投与量および投与の増量計画

投与する活性化リンパ球数は後述の２段階の容量漸増研究とする。各レベルの投与活性化リンパ球数は以下のように設定する。レベル１は $5x10^7$ 個、レベル２は $2x10^8$ 個である。これらは症例の新しいものより順次に投与レベル１より割り付けていく。どの個人に関しても決められた投与量よりの増量は行わない。この投与方法については以下に詳しく述べる。

7.5.8.表

Level	RNF43 ペプチド特異的活性化リンパ球
レベル１	$5x10^7$
レベル２	$2x10^8$

患者はいずれかのレベルの投与量に割り振る。すべての症例において投与量を規制する毒性（DLT: dose limiting toxicities、NCI CTCAE ver.4 にて Grade3 以上の

有害事象）が生じない限り活性化リンパ球の投与を継続する。レベル１より開始し、5 例を登録し、投与が安全に終了した時点でレベル２へと進む。もし設定した用量レベルの１例で用量を制限すべき毒性が出現した場合、もう 1 例をその投与量で治療し用量規定毒性の再現性の有無を観察する。この追加 1 例において用量規定毒性が観察されなければレベル 2 に進む。もし 2 例で用量規定毒性が観察された場合には研究を中止する。レベル 2 においても 5 例を登録し、投与が安全に終了した時点で完了とする。レベル 1 と同様に設定した用量レベルの１例で用量を制限すべき毒性が出現した場合、もう１例にその投与量で治療し、用量規定毒性の再現性の有無を観察する。もし評価中の投与レベルで 2 例の用量規定毒性が観察された場合はレベル 2 を用量規制毒性とするとともに研究を中止する。1 例のみであれば 5 例が終了した段階で完了とする。但し、両レベルにおいて脱落症例があった際は改めてそこに新しい症例を割り付ける。

7.5.9. RNF43 ペプチドパルス樹状細胞の皮下注

RNF43 ペプチドパルス樹状細胞は必要時まで凍結保存しておき、皮下注前に解凍し、日本薬局生理食塩水にて洗浄を行う。一回 1×10^7 の RNF43 ペプチドパルス樹状細胞を所属リンパ節付近の皮下に接種する。活性化リンパ球輸注時、活性化リンパ球輸注後１週目、活性化リンパ球輸注後２週目の計 3 回投与する。

7.5.10. Interleukin-2 使用について

Interleukin-2(IL-2)は本邦においては血管肉腫、腎癌に対する治療薬として一般に使用されているが今回の臨床研究での使用は適応外使用にあたる。しかしこれまでの報告で TIL 療法においては IL-2 の全身投与は静注したリンパ球を活性化し、抗腫瘍効果を増強することが報告されている [21)]。 IL-2 (proleukin®) は今回体外にて誘導した活性化リンパ球を体内でさらに活性化する目的で使用する。製剤は九州大学病院の無菌室において注射用蒸留水で溶解後、分注し冷凍保存しておく。使用時解凍して皮下接種を行う。なお、適応基準を満たす患者において併発症状(胸水、腹水、腸通過障害、呼吸器や消化出血など)がある場合にはこれらの症状を IL-2 自体が悪化させる可能性も懸念されることから、IL-2 の使用については慎重に検討し、場合によっては本投与を省略することもできる。

7.5.11. シクロホスファミド投与について

シクロホスファミドにより制御性 T 細胞を排除し抗腫瘍効果を高められたことが報告されており[17]、同目的にて使用する。

7.6. 追加投与について

細胞数に余裕があり、担当医師が適切と判断した症例に対しては患者本人の同意を得て、更に活性化リンパ球、樹状細胞の輸注を追加できるものとする。ただしそのデータは本臨床研究自体のデータとしては扱わず参考データにとどめる。なお追加接種を実施する際には、先進医療適応評価委員会に報告、承認を得た後、別途文書にて患者に説明後、同意書を得たうえで実施する。

8. 評 価

8.1. 期間

シクロフォスファミド投与開始日から細胞製剤投与終了後４週間後までを評価期間とする。

8.2. 項目

1) 有害事象の評価：NCI CTCAE（NCI common terminology for adverse event ver.4)（別添）に基づき評価する。
2) 抗腫瘍効果：WHO-RECIST 判定基準に基づき評価する。
3) 一般血液検査（末梢血検査、生化学検査)、腫瘍マーカー（血清 CEA 値など事前のスクリーニング検査によりその腫瘍で上昇している腫瘍特異的マーカー)、尿検査、便潜血
4) 生存期間の評価：臨床試験評価期間終了後も３年間は追跡調査を行う。
5) 免疫反応の評価：

①遅延型アレルギー（ＤＴＨ）反応：RNF43 ペプチドに対する遅延型アレルギー（ＤＴＨ）反応の変化の有無を臨床研究前後で検討する。陰性コントロールとして生理食塩水を、陽性コントロールとして精製ツベルクリンを用いる。

②細胞表面マーカー検索：in vitro にて誘導した樹状細胞およびリンパ球

の各種表面マーカーを検索し、樹状細胞においてはその成熟度、リンパ球においてはnaïve, memory, effectorなどのphenotype分画評価を行う。

③細胞内サイトカイン測定:誘導した活性化リンパ球ならびに細胞製剤投与前後の末梢血単核球の細胞内サイトカイン（INFγ，IL-2，TNF-α）をRNF43 peptide 刺激の有無にて測定することにより抗原反応による細胞の活性化を検討する。

④サイトカイン測定:患者血清中のサイトカイン(IL-1β，IL-2，IL-4，IL-5，IL-6，IL-8，IL-10，TNF-α，INF-γ，IL-12p70など）を測定し、細胞製剤投与前後の変化を解析する。

⑤細胞傷害性T細胞(Cytotoxic T Lymphocyte: CTL)活性の評価：細胞製剤投与前後の患者末梢血リンパ球をpeptideと混合培養し、RNF43ペプチド特異的CD107a/b陽性T細胞の変化を評価する。

⑥制御性T細胞（regulatory T cell: Treg）測定：細胞製剤投与前後の末梢血リンパ球を用いてシクロホスファミドによるCD4+CD25+Foxp3+制御性T細胞の排除有無の評価を行う。

9．評価指標（エンドポイント）

1) プライマリー・エンドポイント：
本研究の安全性ならびに有害事象の検討。

2) セカンダリー・エンドポイント：
全生存率、抗腫瘍効果（臨床的効果)、患者の免疫学的評価。

10.有害事象への対処

10.1. 有害事象の記録

主治医は強化養子免疫療法との因果関係の有無を問わず、ICH ガイドラインに定める有害事象については全てを記録する。有害事象の有無と程度を National Cancer Institute-Common Terminology for adverse event version 4 (NCI CTCAE ver.4)を基準に判定する。また、関連性の推測も同時に行う。研究中ならびに最終細胞製剤投与後４週以内にGrade3以上の非血液学的検査異常、またはGrade4の血液学的検査(NCI-CTCAE ver.4の臨床検査項目に準拠)異常が出現した場合および死亡、ICH E2A および E2D ガイドラインに定める生命の危機、永久的な後遺症の発生が予想される重篤な有害事象が生じた際、試験責任医師はその事象

の治療関連行為との関連性の有無の判定に関わらず、できるだけ速やかに九州大学病院院長ならびに九州大学病院臨床試験倫理審査委員会委員長に報告する。また患者ならびに家族に対しても事態の説明を行う。

10.2.　対応方法

重篤な有害事象が生じた場合、主治医は直ちに試験責任医師に連絡を取り、試験責任医師は病院長に連絡する。

10.3.　研究の中止基準

1) ICH ガイドラインに定める重篤な有害事象のうち、NCI-CTC 基準の Grade３、４、ただし血液学的な有害事象に関しては Grade４を認めた場合や予期しない有害事象が認められた場合
2) 患者・家族が臨床研究の中止を希望された場合
3) 試験分担医師が臨床研究の継続が困難と判断した場合

上記基準の内、Grade３、４の有害事象や予測しない有害事象が認められた場合は、ただちに研究中止の有無を問う報告書を九州大学病院先進医療適応評価委員会に提出し、当該患者に対する研究継続に関する評価を受ける。研究の再開は、九州大学病院先進医療適応評価委員会により研究継続可能と判定された後に再開する。但し、当研究において投与薬剤による死亡例を認めた場合は、さらに九州大学病院臨床試験倫理審査委員会による研究継続の審査を受ける。

11.　研究経費

委任経理金ならびにトランスレーショナルリサーチ拠点事業関連の研究費を研究経費に充てる。

12.特許の帰属

RNF43 ペプチドに関する特許は東京大学医科学研究所ゲノムセンター教授中村祐輔ならび先端医療研究センター外科・臓器細胞工学分野教授田原秀晃に帰属する。本ペプチドを用いた活性化リンパ球ならびに樹状細胞療法自体の特許申請に関しては主に九州大学病院に帰属する。

13． 倫理面の配慮

13.1． 被験者に理解を求め同意を得る方法

研究責任医師または研究分担医師は研究実施に先立ち被験者本人に研究内容、被験者の権利などを十分に説明し、被験者が内容を理解したことを確認した後、本人の署名入りのインフォームドコンセントの文書を得る。記入・捺印、署名された写しを被験者に渡し、インフォームドコンセントの原本を保管する。その際説明を受ける者の人権を最優先し、自発的に同意が行われるように十分配慮する。研究者の連絡先を書いた文書を調査対象者に渡す。

13.2． 本法の不利益性、危険性

シクロホスファミドの投与による副作用として骨髄抑制、出血性膀胱炎、心筋障害等があげられるが、従来の化学療法の投与量の範囲内でおこり、今回の投与量は少ないため、重度の副作用発現の可能性は低いと考えられる。また IL-2 は副作用としては発熱が最も多いが、非ステロイド系の消炎剤投与により十分な対処が可能である。その他の副作用として浮腫等の水分貯留、うっ血性心不全、精神症状、肝、腎機能障害、自己免疫反応などの報告があるが、今回用いる投与量は通常投与量の半分量であり、注意深い観察を行いながら十分投与可能と考えられる。樹状細胞療法は注射部位や近傍のリンパ節に中等度以下の局所炎症反応を引き起こしたとの報告があるが、特に治療は必要ではなく、抗腫瘍免疫誘導効果に伴うものと考えられている。腫瘍浸潤リンパ球療法にてぶどう膜炎が起こったとの報告があるが、ステロイド点眼により軽快し、重篤な副作用は発生していない。むろん予期できぬアレルギー、ショック等の重大事象発生の可能性は否定できないため、十分な観察を行い、必要に応じて適切な治療を行えるように最大限の注意を払う。

13.3． ヒト由来試料提供者への予測される不利益とそれに対する配慮

HLA-A locus ならびに RNF43 遺伝子発現の解析により提供者が受ける不利益としては、遺伝子解析結果が外部に漏れた場合、社会における不当な差別などにつながる可能性が考えられる。しかし、固形腫瘍は単一の遺伝子の変異によって起こる疾患ではないため、遺伝子解析結果、患者およびその家族の疾病予防、診断、治療方針に直ちに影響することはないと予測される。しかし、万が一の漏洩による不利益を防ぐため、個人情報は研究実施担当者には匿名化され、

研究成果を公表する際には個人が特定される形では公表しないこととする。

13.4.　ヒト由来試料の保存予定の有無、保存が予定されている場合はその必要性と保存期間と方法、廃棄する場合は廃棄の方法

将来、リンパ球ならびに樹状細胞表面マーカーならびに機能解析等の免疫学的な解析、あるいはその他の遺伝子解析の試料として用いる可能性があるため、保存を予定している。ただし、文書にて同意を得たもののみを保存する。保存期間は使い切るまでとする。保存方法は、試料を符号化して匿名化のうえ管理するが、予後などとの関連を検討する可能性もあるため、個人識別情報管理者においては連結可能匿名化の状態で行う。廃棄については添付ナンバーなどを消去し、医療廃棄物として廃棄する。

13.5.　個人情報の保護の方法

B 群既採取試料については個人情報に関し連結不可能匿名化の状態で利用する。九州大学病院先端分子・細胞治療科にて臨床研究に参加の為の HLA-A locus ならびに RNF43 遺伝子発現の解析者に供与される情報は、個人識別情報を除き供与される。その場合、連結可能匿名化とする。すなわち、患者の氏名、生年月日は除去され、年齢、がんの種類、採取部位に関する情報のみが提供される。

個人情報に関しては、個人識別情報管理者を置き、情報の管理には細心の注意をはらう。個人情報管理者として谷憲三朗教授（九州大学病院先端分子・細部治療科）に担当を依頼する。

なお本解析結果はそれ以降の本臨床研究に患者が参加する為に必須な情報であるため、個人識別情報管理者より本臨床研究責任者へ速やかに文書にて連絡される。

保有する個人情報については、原則として開示等の求めに応じる。

13.6.　研究資金および利益の相反

本研究の計画・実施・報告において、研究の結果および結果の解釈に影響を及ぼすような「起こりえる利益の相反」は存在しない。および研究の実施が被験者の権利・利益を損ねることがないことを確認する。

１４． 連絡先

九州大学病院　先端分子・細胞治療科

試験分担医師：土方　康基　　電話　０９２－６４２－５９９６

（診療・細胞調製の責任者）

試験責任医師：谷　憲三朗　　電話　０９２－６４２－６４３４

（研究計画ならびにその実施全般における総括責任者）

文献

1) がんの統計'03　財団法人がん研究振興財団

2) Yagyu R, Furukawa Y, Lin Y, Shimokawa T, Yamamura T, Nakamura Y. A novel oncoprotein RNF43 functions in an autocrine manner in colorectal cancer. Int. J. Onco. 2004; 25: 1343-48.

3) Uchida N, Tsunoda T, Wada S, Furukawa Y, Nakamura Y, Thahara H. Ring finger protein (RNF) 43 as a New Target for Cancer Immunotherapy. Clin Cancer Res.2004; 10: 8577-86

4) Wang H. Y, Lee D. A, Peng G, Guo Z, Li y, Kiniwa Y, Shevach E., M, Wang R. Tumor-specific human CD4+ regulatory T cells and their ligands: implications for immunotherapy. Immunity. 2004; 20: 107-18.

5) Ghiringhelli F, Larmonier N, Schmitt E, Parcellier A, Cathelin D, Garrido C, Chauffert B, Solary E, Bonnotte B, and Martin F. CD4+CD25+ regulatory T cells suppress tumor immunity but are sensitive to cyclophosphamide which allows immunotherapy of established tumors to be curative. Eur J Immunol. 2004; 34: 336-44

6) Robbins P. F, Dudley M. E, Wunderlich J, El-Gamil M, Li Y. F, Zhou J, Huang J, Powell D. J, Rosenberg S. A. Cutting Edge: Persistence of Transferred lymphocyte Clonotypes Correlates with Cancer Regression in Patients Receiving Cell Transfer Therapy. J. Immunology.2004; 173: 7125-30

7) Kaech S.M, Wherry J, Ahmed R. Effector and Memory T-cell differentiation: Implications for vaccine development. Nature Rev. Immunology. 2002; 2:

251-62.

8) Schluns K.S and Lefrancois L. Cytokine control of memory T-cell development and survival. Nature Rev. Immunology. 2003; 3: 269-79.

9) Surh,C.D.and Sprent,J. Homeostasis of naïve and memory T cells. Immunity. 2008; 9: 848-62.

10) Lou Y, Wang G, Lisee G, Kim J. G, Finkelstein E.S, Feng C, Restifo P. N, Hwu P. Dendritic Cells Strongly Boost the Antitumor Activity of Adoptively Transferred T Cells In vivo. Caner Res. 2004; 64: 6783-90.

11) Tani K, Azuma M, Nakazaki Y, Oyaizu N, Hase H, Ohata J, Takahashi K, OiwaMonna M, Hanazawa K, Wakumoto Y, Kawai K, Noguchi M, Soda Y, Kunisaki R, Watari K, Takahashi S, Machida U, Satoh N, Tojo A, Maekawa T, Eriguchi M, Tomikawa S, Tahara H, Inoue Y, Yoshikawa H, Yamada Y, Iwamoto A, Hamada H, Yamashita N, Okumura K, Kakizoe T, Akaza H, Fujime M, Clift S, Ando D, Mulligan R, Asano S. Phase I study of autologous tumor vaccines transduced with the GM-CSF gene in four patients with stage IV renal cell cancer in Japan: clinical and immunological findings. Mol Ther. 2004; 10: 799-816.

12) M.E. Christine Lutsiak, Roshanak T, Semnani, Roberto De Pascalis, Syed V. S. Kashmini, Jeffrey Schiom, Helen Sabvevari. Inhibition of CD4+25+ T regulatory cell function implicated in enhanced immune response by low-dose cyclophosphamide. Blood. 2005; 105: 2862-68.

13) Ghiringhelli F, Larmonier N, Schmitt E, Parcellier A, Cathelin D, Garrido C, Chauffert B, Solary E, Bonnotte B, Martin F. CD4+CD25+ regulatory T cells suppress tumor immunity but are sensitive to cyclophosphamide which allows immunotherapy of established tumors to be curative. Eur J Immunol. 2004; 34: 336-44.

14) Konno S, Asano K, Gonokami Y, Kurokawa M, Kawazu K, Adachi M. Effect of IPD-1151T(Suplatast tosilate)on airway hyperresponsiveness in mice. Arerugi. 1995; 44: 556-61

15) C.D. Jurincic-Winklera, K.A. Metzb, J. Beuthc, K.F. Klippela. Keyhole Limpet Hemocyanin for Carcinoma in situ of the Bladder: A Long-Term Follow-Up Study Eur Urol. 2000;37:45-49

16) Yukio Iwashita, Kouichirou Tahara, Shigeru Goto, Atsushi Sasaki, Seiichiro Kai, Masataka Seike, Chao-Long Chen, Katsunori Kawano, Seigo Kitano. A phase I study of autologous dendritic cell-based immunotherapy for patients with unresectable primary liver cancer. Cancer Immunol Immunother. 2003;52:155-161
17) Lorenz Höltl, Reinhold Ramoner, Claudia Zelle-Rieser Hubert Gander, Thomas Putz, Christine Papesh, Walter Nussbaumer, Claudia Falkensammer, Georg Bartsch, Martin Thurnher. Allogeneic dendritic cell vaccination against metastatic renal cell carcinoma with or without cyclophosphamide. Cancer Immunol Immunother. 2005; 54: 663-670
18) Dudley M. E, Wunderlich J. R, Robbins P. F, Yang J. C, Hwu P, Schwartzentruber D. J, Topalian S. L, Sherry R, Restifo N. P, Hubicki A. M, Robinson M. R, Raffeld M, Duray P, Seipp C. A, Rogers-Freezer L, Morton K E, Mavroukakis S. A, White D.E, Rosenberg S. A, Cancer Regression and Autoimmunity in Patients After Clonal Repopulation with Antitumor Lymphocytes. Science. 2002; 298 (25): 850-4.
19) Cranmer LD, Trevor KT, Hersh EM. Clinical applications of dendritic cell vaccination in the treatment of cancer. Cancer Immunol immunother. 2004; 53: 275-306.
20) Chakraverty R, Peggs K, Chopra R, Milligan DW, Kottaridis PD, Verfuerth S, Geary J, Thuraisundaram D, Branson K, Chakrabarti S, Mahendra P, Craddock C, Parker A, Hunter A, Hale G, Waldmann H, Williams CD, Yong K, Linch DC, Goldstone AH, Mackinnon S. Limmiting transplantation-related mortality following unrelated donor stem cell transplantation by using a nonmyeloablative Conditioning regimen. Blood. 2002; 99: 1071-8.
21) Dudley M E, Wunderlich J R, Sherry R M, Topalian S L, Restifo N P, Royal R E, Kammula d, White D E, Mavroukakis S A, Rogers L J, et al, Adoptive Cell Transfer Therapy Following Non-Myeloablative but Lymphodepleting Chemotherapy for the Treatment of Patients With Refractory Metastatic Melanoma. J. Clin. Oncol 2005; 23: 2346-57.
