# Supplementary material for: A phase I clinical trial of RNF43 peptide-related immune cell therapy combined with low-dose cyclophosphamide in patients with advanced solid tumors
Source: PLoS One. 2018 Jan 2;13(1):e0187878. doi: 10.1371/journal.pone.0187878 (PMC5749706; doi:10.1371/journal.pone.0187878)
Supplement: S3 File — (PDF) [file pone.0187878.s003.pdf]

Protocol

# A phase 1 study of adoptive immunotherapy using RNF43 peptide-pulsed dendritic cells and RNF43 peptide-activated lymphocytes in patients with advanced solid tumors

Version 3.0

Principal investigator

Kyushu University Hospital

Professor, Department of Advanced Molecular and Cell Therapy

Kenzaburo Tani

## 1. Title

A phase 1 study of adoptive immunotherapy using RNF43 peptide-pulsed dendritic cells and RNF43 peptide-activated lymphocytes in patients with advanced solid tumors

## 2. Objectives

This study aimed to specifically activate lymphocytes by presenting ring finger protein (RNF) 43 peptide, a tumor-specific antigen peptide, to dendritic cells (DCs) in vitro and in co-culture with peripheral blood lymphocytes. Thereafter, these activated lymphocytes and RNF43 peptide-pulsed dendritic cells were administered to patients for the evaluation of safety and the antitumor immunity induction effect in terms of their applicability to in vitro studies and their clinical efficacy.

## 3. Background

Treatment outcomes for cancer have improved year after year owing to the recent advent of multidisciplinary therapy. However, for patients with intractable disease or with recurrent disease, existing general treatments including operative therapy, chemotherapy, and radiation therapy are not effective, and the only option provided to these patients is palliative therapy-centered supportive care [1]. Therefore, a novel treatment method is urgently required for those patients to who cannot expect to achieve complete remission. A potential candidate for such a method is immunotherapy.

*RNF43* has been identified as a novel gene highly expressed in cases of colorectal cancer, as identified through microarray profiling of 23,040 genes. This gene is located on the short arm of chromosome 22, and the resultant protein is composed of 783 amino acids. RNF43 localizes in the nucleus and cytoplasm of tumor cells and is partially characterized as a secretory protein. RNF43 is not expressed in normal cells, but it is expressed in more than 85% cases of colorectal cancer [2]. DNA microarray profiling has revealed high expression of RNF43 in cases of bladder cancer and non-small cell lung cancer (Furukawa et al, private communication). In addition, this protein may be functionally related to tumor growth [2]. Tahara et al. successfully induced cytotoxic T lymphocyte (CTL) clones with strong cytotoxic activity in targeted peptide-pulsed cells by stimulating CD8-positive T cells using an RNF43-derived peptide binding to HLA-A*0201 (ALWPWLLMA) and a peptide binding to HLA-A*2402

(NSQPVWLCL). These CTL clones endogenously expressed RNF43 and had strong cytotoxic activity specific to colorectal cancer cell lines with HLA-A*0201 or HLA-A*2402. Moreover, specificity of the cytotoxic activity was confirmed by the cold target inhibition assay and blocking assay [3]. Based on the above findings, it was suggested that RNF43 has notable immunogenicity and can be applied to cancer immunotherapy by inducing the CTLs that recognize and impair RNF43.

Among peptide-based antitumor immunotherapies, only a few therapies have reported a stable outcome, possibly because regulatory T cells (Tregs) deactivate the activated immune cells administered at tumor sites [4]. However, it was recently reported that administration of cyclophosphamide (CPM) resulted in the depletion of these Tregs, which may enhance the effects of immunotherapy [5]. Depletion of administered activated immune cell clones to a living body was indicated as another reason for the poor responses of immunotherapy [6], and another study reported that activated immune cell clones were maintained in the body for a long period of time in patients who responded to antitumor immunotherapy [7]. To acquire CTLs that can be maintained in the body for a long period of time and show strong reactivity to tumor antigens, the induction of effector cells and memory cells is necessary. On stimulation of naive T cells by antigen-presenting cells (in the initiation phase), the antigen-specific T cell clones become effector cells after growth (clonal expansion); however, after this, most activated effector T cells undergo apoptosis (contraction phase) [8]. Some T cells with high-affinity T cell receptors against antigens survive as $CD44^{high}$ memory T cells that lead to a stronger acquired response to subsequent instances of antigen stimulation (maintenance phase). The necessity of interleukin (IL)-7 and IL-15 for maintenance of those effector cells and memory cells has been reported [9]. Another study also reported that administration of activated lymphocytes followed by immunization with DCs, resulted in the maintenance of these cells in the body for a longer period time than that after DC therapy alone, thus, enabling the induction of stronger antitumor immunity in a mouse model [10].

## 4. Past achievements and expectations for the future

We have completed the study titled “A clinical study of immunogene therapy for IV renal cell cancer”, which is the first clinical study of gene therapy for malignant tumors

conducted in Japan, at the Institute of Medical Science Hospital, The University of Tokyo [11]. Our laboratory possesses the following requisite technologies to conduct this clinical study; thus, we believe that we can fully accomplish this clinical study. Furthermore, the Institute of Medical Science at the University of Tokyo conducted a tumor-specific immunization therapy (Phase I clinical study) using a novel cancer-related antigen RNF43-derived HLA-A*2402-restricted epitope peptide for advanced colorectal cancer and confirmed the safety of this peptide. In the current Phase I clinical study of potent adoptive immunotherapy for patients with advanced solid tumors, Tregs were depleted using CPM on the administration of lymphocytes activated in vitro using RNF43 peptide-pulsed dendritic cells, and RNF43 peptide-pulsed DCs and IL-2 were administered for maintenance and activation of the activated lymphocytes. This is the first clinical study adopting this method, through which we believe that we can expect a greater antitumor effect.

**4.1. Isolation of DCs**

Delete confidential information

**4.2. Induction of activated T-lymphocytes**

We have conducted clinical studies of cancer immunotherapies using pulsed-mature DCs with custom-made multi-peptides, and we are proficient in the in vitro antitumor immunological analysis conducted herein. For instance, we have examined the induction of activated lymphocytes via co-culture of dendritic cells (oncolysate phagocytes) and peripheral blood T lymphocytes and by examining their cytotoxic activity, and we have examined the surface markers of induced cells via co-culture of pulsed mature dendritic cells and PBMCs. Based on the results of these investigations, we have confirmed that 3 or more antigen presentations are required for valid induction of activated lymphocytes and CD8-positive cells were significantly increased after co-culture of peptide-pulsed mature DCs and PBMCs.

**4.3. Enhanced antitumor effects after depletion of regulatory T cells using CPM**

We have gained a great deal of experience in the safe administration of CPM for general treatment of hematologic malignancy. Recently, CPM administration has been reported to specifically deplete Tregs, resulting in the suppression of tumor immunity and

possibly enhancing the effect of immune cell therapy. Furthermore, this effect can be exerted by administration of a low-dose of CPM [12], and it has been documented that CPM administration 4-7 days before the administration of activating lymphocytes is effective [13]. Therefore, low-dose CPM can be used for the purpose of Treg depletion.

## 5. Safety

### 5.1. Contamination by pyrogenous substances/microorganisms

All reagents to be used are clinical-grade or equivalent. A series of incubation processes are implemented in the cell processing room in Kyushu University Hospital. Immunostimulators such as granulocyte macrophage colony-stimulating factor (GM-CSF), IL-4, TNF-α, KLH, and OK432 are incubated to induce dendritic cells, while IL-2, IL-7, IL-12, and IL-15 are incubated to induce activated lymphocytes. Systemic IL-2 administration is performed to maintain infused activated lymphocytes in the body for a long period of time. It is an approved drug in Japan that has been administered to many patients. Pyrexia is the most frequently observed adverse reaction but it can be treated efficiently by non-steroidal anti-inflammatory drug administration. While edema, congestive heart failure, mental symptoms, and liver/renal dysfunction have been reported as the other adverse autoimmune reactions, IL-2 administration is feasible with careful observation because the dose to be used in this study is half the usual dose. OK432 has also been approved for treatment of patients with gastrointestinal cancer, lung cancer with cancerous pleural effusion/ascites, cancers of the head and neck (maxillary cancer, laryngeal cancer, pharyngeal cancer, and tongue cancer) resistant to other drugs, and thyroid cancer, and has been administered to many patients. Hypersensitivity, shock, interstitial pneumonia, and acute renal dysfunction have been reported as adverse reactions of this treatment. GM-CSF is approved in countries such as the US as a drug for treatment of acute myeloid leukemia aimed at mobilizing allogeneic and autologous peripheral blood stem cells for monotherapy and increasing leukocytes after chemotherapy, and has been administered to a lot of patients. However, edema, respiratory disorder, supraventricular arrhythmia, and liver/renal dysfunction have been reported as adverse reactions (based on Ethical Drug Package Insert). IL-4, IL-7, IL-12, IL-15, TNF-α, and KLH are not approved for use as drugs in Japan and other countries; thus, the research reagent (GMP Grade) will be used in vitro.

KLH may induce allergic reactions because it is used in the preparation of a mouse model of allergy-driven airway contraction [14]; however, no Grade 3 or higher adverse event has been reported, although redness at the inoculation site, pain, and induration have been observed during treatment of malignant myeloma and bladder cancer [15]. No adverse reaction has been reported in studies in which KLH was used for induction of DCs in the treatment of patients with primary liver cancer and renal cancer [16,17]. These drugs and cultured cells will be administered intravenously or subcutaneously after 3 washes. That is, when lymphocytes are washed with 500 ml of physiological saline, no more than 2 ml of cell pellet is obtained; thus, the contamination levels will be reduced to 1/250 or lower after washing once and $1/10\times10^7$ or lower after washing 3 times. When DCs are washed with 10 ml of physiological saline, no more than 100 μl of the cell pellet is obtained, and the contamination level will be reduced to 1/100 or lower after washing once and $1/10^6$ or lower after washing 3 times. Therefore, any drug or cytokine will be present at an almost negligible level in the patient blood.

**5.2. Possibility of autoimmune response induction**

While it is indicated that autologous immunological disease may be induced by the activation of self-reactive T cells by autologous antigens presented on DCs, it is unlikely to occur because self-reactive T cells are originally deleted through negative selection in the thymus gland. Although there have been reports on the occurrence of vitiligo vulgaris and uveitis due to tumor-infiltrating lymphocytes (TIL) therapy for melanoma after non-myeloablative conditioning, vitiligo vulgaris is not a clinically serious adverse reaction, and uveitis was found to be improved after treatment with corticosteroid eye drops and visual impairment was not developed [18]. These adverse events are considered specific to immunotherapy for melanoma. Although there is a risk of production of antibodies to serum preparation and cytokines used for incubation, it was determined to be negligible based on the severity of the primary disease, and no induction of autologous immunological disease has been reported in studies involving in vivo administration to humans.

Moreover, we have not experienced any adverse reaction in the administration of various tumor antigen pulsed-dendritic cells in our clinical studies of cancer immunotherapy using pulsed-mature DCs with tailor made multi-peptides. However, to

ensure patient safety, the patients will be hospitalized and observed for administration of cells and IL-2, and will be asked to visit the hospital regularly after the completion of administration, for monitoring of the occurrence of adverse reactions, to ensure that immediate action is taken in the case of adverse reactions.

### 5.3. Cell count

In this clinical study, a DC count of $1\times10^7$ cells will be administered to the patients each time. In previous studies, a dose of $10^8$ DCs were safely administered subcutaneously [19], and no particular adverse event has been observed in the administration of $1\times10^7$ cells in our clinical studies of cancer immunotherapies conducted using pulsed-mature DCs with tailor made multi-peptides. In this study, the same DC count will be used, to ensure safe administration.

RNF43, to be used as an antigen in this therapy, is unlikely to be expressed in normal cells, and injury of normal tissues due to activated lymphocytes is unlikely to occur. However, activated lymphocytes and DCs have not been administered simultaneously.

In a previous study of allogeneic donor lymphocyte infusion therapy for recurrent chronic myeloid leukemia after allogeneic bone marrow transplantation, Grade 2 and 3 graft versus host disease (GVHD) has been observed after the injection of $6\times10^7$ $CD3^+$cells (60 kg) and $6\times10^8$ $CD3^+$cells (60 kg), respectively [20]. On the other hand, $1\times10^9$ lymphocytes can be generally safely administered without any serious adverse event in autologous activated lymphocyte therapy. With reference to previous reports, a 2-step dose-escalation procedure ($5\times10^7$ cells and $2\times10^8$ cells) will be adopted in this clinical study to administer cells, ensuring patient safety.

### 5.4. Adverse reaction due to premedication with CPM

Adverse reactions caused by the administration of CPM generally include bone-marrow suppression and hemorrhagic cystitis; however, a dose of CPM (300 mg/m$^2$) lower than that used in conventional chemotherapy (about 750 mg/m$^2$) will be administered in this clinical study; therefore, severe adverse reactions are unlikely to occur.

### 5.5. Apheresis

Adverse reactions caused by this treatment include symptoms of vasovagal reflex and

transient dehydration, such as numbness in limbs, dizziness, nausea, vomiting, as well as general malaise. To resolve these adverse reactions, a system will be established, in which physicians who are familiar with performing apheresis would take immediate measures, along with ECG monitoring. As a decrease in platelet count is occasionally observed after the collection of PBMCs, blood platelet count will be checked, as required, to confirm recovery to the value before collection.

### 5.6. Safety of RNF43 peptide

A tumor-specific vaccination therapy (Phase I clinical study) using HLA-restricted RNF43-derived epitope peptide in 8 patients with HLA-A*2402-positive advanced colorectal cancer was conducted at the Department of Surgery, Institute of Medical Science, University of Tokyo (principal investigator: Hideaki Tahara, Kanamoto Akira). Only redness induration at the inoculation site was observed as a related adverse event, and no Grade 3 or higher adverse event was observed. Thus, the safety of this clinical study, a primary outcome of this Phase I clinical study, was confirmed.

### 5.7. Safety of dendritic cells

We have been conducting a clinical study of cancer immunotherapy using pulsed-mature DCs with tailor made multi-peptides at our department, and we have immunized 5 patients. With regards to adverse events, only redness induration at the inoculation site was observed, and no Grade 3 or higher adverse event was observed.

## 6. Criteria for cell preparation

The culture of DCs and activated lymphocytes will be checked for pathogenicity and immune cell surface markers, and those cells that are proven to be non-pathogenic will be administered to the patients. The information on immune cell surface markers and clinical data (immune response test, objective tumor response, adverse event) will be compared and examined.

## 7. Design

[Overview]

Among patients with advanced solid tumors refractory to existing treatments such as

anti-cancer drug/radiation who are not expected to achieve complete remission by surgery and whose prognosis is considered extremely poor, patients with confirmed HLA-A*0201 or HLA-A*2402-positive status and RNF43 expression in tumor cells/tissues are included in this study. In principle, a sufficient explanation about the disease name and stage, the study protocol schedule, alternative modes of treatment, side-effects, and risk-benefit ratios is given to the patients, and written informed consent is obtained from patients after the patients fully understand that they cannot always benefit from this treatment.

[**Sample size**]

The total number of evaluable patients who complete the immunization of activated lymphocytes and the fourth-week evaluation after 3 DC immunizations will be 10.

[**Cost**]

Patients will not be charged for the cost of cell preparation and administration or the costs of treatment and hospitalization during the clinical study period i.e. the period of cell administration. Conventional payment methods (e.g. health insurance) will be adopted for general tests (imaging such as an X-ray examination, computed tomography (CT) scan, and blood test) or surgical treatment, for determination of symptoms of the primary disease performed outside the clinical study period, and associated hospitalization costs.

**7.1. Dose cycle and observation schedule**

Chemotherapy (CPM) will be given on Day 1, followed by the administration of the cell preparation 3 times over 2 weeks starting on Day 6. On Day 6, the activated lymphocytes and DCs will be administered and IL-2 will be inoculated for 3 consecutive days after the first DC immunization. Thereafter, the second and the third immunization of DCs will be conducted on Day 13 and Day 20, respectively, and IL-2 will be inoculated for 3 consecutive days after each DC immunization in the same manner.

The cell count to be administered in this clinical study is as follows: activated lymphocyte: $5\times10^7$ cells for level 1 and $2\times10^8$ cells for level 2, dendritic cells: $1\times10^7$ cells for both level 1 and 2. Activated lymphocytes will be suspended in 100 ml of 2%

physiological saline containing serum and intravenously infused over a period of approximately 1 hour. DCs will be suspended in 1.0 ml of physiological saline and subcutaneously (or intracutaneously) administered. IL-2 [$35\times10^4$ IU/350 μl distilled water for injection] will be subcutaneously administered each time. CPM (300 mg/$m^2$) will be added to 500 ml of physiological saline and intravenously infused (over a period of approximately 2–3 hours).

### 7.2. Cell preparation schedule

Delete confidential information

### 7.3. Eligibility

#### Patients

The eligibility criteria were as follows:

i. Patients with advanced solid tumors, previously treated with available standard therapies;
ii. Eastern Cooperative Oncology Group (ECOG) performance status of 0 to 1;
iii. HLA-A*24:02- or A*02:01-positive status;
iv. Polymerase chain reaction (PCR)-confirmed RNF43-antigen expression of tumor cells;
v. Age between 20 and 70 years;
vi. No prior therapy within 4 weeks of enrollment;

vii. Adequate hematology (Hemoglobin, > 8 g/dL; WBC, > 3000 cells/μl; Platelets, > $10^5$/μl), renal (creatinine, < 1.5 mg/dL) and hepatic function (total bilirubin < 2.0 mg/dL, aspartate aminotransferase < 99 U/L, alanine aminotransferase < 90 U/L);

viii. Presence of measurable tumor, to allow assessment of clinical response;

ix. Life expectancy of at least 3 months;

x. Negative for hepatitis B antigen, anti-hepatitis C antibody, anti-HIV antibody, anti-HTLV-1 antibody, and syphilis serodiagnosis;

xi. Written informed consent obtained at the time of enrollment.

The following exclusion criteria were applied:

i. Patients with severe pre-existing diseases;

ii. Presence of autoimmune disease, active infectious disease, cardiovascular disorders, respiratory disorders, renal dysfunction, immunodeficiency, and hematological disorders;

iii. Pregnant, lactating, or possibly pregnant women, or those willing to be pregnant, or willing male partner;

iv. Presence of brain metastases;

v. Patients who required systemic administration of steroid or immunosuppressive agents;

vi. Patients who were inappropriate for study entry, as judged by the attending physician.

**7.4. Consent procedure**

This has been explained in detail in the attached document titled "Phase I clinical study of potent adoptive immunotherapy for patients with advanced solid tumor using RNF43-pulsed DCs and activated lymphocytes." Written informed consent was obtained from all participants.

**7.5. Actual treatment**

**7.5.1. Selection of targeted patients with cancer**

The targeted population are patients who received pre-medication, have advanced solid tumors or solid tumors refractory to existing standard treatments, are HLA-A*0201 or HLA-A*2402-positive, and express RNF43 in tumor cells or tumor tissue.

**(1) Determination of HLA-A*0201 and HLA-A*2402 expression**

HLA-DNA typing was done at Wakunaga Pharmaceutical Co., Ltd. using WAKFlow HLA typing reagent (see attached manual) (Wakunaga Pharmaceutical Co., Ltd., Hiroshima).

**(2) Determination of RNF43 expression using real-time reverse transcription**

**polymerase chain reaction**

Tumor cells or tumor tissues sampled through biopsy (e.g. endoscopic biopsy, transdermal biopsy) are used to extract total RNA. cDNA is then synthesized from the total RNAs using an RNF43 gene-specific primer

FW: CCAGTGTGGTTGTGCCTGAC;

RV: CATGAAGGATCTTCTGTGACCT

Relative quantification of RNF43 is then performed by real-time PCR using SYBR Green I. For patients for whom new biopsies are not feasible, previously collected and stored formalin fixed-samples can be used for perform the same tests.

### 7.5.2. Evaluation of imaging diagnosis immediately before treatment

Immediately before cell preparation, targeted lesions are evaluated by diagnostic imaging (including CT) for conformity with the WHO-RECIST criteria.

### 7.5.3. Collection of peripheral blood mononuclear cells by apheresis or blood sampling

More than $1.5 \times 10^9$ of PBMCs are separated from peripheral blood, collected by the apheresis and blood sampling.

### 7.5.4. RNF43 peptide

GMP-grade peptides purchased from Multiple Peptide Systems (San Diego, CA) are used. They are stored in a freezer within in the cell processing room after being dissolved via a predetermined method followed by dispensing. These peptides are then stored in a freezer at a predetermined temperature (-80°C).

### 7.5.5. Preparation of patient's autologous peripheral blood mononuclear cell-derived dendritic cells

Adherent cells and non-adherent cells are separated from PBMCs using a plastic plate, and the dendritic cells (DCs) are produced from adherent cells. Produced DCs are pulsed with RNF43 peptide at the final concentration of 20 μg/ml and are cryopreserved until use or used for co-culture with PBMCs and subcutaneous (intracutaneous) injection.

#### 7.5.6. Preparation of RNF43 peptide-activated lymphocytes

Activated lymphocytes are induced by co-culturing non-adherent cells, isolated from PBMCs using a plastic plate, with RNF43 peptide-pulsed DCs. Antigen presentation in RNF43 peptide-pulsed DCs was performed thrice.

#### 7.5.7. Intravenous infusion of RNF43 peptide-activated lymphocytes

Activated lymphocytes are washed with a Japanese Pharmacopoeia physiological saline and intravenously infused to patients.

#### 7.5.8. Initial dose and dose increment design of RNF43 peptide specifically activated lymphocytes

A two-step dose-escalation study (discussed later) is conducted to determine the number of activated lymphocytes to be administered, as shown in the table below. Patients are allocated to the two dose levels in sequential order. The dose administered will never exceed the predetermined dose for any patient. This administration method will be discussed later in detail.

7.5.8. Table

| Level | RNF43 peptide specifically activated lymphocytes |
|---|---|
| Level 1 | $5x10^7$ |
| Level 2 | $2x10^8$ |

Activated lymphocytes will continue to be administered to all patients unless dose limiting toxicities (DLT) or Grade 3 or higher adverse events (National Cancer Institute-Common Terminology for adverse event version 4: NCI CTCAE ver.4) are observed. When administration is safely completed for 5 patients registered in level 1, they are then proceeded to level 2. If DLT is found in level 1 in 1 patient, another patient is added to level 1 to observe the reproducibility of DLT. If no DLT is observed in this patient, then new patients will proceed to level 2. If DLT is found in 2 patients, this study will be terminated. In level 2, 5 patients are also registered, and the study will be completed when administration is safely completed. Similar to the procedure for

level 1, in level 2, if DLT is found in 1 patient at the predetermined dose level, another patient will be treated to observe the reproducibility of DLT. If DLT is found in 2 patients, level 2 ($2x10^8$ cells) will be DLT and the study will be terminated. If DLT is found in 1 patient at level 2, the study will be completed when administration is safely completed in 5 patients. However, if there is any drop-out case at both levels, another patient will be allocated to each level.

### 7.5.9. Subcutaneous injection of RNF43 peptide-pulsed dendritic cells

RNF43 peptide-pulsed DCs are cryopreserved until use, thawed before subcutaneous injection, and washed with saline solution. RNF43 peptide-pulsed DCs ($1\times10^7$/time) are subcutaneously inoculated near the regional lymph node. The DCs are administered 3 times in total as follows: at injection of lymphocytes, activated 1 week after injection of lymphocytes, and at Week 2 after injection of activated lymphocytes.

### 7.5.10. Use of interleukin-2

Although interleukin-2 (IL-2) is generally used for angiosarcoma and renal cancer treatment in Japan, its usage in this clinical study is off-label. However, systemic administration of IL-2 in TIL therapy has previously been reported to activate injected lymphocytes and enhance antitumor effect [21]. IL-2 (proleukin®) is used for further *in vitro* activation of induced activated lymphocytes in this study. The preparation is dissolved in distilled water for injection at the Kyushu University Molecular and Cell Processing Center, and then dispensed and cryopreserved. It is thawed for use and subcutaneous inoculation. If eligible patients have concomitant symptoms (e.g. pleural effusion, ascites, intestinal obstruction, respiratory bleeding, or gastrointestinal bleeding), IL-2 use is carefully examined because its administration may worsened those symptoms; if necessary, IL-2 administration can be omitted.

### 7.5.11. Administration of CPM

A study reported that CPM deleted Treg and enhanced antitumor effect [317, thus, CPM will be used in this study for the same purpose.

## 7.6. Additional administration

If there is sufficient remaining cell count and the attending physician considers it appropriate, additional activated lymphocytes and dendritic cells can be injected into the patient with his consent. However, these instances are not included in the data of this clinical study and are regarded as reference data. When additional inoculation is performed, it is reported to the Research Ethics Committee and also explained to the patients to obtain consent.

## 8. Evaluation

### 8.1. Period

The evaluation period is set from the start date of CPM administration to 4 weeks after the completion of administration of the cell preparation.

### 8.2. Items

1) Evaluation of adverse events: National Cancer Institute-Common Terminology for adverse event version 4 (NCI CTCAE) (Attachment) is used as the basis of evaluation.
2) Antitumor effect: WHO-RECIST criteria is used as the basis of evaluation.
3) General blood test (peripheral blood test, biochemical examination), tumor marker (increased tumor-specific marker such as serum carcinoembryonic antigen (CEA) in the tumor in preliminary screening test), urinalysis, fecal occult blood
4) Evaluation of survival time: follow-up observations will be conducted for 3 years since completion of the clinical study evaluation period.
5) Evaluation of immune reaction:

(1) Delayed type hypersensitivity (DTH) reaction: changes in DTH reaction to RNF43 peptide before and after the clinical study are examined. Physiological saline and purified protein derivative are used as the negative and positive controls, respectively.

(2) Cell surface marker search: various surface markers for DCs and lymphocytes induced in vitro are searched, and maturity of DCs and lymphocyte phenotypes such as naïve, memory, and effector are evaluated.

(3) Intracellular cytokine measurement: cell activation by antigen reaction is examined by measuring whether intracellular cytokines (INFγ, IL-2, and TNF-α)

are induced by RNF43 peptide stimulation in patient's PBMCs before and after administration of cell preparation.

(4) Cytokine measurement: patient's serum cytokines (e.g. IL-1β, IL-2, IL-4, IL-5, IL-6, IL-8, IL-10, TNF-α, INF-γ, and IL-12p70) are measured to analyze the changes that occurred before and after administration of cell preparation.

(5) Evaluation of CTL activation: peripheral blood lymphocytes before and after administration of cell preparation are mixed and cultured with peptide to evaluate the changes in RNF43 peptide-specific CD107a/b positive T cells.

(6) Treg measurement: CPM-induced depletion of Tregs ($CD4^+$, $CD25^+$ and $Foxp3^+$) in patient's peripheral lymphocytes are evaluated before and after administration of CPM.

## 9. Endpoint

1) Primary endpoint:
Examination of safety and adverse events of this study

2) Secondary endpoint:
Examination of overall survival, antitumor effect (clinical efficacy) and Immunological evaluation.

## 10. Measures undertaken to deal with adverse events

### 10.1. Record of adverse events

Attending physician shall record all adverse events specified in the International Conference on Harmonization (ICH) guideline, regardless of a causal relation with this adoptive immunotherapy. The presence or absence and severity of adverse events are determined according to the NCI CTCAE ver.4. Moreover, causal relation is also assumed at the same time. The principal investigator shall report to the director of Kyushu University Hospital and the chairman of Kyushu University Hospital clinical study ethic committee as quickly as possible in case of a Grade 3 or higher abnormality in non-hematological test, or a Grade 4 abnormality in hematological test (in conformity with the clinical examination item of the NCI-CTCAE ver. 4), death, any life-threatening risk specified in the ICH E2A and E2D guideline, or serious adverse events that may result in permanent damage during the study and within 4 weeks since

administration of the final cell preparation, regardless of the relation of those events with the treatment. These events are explained to patients and their family.

### 10.2. Measures

In case of serious adverse event, the attending physician shall immediately contact the principal investigator, and the principal investigator shall contact the hospital director.

### 10.3. Conditions for halting the study

1) When Grade 3/4 adverse events (Grade 4 for hematological adverse events) (NCI-CTC criteria) and unexpected adverse events that are listed among the serious adverse events specified in the ICH guideline are observed.
2) When patients/their family member(s) desire their withdrawal from the clinical study.
3) When the sub-investigator determines the continuation of the clinical study is difficult.

Among the above criteria, when Grade 3/4 adverse events or unexpected adverse events are observed, a report should be immediately submitted to the Research Ethics Committee of Kyushu University Hospital to determine whether the clinical study can be continued. The study will be resumed after the Research Ethics Committee of Kyushu University Hospital permits it. However, if death occurs related to the drug administration conducted in the study, the Kyushu University Hospital Clinical Study Ethic Committee will further evaluate the continuation of the clinical study.

## 11. Research cost

Research funds from the trust accounts and the translational research core center project will be allocated to this research.

## 12. Attribution of patent rights

The patent rights of RNF43 peptide are attributable to Yusuke Nakamura, a professor of the Human Genome Center, Institute of Medical Science, the University of Tokyo and Tahara Hideaki, a professor of the Department of Surgery and Bioengineering, Advanced Clinical Research Center, Institute of Medical Science, the University of Tokyo. The right of patent filing for activated lymphocyte and dendritic cell therapy using this peptide is mainly attributable to the Kyushu University Hospital.

## 13. Ethical considerations

### 13.1. Explanation of the study to patients and obtainment of their consent

Prior to conducting the study, the principle investigator or sub-investigator offered a full explanation of the details of the study, the subject's rights, etc. to the subject, and obtained informed consent with the subject's signature after confirming that the subject had understood the implications. A copy of the informed consent with the name and signature was given to the subject, and the original copy was stored. The principle investigator or sub-investigator was careful to give first priority to the subject's rights and let the subject voluntarily give his/her consent. The subject was provided with a document containing the contact information of the researchers.

### 13.2. Disadvantages and risks of this therapy

While adverse reactions caused by CPM include bone-marrow suppression, hemorrhagic cystitis, and myocardial dysfunction, these reaction were caused when CPM was used at a dose similar to that for conventional chemotherapy. The dose used in this study is lower than that; hence, severe adverse reaction is unlikely to occur. Pyrexia is most often reported as an adverse reaction to IL-2; however, this can be appropriately treated with non-steroidal anti-inflammatory drugs. While water retention (edema), congestive heart failure, mental symptoms, liver dysfunction, renal dysfunction, and autoimmune response have been reported as other adverse reactions, the dose used in this study was about half of the usual dose. Thus, this dose could be safely administered under careful observation. Therapy using DCs has been reported to cause mild or intermediate local inflammatory reaction at the injection site or near the lymph node; however, no particular treatment was required and this may be associated with the antitumor immunity-induced effect. While the development of uveitis after tumor invasion lymphocyte therapy was reported, the symptoms were improved by applying corticosteroid eye drops, and no serious adverse reaction was observed. Of course, the possibility of serious events, including unexpected allergy and shock cannot be denied; therefore, we adequately observed and took great care so that appropriate treatment could be given, if needed.

### 13.3. Expected disadvantages to the sample donor and other considerations

A disadvantage of the HLA-A locus and RNF43 gene expression analysis for the donor is that the leaking of genetic analysis results to outsiders may lead to unjust discrimination in society. The genetic analysis results will notimmediately result in disease prevention, diagnosis, and treatment policy for the patients and their family as solid tumors are usually not caused by the mutation of a single gene. Moreover, personal information was anonymized to avoid disadvantageous events due to the unexpected leakage of information, and the published study results will not contain any personally identifiable information.

### 13.4. Storage of human-derived samples, its necessity and methods, and method in case of disposal

Residual samples were stored because they may be used for analysis of surface markers of lymphocytes and DCs, immunological analysis, such as functional analysis, or other genetic analyses in the future. Nevertheless, samples were only stored with written consent. They will be stored until all samples are used. They were handled after coding and anonymization; however, a personal identification information manager adopted linkable anonymization to manage the samples because our data may be examined in relation to the prognosis. When they are disposed, the attached number, etc. will be erased and they will be disposed as medical waste.

### 13.5. Protection method for personal information

For samples collected from Group B based on the guideline in Ministry of Health, Labour and Welfare, personal information was unlinked and anonymized before use. The analyzer of the HLA-A locus and RNF43 gene expression at Kyushu University Hospital Department of Advanced Molecular and Cell Therapy was provided the patient’s information, excluding any personally identifiable information. That is, only information on age, cancer type, and collection site was provided and the name and birth date of patients were removed.

A manager of personal identification information—Kenzaburo Tani (Professor of the Department of Advanced Molecular and Cell Therapy of Kyushu University Hospital) —was appointed, and he paid close attention to the information management.

As these analysis results are essential information for subsequent patients to participate in this clinical study, the personal identification information manager immediately reported the results to the director of this clinical study in writing.
We will respond to any requests for disclosure of the acquired personal information.

### 13.6. Research fund and conflict of interest

There is no "conflict of interest" that may affect the design, implementation, and reporting of this study, the study results, and the interpretation of these results. We also verify that this study did not violate the rights and health of the patient.

## 14. Contact

Kyushu University Hospital

Sub-investigator: Yasuki Hijikata, Phone: 092-642-5996

(responsible person for medical practice/cell preparation)

Principal investigator: Kenzaburo Tani, Phone: 092-642-6434

(Director of study design and its implementation)

## references

1) Cancer statistics in Japan 2003. Foundation for Promotion of Cancer Research
2) Yagyu R, Furukawa Y, Lin Y, Shimokawa T, Yamamura T, Nakamura Y. A novel oncoprotein RNF43 functions in an autocrine manner in colorectal cancer. Int. J. Onco. 2004; 25: 1343-48.
3) Uchida N, Tsunoda T, Wada S, Furukawa Y, Nakamura Y, Thahara H. Ring finger protein (RNF) 43 as a New Target for Cancer Immunotherapy. Clin Cancer Res.2004; 10: 8577-86
4) Wang H. Y, Lee D. A, Peng G, Guo Z, Li y, Kiniwa Y, Shevach E., M, Wang R. Tumor-specific human CD4+ regulatory T cells and their ligands: implications for immunotherapy. Immunity. 2004; 20: 107-18.

5) Ghiringhelli F, Larmonier N, Schmitt E, Parcellier A, Cathelin D, Garrido C, Chauffert B, Solary E, Bonnotte B, and Martin F. CD4+CD25+ regulatory T cells suppress tumor immunity but are sensitive to cyclophosphamide which allows immunotherapy of established tumors to be curative. Eur J Immunol. 2004; 34: 336-44
6) Robbins P. F, Dudley M. E, Wunderlich J, El-Gamil M, Li Y. F, Zhou J, Huang J, Powell D. J, Rosenberg S. A. Cutting Edge: Persistence of Transferred lymphocyte Clonotypes Correlates with Cancer Regression in Patients Receiving Cell Transfer Therapy. J. Immunology.2004; 173: 7125-30
7) Kaech S.M, Wherry J, Ahmed R. Effector and Memory T-cell differentiation: Implications for vaccine development. Nature Rev. Immunology. 2002; 2: 251-62.
8) Schluns K.S and Lefrancois L. Cytokine control of memory T-cell development and survival. Nature Rev. Immunology. 2003; 3: 269-79.
9) Surh,C.D.and Sprent,J. Homeostasis of naïve and memory T cells. Immunity. 2008; 9: 848-62.
10) Lou Y, Wang G, Lisee G, Kim J. G, Finkelstein E.S, Feng C, Restifo P. N, Hwu P. Dendritic Cells Strongly Boost the Antitumor Activity of Adoptively Transferred T Cells In vivo. Caner Res. 2004; 64: 6783-90.
11) Tani K, Azuma M, Nakazaki Y, Oyaizu N, Hase H, Ohata J, Takahashi K, OiwaMonna M, Hanazawa K, Wakumoto Y, Kawai K, Noguchi M, Soda Y, Kunisaki R, Watari K, Takahashi S, Machida U, Satoh N, Tojo A, Maekawa T, Eriguchi M, Tomikawa S, Tahara H, Inoue Y, Yoshikawa H, Yamada Y, Iwamoto A, Hamada H, Yamashita N, Okumura K, Kakizoe T, Akaza H, Fujime M, Clift S, Ando D, Mulligan R, Asano S. Phase I study of autologous tumor vaccines transduced with the GM-CSF gene in four patients with stage IV renal cell cancer in Japan: clinical and immunological findings. Mol Ther. 2004; 10: 799-816.
12) M.E. Christine Lutsiak, Roshanak T, Semnani, Roberto De Pascalis, Syed V. S. Kashmini, Jeffrey Schiom, Helen Sabvevari. Inhibition of CD4+25+ T regulatory cell function implicated in enhanced immune response by low-dose cyclophosphamide. Blood. 2005; 105: 2862-68.

13) Ghiringhelli F, Larmonier N, Schmitt E, Parcellier A, Cathelin D, Garrido C, Chauffert B, Solary E, Bonnotte B, Martin F. CD4+CD25+ regulatory T cells suppress tumor immunity but are sensitive to cyclophosphamide which allows immunotherapy of established tumors to be curative. Eur J Immunol. 2004; 34: 336-44.

14) Konno S, Asano K, Gonokami Y, Kurokawa M, Kawazu K, Adachi M. Effect of IPD-1151T(Suplatast tosilate)on airway hyperresponsiveness in mice. Arerugi. 1995; 44: 556-61

15) C.D. Jurincic-Winklera, K.A. Metzb, J. Beuthc, K.F. Klippela. Keyhole Limpet Hemocyanin for Carcinoma in situ of the Bladder: A Long-Term Follow-Up Study Eur Urol. 2000;37:45-49

16) Yukio Iwashita, Kouichirou Tahara, Shigeru Goto, Atsushi Sasaki, Seiichiro Kai, Masataka Seike, Chao-Long Chen, Katsunori Kawano, Seigo Kitano. A phase I study of autologous dendritic cell-based immunotherapy for patients with unresectable primary liver cancer. Cancer Immunol Immunother. 2003;52:155-161

17) Lorenz Höltl, Reinhold Ramoner, Claudia Zelle-Rieser Hubert Gander, Thomas Putz, Christine Papesh, Walter Nussbaumer, Claudia Falkensammer, Georg Bartsch, Martin Thurnher. Allogeneic dendritic cell vaccination against metastatic renal cell carcinoma with or without cyclophosphamide. Cancer Immunol Immunother. 2005; 54: 663-670

18) Dudley M. E, Wunderlich J. R, Robbins P. F, Yang J. C, Hwu P, Schwartzentruber D. J, Topalian S. L, Sherry R, Restifo N. P, Hubicki A. M, Robinson M. R, Raffeld M, Duray P, Seipp C. A, Rogers-Freezer L, Morton K E, Mavroukakis S. A, White D.E, Rosenberg S. A, Cancer Regression and Autoimmunity in Patients After Clonal Repopulation with Antitumor Lymphocytes. Science. 2002; 298 (25): 850-4.

19) Cranmer LD, Trevor KT, Hersh EM. Clinical applications of dendritic cell vaccination in the treatment of cancer. Cancer Immunol immunother. 2004; 53: 275-306.

20) Chakraverty R, Peggs K, Chopra R, Milligan DW, Kottaridis PD, Verfuerth S, Geary J, Thuraisundaram D, Branson K, Chakrabarti S, Mahendra P, Craddock C,

Parker A, Hunter A, Hale G, Waldmann H, Williams CD, Yong K, Linch DC, Goldstone AH, Mackinnon S. Limmiting transplantation-related mortality following unrelated donor stem cell transplantation by using a nonmyeloablative Conditioning regimen. Blood. 2002; 99: 1071-8.

21) Dudley M E, Wunderlich J R, Sherry R M, Topalian S L, Restifo N P, Royal R E, Kammula d, White D E, Mavroukakis S A, Rogers L J, et al, Adoptive Cell Transfer Therapy Following Non-Myeloablative but Lymphodepleting Chemotherapy for the Treatment of Patients With Refractory Metastatic Melanoma. J. Clin. Oncol 2005; 23: 2346-57.
