# Supplementary material for: A phase I clinical trial of RNF43 peptide-related immune cell therapy combined with low-dose cyclophosphamide in patients with advanced solid tumors
Source: PLoS One. 2018 Jan 2;13(1):e0187878. doi: 10.1371/journal.pone.0187878 (PMC5749706; doi:10.1371/journal.pone.0187878)
Supplement: S4 File — (PDF) [file pone.0187878.s004.pdf]

Patient No.	KU−1

【PBMC phenotype】

Test item	Unit	Day1	Day28	Day49
CD3+CD4+	%	44.1	46.8	54.3
CD3+CD4+−naive	%	27.6	27.4	25.2
CD3+CD4+−effector memory	%	31.3	23.1	24.2
CD3+CD4+−central memory	%	34	45.1	46.9
CD3+CD4+−effector	%	7.2	4.4	3.7
CD3+CD8+	%	16.8	16.7	19.9
CD3+CD8+−naive	%	8.1	11.1	14.4
CD3+CD8+−effector memory	%	44.8	45.5	33.9
CD3+CD8+−central memory	%	20.9	21	36.3
CD3+CD8+−effector	%	26.2	22.5	15.4
CD3−CD56+	%	24.5	20.9	5.7

【Serum cytokine levels】

Test item	Unit	Day1	Day28	Day49		Day6
IL−1β	pg/mL	19.6	20.4	17.22		19.85
IL−2	pg/mL	0	5.67	2.64		2.54
IL−4	pg/mL	5.57	5.09	8.51		4.18
IL−6	pg/mL	13.32	160.87	34.73		87.8
IL−10	pg/mL	4.96	4.33	2.89		6.55
TNF−α	pg/mL	13.26	14.91	9.27		12.36
IFN−γ	pg/mL	0	0	0.79		0

【CD107a/b CD8+】

Test item	Unit	Day1	Day28	Day49
1)medium	%	0.8	2.2	0.4
2)RNF43antigen(−)	%	1	1.5	0.8

3)RNF43antigen(+)	%	1.1	1.5	0.3
3)−1)	%	0.3	−0.7	−0.1
3)−2)	%	0.1	0	−0.5

【Regulatory T cell】

Test item	Unit	Day1	Day6	Day28	Day49
Treg/CD4 ratio	%(/CD4)	0.4	0.2	0.6	0.3
Counts of Treg	cells(/ul)	3	5	3	2

【Th17 cell】

Test item	Unit	Day1	Day6	Day28	Day49
Th17/CD4 ratio	%(/CD4)	0.01	0.01	0.02	0.01

Patinet No.	KU-1

Intracellular cytokine staing

【ICS+phenotype：CD4+】

Test item	Unit	Day1			Day28			Day49		
		1)RNF43 antigen－	2)RNF43 antigen＋	2)-1)	1)RNF43 antigen－	2)RNF43 antigen＋	2)-1)	1)RNF43 antigen－	2)RNF43 antigen＋	2)-1)
IFN-γ-TOTAL	%	0.16	0.33	0.17	0.4	0.34	-0.06	0.25	0.11	-0.14
IFN-γ-naive	%		30.8			15,4			0	
IFN-γ-effector memory	%		30.8			38.5			100	
IFN-γ-central memory	%		30.8			46.2			0	
IFN-γ-effector	%		7.7			0			0	
IL-2-TOTAL	%	0.24	0.16	-0.08	0.05	0.37	0.32	0.05	0.14	0.09
IL-2-naive	%		42.9			15.4			0	
IL-2-effector memory	%		28.6			15.4			0	
IL-2-central memory	%		28.6			61.5			100	
IL-2-effector	%		0			7.7			0	
TNF-α-TOTAL	%	0.22	0.12	-0.1	0.15	0.14	-0.01	0.21	0.06	-0.15
TNF-α-naive	%		60			25			33.3	
TNF-α-effector memory	%		40			25			66.7	
TNF-α-central memory	%		0			50			0	
TNF-α-effector	%		0			0			0	

【ICS+phenotype：CD8+】

Test item	Unit	Day1			Day28			Day49		
		1)RNF43 antigen－	2)RNF43 antigen＋	2)-1)	1)RNF43 antigen－	2)RNF43 antigen＋	2)-1)	1)RNF43 antigen－	2)RNF43 antigen＋	2)-1)
IFN-γ-TOTAL	%	0.16	0.62	0.46	0.25	0.49	0.24	0.24	0.62	0.38
IFN-γ-naive	%		10			0			25	
IFN-γ-effector memory	%		20			14.3			25	
IFN-γ-central memory	%		50			42.9			50	

IFN−γ−effector	%		20			42.8			0	
IL−2−TOTAL	%	0.16	0.21	0.05	0.23	0	−0.23	0.51	0.42	−0.09
IL−2−naive	%		0			0			0	
IL−2−effector memory	%		75			0			50	
IL−2−central memory	%		0			0			50	
IL−2−effector	%		25			0			0	
TNF−α−TOTAL	%	0.1	0.16	0.06	0	0.09	0.09	0.15	0.32	0.17
TNF−α−naive	%		0			0			0	
TNF−α−effector memory	%		66.7			100			66.7	
TNF−α−central memory	%		33.3			0			33.3	
TNF−α−effector	%		0			0			0	

Patint No.	KU-2

【PBMC phenotype】

Test item	Unit	Day24	Day50	Day71
CD3+CD4+	%	52.3	41.8	53.4
CD3+CD4+-naive	%	14.9	28.1	28.3
CD3+CD4+-effector memory	%	45	28.7	30.4
CD3+CD4+-central memory	%	13.9	23.8	20.8
CD3+CD4+-effector	%	26.2	19.4	20.4
CD3+CD8+	%	11.2	15.8	14.8
CD3+CD8+-naive	%	6.4	6.9	7.4
CD3+CD8+-effector memory	%	59.6	58.4	57
CD3+CD8+-central memory	%	8.1	7.1	6.6
CD3+CD8+-effector	%	26	27.6	29
CD3-CD56+	%	5.7	11.9	10.8

【Serum cytokine levels】

Test item	Unit	Day1	Day28	Day49
IL-1β	pg/mL	20.47	23.71	27.85
IL-2	pg/mL	4.25	1.64	3.49
IL-4	pg/mL	3.75	6.83	7.05
IL-6	pg/mL	26.71	22.25	36.79
IL-10	pg/mL	3.25	4.76	5.47
TNF-α	pg/mL	29.63	12.35	22.98
IFN-γ	pg/mL	1.17	3.96	1.35

【CD107a/b CD8+】

Test item	Unit	Day24	Day50	Day71
1)medium	%	1.3	1.3	1.6
2)RNF43antigen(-)	%	1.5	1.5	1.4
3)RNF43antigen(+)	%	1.7	1.6	1.5
3)-1)	%	0.4	0.3	-0.1
3)-2)	%	0.2	0.1	0.1

【Regulatory T cell】

Test item	Unit	Day1	Day6	Day28
Treg/CD4 ratio	%(/CD4)	2.7	3.1	2.6
Counts of Treg	cells(/ul)	12	15	18

【Th17 cell】

Test item	Unit	Day1	Day6	Day28
Th17/CD4 ratio	%(/CD4)	0.12	0.13	0.11

	Day6
	13.11
	1.36
	3.65
	11.33
	3.28
	12.33
	1.74

Day49
2
14

Day49
0.07

Patinet No.	KU-2

Intracellular cytokine staing

【ICS+phenotype：CD4+】

Test item	Unit	Day1 1)RNF43 antigen－	Day1 2)RNF43 antigen＋	Day1 2)-1)	1)RNF43 antigen－
IFN-γ-TOTAL	%	0.2	0.22	0.02	0.29
IFN-γ-naive	%		8		
IFN-γ-effector memory	%		48		
IFN-γ-central memory	%		32		
IFN-γ-effector	%		12		
IL-2-TOTAL	%	0.2	0.29	0.09	0.18
IL-2-naive	%		17.2		
IL-2-effector memory	%		24.1		
IL-2-central memory	%		41.4		
IL-2-effector	%		17.2		
TNF-α-TOTAL	%	0.26	0.29	0.03	0.17
TNF-α-naive	%		28		
TNF-α-effector memory	%		40		
TNF-α-central memory	%		20		
TNF-α-effector	%		12		

【ICS+phenotype：CD8+】

Test item	Unit	Day1 1)RNF43 antigen－	Day1 2)RNF43 antigen＋	Day1 2)-1)	1)RNF43 antigen－
IFN-γ-TOTAL	%	0.17	0.39	0.22	0.06
IFN-γ-naive	%		0		
IFN-γ-effector memory	%		70		
IFN-γ-central memory	%		0		
IFN-γ-effector	%		30		
IL-2-TOTAL	%	0.18	0.31	0.13	0.18
IL-2-naive	%		12.5		
IL-2-effector memory	%		62.5		
IL-2-central memory	%		12.5		
IL-2-effector	%		12.5		
TNF-α-TOTAL	%	0.14	0.49	0.35	0.45
TNF-α-naive	%		0		
TNF-α-effector memory	%		63.6		
TNF-α-central memory	%		9.1		
TNF-α-effector	%		27.3		

Day28		Day49		
2)RNF43 antigen＋	2)−1)	1)RNF43 antigen－	2)RNF43 antigen＋	2)−1)
0.31	0.02	0.29	0.19	−0.1
34.4			27.8	
37.5			22.2	
25			50	
3.1			0	
0.17	−0.01	0.27	0.12	−0.15
55.6			9.1	
22.2			27.3	
11.1			63.6	
11.1			0	
0.18	0.01	0.21	0.17	−0.04
18.2			11.8	
36.4			11.8	
36.4			58.8	
9.1			17.6	

Day28		Day49		
2)RNF43 antigen＋	2)−1)	1)RNF43 antigen－	2)RNF43 antigen＋	2)−1)
0.38	0.32	0.4	0.53	0.13
6.7			13.3	
46.7			53.3	
13.3			0	
33.3			33.3	
0.47	0.29	0.27	0.14	−0.13
11.1			25	
55.6			25	
0			0	
33.3			50	
0.31	−0.14	0.37	0.22	−0.15
12.5			14.3	
50			28.6	
12.5			0	
25			57.1	

Patient No.	KU-3

【PBMC phenotype】

検査項目	Unit	Day24	Day50	Day71
CD3+CD4+	%	46	33.3	30.4
CD3+CD4+-naive	%	14.2	16.6	16.7
CD3+CD4+-effector memory	%	50.2	54.7	55.8
CD3+CD4+-central memory	%	26.1	20.8	16.5
CD3+CD4+-effector	%	9.5	7.8	11
CD3+CD8+	%	23.8	25.8	28.8
CD3+CD8+-naive	%	20.9	22.6	24.3
CD3+CD8+-effector memory	%	35.3	38.4	33.6
CD3+CD8+-central memory	%	5.5	5.3	4.6
CD3+CD8+-effector	%	38.3	33.7	37.5
CD3-CD56+	%	9.3	16.1	18.1

【Serum cytokine levels】

検査項目	Unit	Day1	Day28	Day49
IL-1β	pg/mL	18.31	23	10.89
IL-2	pg/mL	3.06	3.99	5.07
IL-4	pg/mL	7.43	6.56	3.12
IL-6	pg/mL	12.73	12.25	12.1
IL-10	pg/mL	5.07	3.08	3.38
TNF-α	pg/mL	9.95	17.04	5.24
IFN-γ	pg/mL	0.43	0.89	0

【CD107a/b CD8+】

Test item	Unit	Day1	Day28	Day49
1)medium	%	0.9	0.4	0.6
2)RNF43antigen(-)	%	1.1	0.3	1.3
3)RNF43antigen(+)	%	1.3	0.9	1.9
3)-1)	%	0.4	0.5	1.3
3)-2)	%	0.2	0.6	0.6

【Regulatory T cell】

Test item	Unit	Day1	Day6	Day28
Treg/CD4 ratio	%(/CD4)	3.8	3.2	3.7
Counts of Treg	cells(/ul)	14	14	19

【Th17 cell】

Test item	Unit	Day1	Day6	Day28
Th17/CD4 ratio	%(/CD4)	0.11	0.13	0.07

	Day6
	16.03
	1.85
	4.45
	5.76
	5
	6.58
	1.34

Day49
2.9
10

Day49
0.06

Patinet No.	KU-3

Intracellular cytokine staing

【ICS+phenotype：CD4+】

Test item	Unit	Day1			
		1)RNF43 antigen－	2)RNF43 antigen＋	2)-1)	1)RNF43 antigen－
IFN-γ-TOTAL	%	0.26	0.39	0.13	0.12
IFN-γ-naive	%		16.1		
IFN-γ-effector memory	%		45.2		
IFN-γ-central memory	%		29		
IFN-γ-effector	%		9.7		
IL-2-TOTAL	%	0.19	0.34	0.15	0.26
IL-2-naive	%		19.4		
IL-2-effector memory	%		29		
IL-2-central memory	%		41.9		
IL-2-effector	%		9.7		
TNF-α-TOTAL	%	0.16	0.17	0.01	0.13
TNF-α-naive	%		5.6		
TNF-α-effector memory	%		72.2		
TNF-α-central memory	%		16.7		
TNF-α-effector	%		5.6		

【ICS+phenotype：CD8+】

Test item	Unit	Day1			
		1)RNF43 antigen－	2)RNF43 antigen＋	2)-1)	1)RNF43 antigen－
IFN-γ-TOTAL	%	0.49	0.38	-0.11	0.15
IFN-γ-naive	%		18.8		
IFN-γ-effector memory	%		12.5		
IFN-γ-central memory	%		6.2		
IFN-γ-effector	%		62.5		
IL-2-TOTAL	%	0.33	0.68	0.35	0.2
IL-2-naive	%		19.4		
IL-2-effector memory	%		41.9		
IL-2-central memory	%		3.2		
IL-2-effector	%		35.5		
TNF-α-TOTAL	%	0.13	0.39	0.26	0.13
TNF-α-naive	%		23.8		
TNF-α-effector memory	%		23.8		
TNF-α-central memory	%		0		
TNF-α-effector	%		52.4		

Day28		Day49		
2)RNF43 antigen＋	2)−1)	1)RNF43 antigen－	2)RNF43 antigen＋	2)−1)
0.3	0.18	0.32	0.62	0.3
11.1			5.9	
22.2			47.1	
44.4			41.2	
22.2			5.9	
0.2	−0.06	0.07	0.19	0.12
11.1			16.7	
55.6			66.7	
33.3			16.7	
0			0	
0.27	0.14	0.29	0.15	−0.14
9.1			25	
45.5			25	
45.5			50	
0			0	

Day28		Day49		
2)RNF43 antigen＋	2)−1)	1)RNF43 antigen－	2)RNF43 antigen＋	2)−1)
0.5	0.35	0.34	0.57	0.23
3.8			6.2	
38.5			37.5	
3.8			6.2	
53.8			50	
0.2	0	0.14	0.1	−0.04
25			33.3	
12.5			0	
0			33.3	
62.5			33.3	
0.23	0.1	0.27	0.26	−0.01
0			42.9	
28.6			14.3	
0			0	
71.4			42.9	

Patient No.	KU-4

【PBMC phenotype】

Test item	Unit	Day24	Day50	Day71
CD3+CD4+	%	50.8	46.1	50.3
CD3+CD4+-naive	%	5.9	44	36.9
CD3+CD4+-effector memory	%	55.8	15.5	25.5
CD3+CD4+-central memory	%	14.1	36.4	30.2
CD3+CD4+-effector	%	24.1	3.7	7.5
CD3+CD8+	%	21.6	31.6	24.8
CD3+CD8+-naive	%	13.9	47.4	42.7
CD3+CD8+-effector memory	%	37.1	29.1	32.7
CD3+CD8+-central memory	%	3.3	5.9	6.1
CD3+CD8+-effector	%	45.7	17.6	18.5
CD3-CD56+	%	1.7	11.9	4.4

【Serum cytokine levels】

Test item	Unit	Day1	Day28	Day49
IL-1β	pg/mL	19.76	19.86	15.33
IL-2	pg/mL	0.92	0.16	0
IL-4	pg/mL	6.27	2.47	4.93
IL-6	pg/mL	13.78	15.2	21.52
IL-10	pg/mL	2.97	3.16	6.25
TNF-α	pg/mL	0	0	0
IFN-γ	pg/mL	0	3.26	0.04

【CD107a/b CD8+】

Test item	Unit	Day24	Day50	Day71
1)medium	%	1	0.3	0.7
2)RNF43antigen(-)	%	0.9	0.2	0.5
3)RNF43antigen(+)	%	1.2	0.2	0.8
3)-1)	%	0.2	-0.1	0.1
3)-2)	%	0.3	0	0.3

【Regulatory T cell】

Test item	Unit	Day1	Day6	Day28
Treg/CD4 ratio	%(/CD4)	1.1	0.7	2.3
Counts of Treg	cells(/ul)	4	2	7

【Th17 cell】

Test item	Unit	Day1	Day6	Day28
Th17/CD4 ratio	%(/CD4)	0.13	0.04	0.12

	Day6
	24.29
	0.98
	3.97
	15.94
	5.13
	4.69
	2.81

Day49
1.6
4

Day49
0.65

Patinet No.	KU-4

Intracellular cytokine staing

【ICS+phenotype：CD4+】

Test item	Unit	Day1 1)RNF43 antigen－	Day1 2)RNF43 antigen＋	Day1 2)-1)	1)RNF43 antigen－
IFN-γ-TOTAL	%	0.28	0.18	-0.1	0.23
IFN-γ-naive	%		22.2		
IFN-γ-effector memory	%		22.2		
IFN-γ-central memory	%		22.2		
IFN-γ-effector	%		33.3		
IL-2-TOTAL	%	0.2	0.3	0.1	0.14
IL-2-naive	%		0		
IL-2-effector memory	%		42.9		
IL-2-central memory	%		42.9		
IL-2-effector	%		14.3		
TNF-α-TOTAL	%	0.29	0.15	-0.14	0.19
TNF-α-naive	%		20		
TNF-α-effector memory	%		60		
TNF-α-central memory	%		20		
TNF-α-effector	%		0		

【ICS+phenotype：CD8+】

Test item	Unit	Day1 1)RNF43 antigen－	Day1 2)RNF43 antigen＋	Day1 2)-1)	1)RNF43 antigen－
IFN-γ-TOTAL	%	0.27	0.25	-0.02	0.24
IFN-γ-naive	%		20		
IFN-γ-effector memory	%		20		
IFN-γ-central memory	%		0		
IFN-γ-effector	%		60		
IL-2-TOTAL	%	0.11	0.47	0.36	0.21
IL-2-naive	%		0		
IL-2-effector memory	%		33.3		
IL-2-central memory	%		0		
IL-2-effector	%		66.7		
TNF-α-TOTAL	%	0.28	0.13	-0.15	0.38
TNF-α-naive	%		0		
TNF-α-effector memory	%		20		
TNF-α-central memory	%		0		
TNF-α-effector	%		80		

Day28		Day49		
2)RNF43 antigen＋	2)−1)	1)RNF43 antigen−	2)RNF43 antigen＋	2)−1)
0.29	0.06	0.15	0.17	0.02
35.6			30	
31.1			30	
24.4			30	
8.9			10	
0.09	−0.05	0.13	0.13	0
46.2			12.5	
0			25	
53.8			50	
0			12.5	
0.19	0	0.13	0.23	0.1
55			20	
5			20	
20			53.3	
20			6.7	

Day28		Day49		
2)RNF43 antigen＋	2)−1)	1)RNF43 antigen−	2)RNF43 antigen＋	2)−1)
0.86	0.62	0.29	0.55	0.26
19.1			17.6	
38.3			29.4	
1.1			5.9	
41.5			47.1	
0.24	0.03	0.05	0.1	0.05
60			66.7	
6.7			0	
6.7			0	
26.7			33.3	
0.37	−0.01	0.11	0.15	0.04
29.6			0	
51.9			40	
0			20	
18.5			40	

Patient No.	KU-5

【PBMC phenotype】

Test item	Unit	Day24	Day50	Day71
CD3+CD4+	%	25.6	28.7	34.8
CD3+CD4+-naive	%	3.7	3.4	3.2
CD3+CD4+-effector memory	%	68.5	64.4	60.1
CD3+CD4+-central memory	%	27.6	31.8	36.5
CD3+CD4+-effector	%	0.2	0.4	0.2
CD3+CD8+	%	23.2	20.4	25.1
CD3+CD8+-naive	%	9.7	10.9	11.5
CD3+CD8+-effector memory	%	55.6	52	52.8
CD3+CD8+-central memory	%	19.5	26.4	28.4
CD3+CD8+-effector	%	15.2	10.8	7.3
CD3-CD56+	%	18.6	12.1	7.3

【Serum cytokine levels】

Test item	Unit	Day1	Day28	Day49
IL-1β	pg/mL	15.18	14.97	19.06
IL-2	pg/mL	0.42	0.96	0.89
IL-4	pg/mL	0.86	3.36	3.96
IL-6	pg/mL	8.73	13.46	11.12
IL-10	pg/mL	3.14	3.74	2.86
TNF-α	pg/mL	1.79	0	0
IFN-γ	pg/mL	0	8.07	0

【CD107a/b CD8+】

Test item	Unit	Day24	Day50	Day71
1)medium	%	0.9	1.5	1.1
2)RNF43antigen(-)	%	0.5	1	1.4
3)RNF43antigen(+)	%	0.9	1.3	1.3
3)-1)	%	0	-0.2	0.2
3)-2)	%	0.4	0.3	-0.1

【Regulatory T cell】

Test item	Unit	Day1	Day6	Day28
Treg/CD4 ratio	%(/CD4)	3.5	2.2	2.8
Counts of Treg	cells(/ul)	5	5	5

【Th17 cell】

Test item	Unit	Day1	Day6	Day28
Th17/CD4 ratio	%(/CD4)	0	0.21	0.02

	Day6
	5.38
	2.7
	4.42
	10.85
	5.44
	0
	0.25

Day49
2.6
5

Day49
0

Patinet No.	KU-5

Intracellular cytokine staing

【ICS+phenotype：CD4+】

		Day1			
Test item	Unit	1)RNF43 antigen－	2)RNF43 antigen＋	2)-1)	1)RNF43 antigen－
IFN-γ-TOTAL	%	0.21	0.06	-0.15	0.65
IFN-γ-naive	%		33.3		
IFN-γ-effector memory	%		33.3		
IFN-γ-central memory	%		33.3		
IFN-γ-effector	%		0		
IL-2-TOTAL	%	0.2	0.04	-0.16	0.22
IL-2-naive	%		0		
IL-2-effector memory	%		50		
IL-2-central memory	%		0		
IL-2-effector	%		50		
TNF-α-TOTAL	%	0.15	0.2	0.05	0.15
TNF-α-naive	%		0		
TNF-α-effector memory	%		40		
TNF-α-central memory	%		60		
TNF-α-effector	%		0		

【ICS+phenotype：CD8+】

		Day1			
Test item	Unit	1)RNF43 antigen－	2)RNF43 antigen＋	2)-1)	1)RNF43 antigen－
IFN-γ-TOTAL	%	0.36	0.22	-0.14	0.71
IFN-γ-naive	%		20		
IFN-γ-effector memory	%		50		
IFN-γ-central memory	%		20		
IFN-γ-effector	%		10		
IL-2-TOTAL	%	0.25	0.22	-0.03	0.66
IL-2-naive	%		33.3		
IL-2-effector memory	%		55.6		
IL-2-central memory	%		11.1		
IL-2-effector	%		0		
TNF-α-TOTAL	%	0.21	0.37	0.16	0.48
TNF-α-naive	%		23.5		
TNF-α-effector memory	%		29.4		
TNF-α-central memory	%		23.5		
TNF-α-effector	%		23.5		

Day28		Day49		
2)RNF43 antigen＋	2)−1)	1)RNF43 antigen−	2)RNF43 antigen＋	2)−1)
0.83	0.18	0.37	0.59	0.22
8.3			2.2	
58.3			60	
33.3			37.8	
0			0	
0.58	0.36	0.28	0.67	0.39
7.1			4.3	
35.7			31.9	
57.1			61.7	
0			2.1	
0.2	0.05	0.27	0.72	0.45
4.8			6.1	
61.9			42.4	
28.6			51.5	
4.8			0	

Day28		Day49		
2)RNF43 antigen＋	2)−1)	1)RNF43 antigen−	2)RNF43 antigen＋	2)−1)
0.83	0.12	0.39	0.59	0.2
24.2			23.3	
21.2			26.7	
45.5			46.7	
9.1			3.3	
0.58	−0.08	0.59	0.66	0.07
12.5			31.2	
45.8			18.8	
33.3			43.8	
8.3			6.2	
0.44	−0.04	0.47	0.72	0.25
11.1			25.7	
50			20	
33.3			51.4	
5.6			2.9	

Patient No.	KU-6

【PBMC phenotype】

Test item	Unit	Day24	Day50	Day71
CD3+CD4+	%	53.7	56.7	60.2
CD3+CD4+-naive	%	52.2	45	48.5
CD3+CD4+-effector memory	%	23.4	27.2	24.6
CD3+CD4+-central memory	%	20.8	25.3	24.4
CD3+CD4+-effector	%	3.6	2.6	2.5
CD3+CD8+	%	13.7	15.6	14.3
CD3+CD8+-naive	%	59.3	52.2	54.8
CD3+CD8+-effector memory	%	14.6	19.9	16.4
CD3+CD8+-central memory	%	9	10.3	11.5
CD3+CD8+-effector	%	17.1	17.6	17.3
CD3-CD56+	%	8	10	7.9

【Serum cytokine levels】

Test item	Unit	Day1	Day28	Day49
IL-1β	pg/mL	23.4	20.6	12.6
IL-2	pg/mL	1.05	1.53	2.28
IL-4	pg/mL	5.98	4.67	5.46
IL-6	pg/mL	12.39	6.38	35.8
IL-10	pg/mL	2.01	2.97	3.45
TNF-α	pg/mL	0	0	0
IFN-γ	pg/mL	5.4	0	1.26

【CD107a/b CD8+】

Test item	Unit	Day24	Day50	Day71
1)medium	%	9.7	6.8	12.1
2)RNF43antigen(-)	%	9.1	7.6	11.1
3)RNF43antigen(+)	%	7.4	7.8	10.4
3)-1)	%	-2.3	1	-1.7
3)-2)	%	-1.7	0.2	-0.7

【Regulatory T cell】

Test item	Unit	Day1	Day6	Day28
Treg/CD4 ratio	%(/CD4)	2.5	2.3	2.7
Counts of Treg	cells(/ul)	22	14	23

【Th17 cell】

Test item	Unit	Day1	Day6	Day28
Th17/CD4 ratio	%(/CD4)	0.01	0	0.01

	Day6
	16.62
	0.82
	5.81
	9.24
	3.2
	0
	0

Day49
2.1
15

Day49
0.02

Patinet No.	KU-6

Intracellular cytokine staing

【ICS+phenotype：CD4+】

Test item	Unit	Day1 1)RNF43 antigen－	Day1 2)RNF43 antigen＋	Day1 2)-1)	1)RNF43 antigen－
IFN-γ-TOTAL	%	0.15	0.19	0.04	0.2
IFN-γ-naive	%		73.9		
IFN-γ-effector memory	%		13		
IFN-γ-central memory	%		13		
IFN-γ-effector	%		0		
IL-2-TOTAL	%	0.04	0.15	0.11	0.18
IL-2-naive	%		62.5		
IL-2-effector memory	%		12.5		
IL-2-central memory	%		25		
IL-2-effector	%		0		
TNF-α-TOTAL	%	0.07	0.06	-0.01	0.18
TNF-α-naive	%		100		
TNF-α-effector memory	%		0		
TNF-α-central memory	%		0		
TNF-α-effector	%		0		

【ICS+phenotype：CD8+】

Test item	Unit	Day1 1)RNF43 antigen－	Day1 2)RNF43 antigen＋	Day1 2)-1)	1)RNF43 antigen－
IFN-γ-TOTAL	%	0.14	0.07	-0.07	0.32
IFN-γ-naive	%		50		
IFN-γ-effector memory	%		0		
IFN-γ-central memory	%		0		
IFN-γ-effector	%		50		
IL-2-TOTAL	%	0.25	0.11	-0.14	0.34
IL-2-naive	%		66.7		
IL-2-effector memory	%		0		
IL-2-central memory	%		-		
IL-2-effector	%		33.3		
TNF-α-TOTAL	%	0.14	0.07	-0.07	0.29
TNF-α-naive	%		50		
TNF-α-effector memory	%		50		
TNF-α-central memory	%		0		
TNF-α-effector	%		0		

Day28		Day49		
2)RNF43 antigen＋	2)−1)	1)RNF43 antigen−	2)RNF43 antigen＋	2)−1)
0.21	0.01	0.43	0.23	−0.2
54.2			55.6	
16.7			14.8	
29.2			29.6	
0			0	
0.25	0.07	0.17	0.22	0.05
77.8			69.2	
0			3.8	
22.2			23.1	
0			3.8	
0.32	0.14	0.2	0.25	0.05
85.7			86.7	
2.9			0	
8.6			13.3	
2.9			0	

Day28		Day49		
2)RNF43 antigen＋	2)−1)	1)RNF43 antigen−	2)RNF43 antigen＋	2)−1)
0.45	0.13	0.49	0.36	−0.13
85.7			70	
0			0	
7.1			0	
7.1			30	
0.15	−0.19	0.24	0.21	−0.03
100			83.3	
0			0	
0			16.7	
0			0	
0.28	−0.01	0.31	0.27	−0.04
88.9			75	
0			12.5	
11.1			0	
0			12.5	

Patient No.	KU-7

【PBMC phenotype】

Test item	Unit	Day24	Day50	Day71
CD3+CD4+	%	51.6	55.6	51.2
CD3+CD4+-naive	%	33.9	31.9	28.2
CD3+CD4+-effector memory	%	30.6	32.7	34.2
CD3+CD4+-central memory	%	24.2	25.8	22.3
CD3+CD4+-effector	%	11.3	9.6	15.3
CD3+CD8+	%	14.8	13.8	15.8
CD3+CD8+-naive	%	26.3	29.6	22
CD3+CD8+-effector memory	%	34.2	31.4	36.2
CD3+CD8+-central memory	%	8.7	11	9.7
CD3+CD8+-effector	%	30.9	28.1	32
CD3-CD56+	%	6.4	5.1	7.1

【Serum cytokine levels】

Test item	Unit	Day1	Day28	Day49
IL-1β	pg/mL	13.66	14.9	19.71
IL-2	pg/mL	2.21	0.46	1.15
IL-4	pg/mL	4.52	3.29	4.63
IL-6	pg/mL	17.53	19.8	22.88
IL-10	pg/mL	3.1	4.88	4.01
TNF-α	pg/mL	0	0	0
IFN-γ	pg/mL	3.3	0	2.58

【CD107a/b CD8+】

Test item	Unit	Day24	Day50	Day71
1)medium	%	2.1	2.4	2.8
2)RNF43antigen(-)	%	1.4	2.4	2.4
3)RNF43antigen(+)	%	1.5	2.4	2.5
3)-1)	%	-0.6	0	-0.3
3)-2)	%	0.1	0	0.1

【Regulatory T cell】

Test item	Unit	Day1	Day6	Day28
Treg/CD4 ratio	%(/CD4)	2.2	1.4	1.9
Counts of Treg	cells(/ul)	19	9	17

【Th17 cell】

Test item	Unit	Day1	Day6	Day28
Th17/CD4 ratio	%(/CD4)	0	0	0

	Day6
	12.37
	0.07
	1.87
	15.12
	3.07
	0.9
	0

Day49
1.5
12

Day49
0

Patinet No.	KU-7

Intracellular cytokine staing

【ICS+phenotype：CD4+】

Test item	Unit	Day1 1)RNF43 antigen－	Day1 2)RNF43 antigen＋	Day1 2)-1)	1)RNF43 antigen－
IFN-γ-TOTAL	%	0.23	0.15	-0.08	0.15
IFN-γ-naive	%		50		
IFN-γ-effector memory	%		8.3		
IFN-γ-central memory	%		20.8		
IFN-γ-effector	%		20.8		
IL-2-TOTAL	%	0.04	0.11	0.07	0.13
IL-2-naive	%		72.2		
IL-2-effector memory	%		5.6		
IL-2-central memory	%		16.7		
IL-2-effector	%		5.6		
TNF-α-TOTAL	%	0.06	0.1	0.04	0.09
TNF-α-naive	%		12.5		
TNF-α-effector memory	%		43.8		
TNF-α-central memory	%		31.2		
TNF-α-effector	%		12.5		

【ICS+phenotype：CD8+】

Test item	Unit	Day1 1)RNF43 antigen－	Day1 2)RNF43 antigen＋	Day1 2)-1)	1)RNF43 antigen－
IFN-γ-TOTAL	%	0.15	0.19	0.04	0.17
IFN-γ-naive	%		55.6		
IFN-γ-effector memory	%		11.1		
IFN-γ-central memory	%		11.1		
IFN-γ-effector	%		22.2		
IL-2-TOTAL	%	0.02	0.09	0.07	0.12
IL-2-naive	%		50		
IL-2-effector memory	%		25		
IL-2-central memory	%		0		
IL-2-effector	%		25		
TNF-α-TOTAL	%	0.17	0.07	-0.1	0.12
TNF-α-naive	%		100		
TNF-α-effector memory	%		0		
TNF-α-central memory	%		0		
TNF-α-effector	%		0		

Day28		Day49		
2)RNF43 antigen＋	2)−1)	1)RNF43 antigen－	2)RNF43 antigen＋	2)−1)
0.09	−0.06	0.3	0.2	−0.1
56.2			53.1	
12.5			12.5	
18.8			18.8	
12.5			15.6	
0.12	−0.01	0.21	0.2	−0.01
61.9			60.6	
0			9.1	
23.8			27.3	
14.3			3	
0.11	0.02	0.18	0.19	0.01
63.2			46.7	
15.8			10	
21.1			6.7	
0			36.7	

Day28		Day49		
2)RNF43 antigen＋	2)−1)	1)RNF43 antigen－	2)RNF43 antigen＋	2)−1)
0.14	−0.03	0.09	0.21	0.12
66.7			40	
16.7			10	
0			10	
16.7			40	
0.21	0.09	0.27	0.23	−0.04
66.7			58.3	
0			8.3	
0			8.3	
33.3			25	
0.14	0.02	0.25	0.18	−0.07
66.7			44.4	
0			33.3	
0			11.1	
33.3			11.1	

Patient No.	KU-8

【PBMC phenotype】

Test item	Unit	Day24	Day50	Day71
CD3+CD4+	%	38.1	40.2	36.9
CD3+CD4+-naive	%	15.3	16.9	13.2
CD3+CD4+-effector memory	%	31.8	26.1	30.5
CD3+CD4+-central memory	%	50	54.7	53.6
CD3+CD4+-effector	%	3	2.3	2.7
CD3+CD8+	%	39	38	41
CD3+CD8+-naive	%	7.3	8.6	6.6
CD3+CD8+-effector memory	%	29.1	24.5	26.7
CD3+CD8+-central memory	%	61.4	65.1	65
CD3+CD8+-effector	%	2.4	1.8	1.7
CD3-CD56+	%	3.2	3.3	3.6

【Serum cytokine levels】

Test item	Unit	Day1	Day28	Day49
IL-1β	pg/mL	4.31	16.39	12.45
IL-2	pg/mL	1.57	2.54	1.17
IL-4	pg/mL	4.08	4.23	3.76
IL-6	pg/mL	12.04	10.9	16.33
IL-10	pg/mL	1.75	5.18	3.27
TNF-α	pg/mL	0	0	0
IFN-γ	pg/mL	17.54	17.33	10.5

【CD107a/b CD8+】

Test item	Unit	Day24	Day50	Day71
1)medium	%	0.2	0.2	0.2
2)RNF43antigen(-)	%	0.2	0.2	0.2
3)RNF43antigen(+)	%	0.3	0.3	0.4
3)-1)	%	0.1	0.1	0.2
3)-2)	%	0.1	0.1	0.2

【Regulatory T cell】

Test item	Unit	Day1	Day6	Day28
Treg/CD4 ratio	%(/CD4)	2.8	2.6	1.3
Counts of Treg	cells(/ul)	15	11	7

【Th17 cell】

Test item	Unit	Day1	Day6	Day28
Th17/CD4 ratio	%(/CD4)	0.1	0.31	0.7

	Day6
	13.01
	0.72
	1.83
	9.19
	2.37
	0
	20.11

Day49
1.4
6

Day49
0.3

Patinet No.	KU-8

Intracellular cytokine staing

【ICS+phenotype：CD4+】

Test item	Unit	Day1			
		1)RNF43 antigen－	2)RNF43 antigen＋	2)-1)	1)RNF43 antigen－
IFN-γ-TOTAL	%	0.6	1.1	0.5	0.3
IFN-γ-naive	%		32.8		
IFN-γ-effector memory	%		10.2		
IFN-γ-central memory	%		53.9		
IFN-γ-effector	%		3.1		
IL-2-TOTAL	%	0.2	0.2	0	1.71
IL-2-naive	%		28.7		
IL-2-effector memory	%		8.5		
IL-2-central memory	%		59.6		
IL-2-effector	%		3.1		
TNF-α-TOTAL	%	0.19	0.14	-0.05	0.06
TNF-α-naive	%		35.3		
TNF-α-effector memory	%		5.9		
TNF-α-central memory	%		58.8		
TNF-α-effector	%		0		

【ICS+phenotype：CD8+】

Test item	Unit	Day1			
		1)RNF43 antigen－	2)RNF43 antigen＋	2)-1)	1)RNF43 antigen－
IFN-γ-TOTAL	%	0.53	0.83	0.3	0.28
IFN-γ-naive	%		29.2		
IFN-γ-effector memory	%		21.9		
IFN-γ-central memory	%		45.8		
IFN-γ-effector	%		3.1		
IL-2-TOTAL	%	1.2	1.43	0.23	1.12
IL-2-naive	%		23.4		
IL-2-effector memory	%		15.8		
IL-2-central memory	%		53.8		
IL-2-effector	%		7		
TNF-α-TOTAL	%	0.19	0.17	-0.02	0.2
TNF-α-naive	%		20		
TNF-α-effector memory	%		35		
TNF-α-central memory	%		45		
TNF-α-effector	%		0		

Day28		Day49		
2)RNF43 antigen＋	2)−1)	1)RNF43 antigen−	2)RNF43 antigen＋	2)−1)
0.41	0.11	0.16	0.22	0.06
51			28	
6.1			20	
36.7			52	
6.1			0	
0.61	−1.1	0.93	0.6	−0.33
70.3			40.3	
2.7			9	
21.6			49.3	
5.4			1.5	
0.56	0.5	0.21	0.19	−0.02
40			57.1	
7.1			9.5	
48.6			33.3	
4.3			0	

Day28		Day49		
2)RNF43 antigen＋	2)−1)	1)RNF43 antigen−	2)RNF43 antigen＋	2)−1)
0.5	0.22	0.08	0.35	0.27
31.6			26.2	
8.8			2.4	
56.1			71.4	
3.5			0	
0.41	−0.71	0.67	0.58	−0.09
61.7			42.3	
2.1			9.9	
29.8			46.5	
6.4			1.4	
0.49	0.29	0.15	0.25	0.1
28.1			12.9	
7			22.6	
63.2			51.6	
1.8			12.9	

Patient No.	KU-9

【PBMC phenotype】

Test item	Unit	Day24	Day50	Day71
CD3+CD4+	%	40	41.3	57.6
CD3+CD4+-naive	%	35.1	34.2	55.6
CD3+CD4+-effector memory	%	16.4	15.3	7.3
CD3+CD4+-central memory	%	46.1	49.3	33.5
CD3+CD4+-effector	%	2.4	1.2	3.7
CD3+CD8+	%	32.6	30.9	22.3
CD3+CD8+-naive	%	40.7	43.9	68.5
CD3+CD8+-effector memory	%	25.4	25.6	10.2
CD3+CD8+-central memory	%	22.4	19.1	11.4
CD3+CD8+-effector	%	11.6	11.3	9.4
CD3-CD56+	%	1.7	3.7	2.4

【Serum cytokine levels】

Test item	Unit	Day1	Day28	Day49
IL-1β	pg/mL	25.26	16.36	16.86
IL-2	pg/mL	8.14	0.94	0.7
IL-4	pg/mL	5.99	3.48	4.14
IL-6	pg/mL	27.57	69.42	61.48
IL-10	pg/mL	9.06	7.69	8.57
TNF-α	pg/mL	0	0	1.14
IFN-γ	pg/mL	1.34	2.55	3.14

【CD107a/b CD8+】

Test item	Unit	Day24	Day50	Day71
1)medium	%	0.2	0.3	0.9
2)RNF43antigen(-)	%	0.2	0.3	1.1
3)RNF43antigen(+)	%	0.2	0.4	0.9
3)-1)	%	0	0.1	0
3)-2)	%	0	0.1	-0.2

【Regulatory T cell】

Test item	Unit	Day1	Day6	Day28
Treg/CD4 ratio	%(/CD4)	2.2	1.6	3.2
Counts of Treg	cells(/ul)	11	6	11

【Th17 cell】

Test item	Unit	Day1	Day6	Day28
Th17/CD4 ratio	%(/CD4)	0.02	0	0.06

	Day6
	16.92
	1.63
	3.72
	27.66
	2.66
	0
	1.98

Day49
1.9
8

Day49
0.16

Patinet No.	KU-9

Intracellular cytokine staing

【ICS+phenotype：CD4+】

Test item	Unit	Day1 1)RNF43 antigen－	Day1 2)RNF43 antigen＋	Day1 2)-1)	1)RNF43 antigen－
IFN-γ-TOTAL	%	3.1	0.29	-2.81	2.65
IFN-γ-naive	%		50		
IFN-γ-effector memory	%		10.4		
IFN-γ-central memory	%		38.5		
IFN-γ-effector	%		1		
IL-2-TOTAL	%	3.56	0.95	-2.61	0.46
IL-2-naive	%		76.4		
IL-2-effector memory	%		2.8		
IL-2-central memory	%		17		
IL-2-effector	%		3.8		
TNF-α-TOTAL	%	1.35	0.68	-0.67	1.46
TNF-α-naive	%		41.6		
TNF-α-effector memory	%		10.4		
TNF-α-central memory	%		46.8		
TNF-α-effector	%		1.3		

【ICS+phenotype：CD8+】

Test item	Unit	Day1 1)RNF43 antigen－	Day1 2)RNF43 antigen＋	Day1 2)-1)	1)RNF43 antigen－
IFN-γ-TOTAL	%	4	0.36	-3.64	2.87
IFN-γ-naive	%		66		
IFN-γ-effector memory	%		10.6		
IFN-γ-central memory	%		12.8		
IFN-γ-effector	%		10.6		
IL-2-TOTAL	%	4.09	1.09	-3	0.75
IL-2-naive	%		74.2		
IL-2-effector memory	%		3.1		
IL-2-central memory	%		8.2		
IL-2-effector	%		14.4		
TNF-α-TOTAL	%	1.36	0.71	-0.65	1.93
TNF-α-naive	%		71		
TNF-α-effector memory	%		7.2		
TNF-α-central memory	%		8.7		
TNF-α-effector	%		13		

Day28		Day49		
2)RNF43 antigen ＋	2)−1)	1)RNF43 antigen−	2)RNF43 antigen＋	2)−1)
0.76	−1.89	3.66	0.29	−3.37
68.1			79.3	
5.3			2.4	
24.8			14.6	
1.8			3.7	
0.36	−0.1	1.81	0.82	−0.99
86			85.6	
0			1.4	
14			8.6	
0			4.3	
1.27	−0.19	1.4	1.87	0.47
71.7			83.7	
2.5			0.9	
24.5			10.3	
1.3			5	

Day28		Day49		
2)RNF43 antigen＋	2)−1)	1)RNF43 antigen−	2)RNF43 antigen＋	2)−1)
1.32	−1.55	4.08	0.19	−3.89
65.6			100	
9.6			0	
16			0	
8.8			0	
0.47	−0.28	1.8	0.98	−0.82
86.7			91	
6.7			1.5	
2.2			1.5	
4.4			6	
2.14	0.21	1.6	2.42	0.82
72.7			92.6	
5.7			0.6	
16.5			3.1	
5.1			3.7	

Patient No.	KU-10

【PBMC phenotype】

Test item	Unit	Day1	Day28	Day49
CD3+CD4+	%	46.8	42.2	38.9
CD3+CD4+-naive	%	41.4	47.3	22
CD3+CD4+-effector memory	%	13.8	13.8	32
CD3+CD4+-central memory	%	42.7	35.3	35.4
CD3+CD4+-effector	%	2.1	3.6	10.6
CD3+CD8+	%	25.4	28.1	21.7
CD3+CD8+-naive	%	31.2	24.5	19.1
CD3+CD8+-effector memory	%	35.8	43.1	48.7
CD3+CD8+-central memory	%	12.3	5.3	5.9
CD3+CD8+-effector	%	20.7	27.1	26.3
CD3-CD56+	%	1.4	1.6	0.8

【Serum cytokine levels】

Test item	Unit	Day1	Day28	Day49
IL-1β	pg/mL	18.99	17.27	8.4
IL-2	pg/mL	1.42	0.42	1.07
IL-4	pg/mL	8.22	4.73	3.4
IL-6	pg/mL	11.51	11.77	11.58
IL-10	pg/mL	4.39	2.96	2.05
TNF-α	pg/mL	0	0	0
IFN-γ	pg/mL	15.79	14.17	7.54

【CD107a/b CD8+】

Test item	Unit	Day1	Day28	Day49
1)medium	%	0.6	0.3	0.4
2)RNF43antigen(-)	%	0.6	0.4	0.4
3)RNF43antigen(+)	%	0.6	0.7	0.6
3)-1)	%	0	0.4	0.2
3)-2)	%	0	0.3	0.2

【Regulatory T cell】

Test item	Unit	Day1	Day6	Day28
Treg/CD4 ratio	%(/CD4)	3.2	2.7	2.9
Counts of Treg	cells(/ul)	17	13	17

【Th17 cell】

Test item	Unit	Day1	Day6	Day28
Th17/CD4 ratio	%(/CD4)	0.04	0.08	0.1

	Day6
	15.34
	1.82
	7.2
	13.52
	3.57
	0.5
	17.92

Day49
1.5
7

Day49
0.25

Patinet No.	KU-10

Intracellular cytokine staing

【ICS+phenotype：CD4+】

Test item	Unit	Day1 1)RNF43 antigen－	Day1 2)RNF43 antigen＋	Day1 2)-1)	1)RNF43 antigen－
IFN-γ-TOTAL	%	0.29	0.25	-0.04	0.29
IFN-γ-naive	%		45.8		
IFN-γ-effector memory	%		12.5		
IFN-γ-central memory	%		41.7		
IFN-γ-effector	%		0		
IL-2-TOTAL	%	0.34	0.47	0.13	0.3
IL-2-naive	%		52.3		
IL-2-effector memory	%		2.3		
IL-2-central memory	%		40.9		
IL-2-effector	%		4.5		
TNF-α-TOTAL	%	0.44	0.63	0.19	0.3
TNF-α-naive	%		43.3		
TNF-α-effector memory	%		8.3		
TNF-α-central memory	%		48.3		
TNF-α-effector	%		0		

【ICS+phenotype：CD8+】

Test item	Unit	Day1 1)RNF43 antigen－	Day1 2)RNF43 antigen＋	Day1 2)-1)	1)RNF43 antigen－
IFN-γ-TOTAL	%	0.57	0.31	-0.26	0.5
IFN-γ-naive	%		37.5		
IFN-γ-effector memory	%		20.8		
IFN-γ-central memory	%		29.2		
IFN-γ-effector	%		12.5		
IL-2-TOTAL	%	0.25	0.72	0.47	0.52
IL-2-naive	%		40.5		
IL-2-effector memory	%		27		
IL-2-central memory	%		13.5		
IL-2-effector	%		18.9		
TNF-α-TOTAL	%	0.15	0.54	0.39	0.35
TNF-α-naive	%		32.1		
TNF-α-effector memory	%		14.3		
TNF-α-central memory	%		28.6		
TNF-α-effector	%		25		

Day28		Day49		
2)RNF43 antigen＋	2)−1)	1)RNF43 antigen−	2)RNF43 antigen＋	2)−1)
0.45	0.16	0.42	0.61	0.19
44.7			23.1	
15.8			17.3	
39.5			55.8	
0			3.8	
0.42	0.12	0.19	0.46	0.27
50			18.4	
8.3			21.1	
38.9			52.6	
2.8			7.9	
0.48	0.18	0.28	0.62	0.34
48.1			17.6	
7.4			17.6	
40.7			64.7	
3.7			0	

Day28		Day49		
2)RNF43 antigen＋	2)−1)	1)RNF43 antigen−	2)RNF43 antigen＋	2)−1)
0.63	0.13	0.47	0.57	0.1
35.1			29.6	
43.2			33.3	
2.7			11.1	
18.9			25.9	
0.6	0.08	0.47	0.37	−0.1
48.6			58.8	
20			35.3	
8.6			0	
22.9			5.9	
0.43	0.08	0.26	0.69	0.43
48			28.1	
36			28.1	
8			21.9	
8			21.9	
