# Supplementary figures and images for: A phase I clinical trial of RNF43 peptide-related immune cell therapy combined with low-dose cyclophosphamide in patients with advanced solid tumors
Source: PLoS One. 2018 Jan 2;13(1):e0187878. doi: 10.1371/journal.pone.0187878 (PMC5749706; doi:10.1371/journal.pone.0187878)

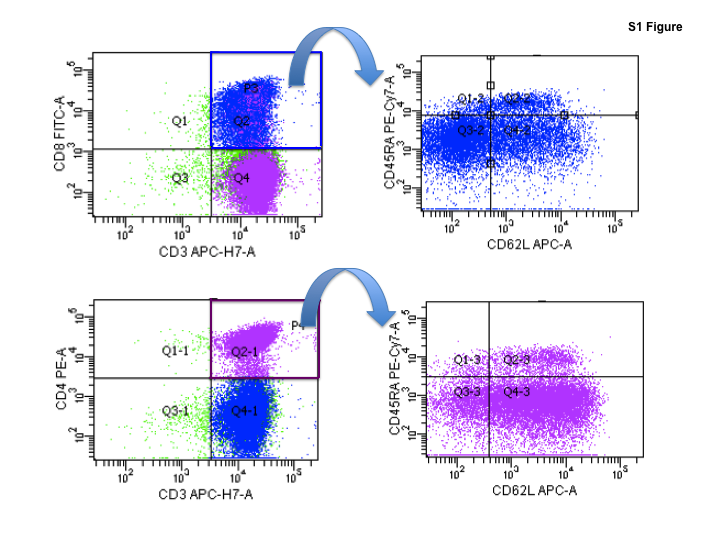

Supplement: S1 Fig — DAKs were analyzed after gating lymphocytes using forward scatter and side scatter. Representative flow plot of CD3+CD4+ and CD8+ T cells are separated into naïve (CD45RA+CD62L+), effector memory (CD45RA-CD62L-), central memory (CD45RA-CD62L+) and terminal effector (CD45RA+CD62L-). (TIF) [file pone.0187878.s006.tif]

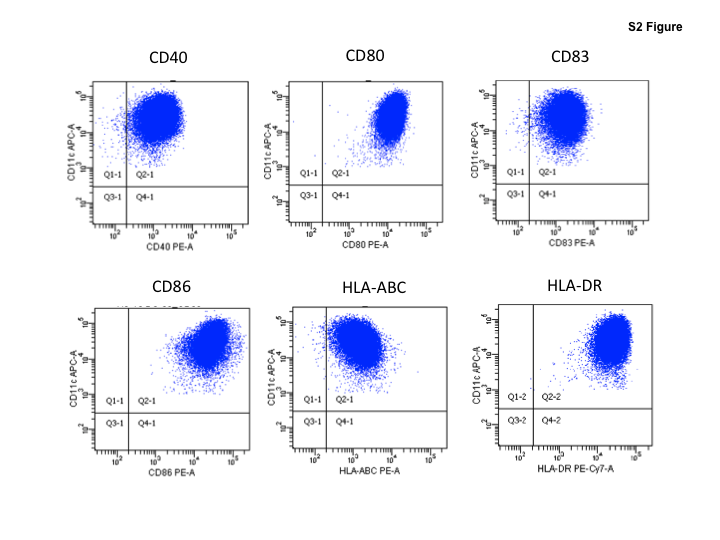

Supplement: S2 Fig — The cell surface phenotypes of each gated DCs population were analyzed after gating monocytes using forward scatter and side scatter by flow cytometry. Matured CD11c+DCs were indicated in each panel expressing high levels of CD40, CD80, CD83, CD86, HLA-ABC and HLA-DR. (TIF) [file pone.0187878.s007.tif]

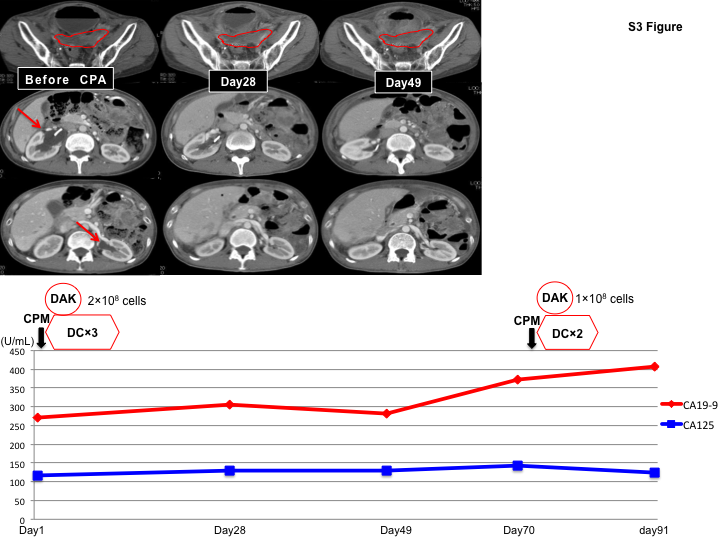

Supplement: S3 Fig — Although the size of the tumor mass in the pelvis and the levels of the two tumor markers decreased slightly, bilateral ureterohydronephrosis improved after this trial. (TIF) [file pone.0187878.s008.tif]

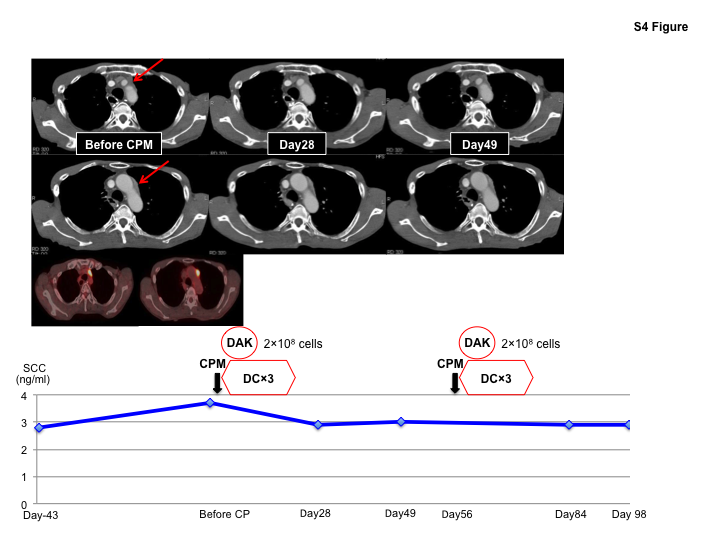

Supplement: S4 Fig — The red arrows indicate the metastasized tumors in the mediastinum. They were enhanced by positron emission tomography-computed tomography (the bottom of the image before CPA). The target lesions did not change during the observation period. The serum level of tumor marker of squamous cell carcinoma (SCC) decreased and was maintained at a reduced level after both treatments. (TIF) [file pone.0187878.s009.tif]

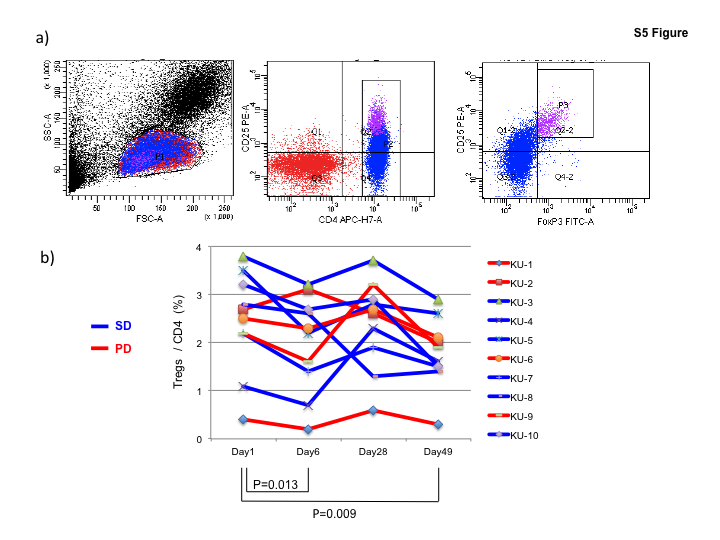

Supplement: S5 Fig — a) PBMCs were stained for CD4, CD25, Foxp3 and analyzed by means of flow cytometry. Tregs are illustrated by costaining of CD4+, CD25 high and Foxp3+. b) The frequency of peripheral blood Tregs in all patients decreased significantly between the pre-CPA time point and Day 6 (p = 0.013) or Day 49 (P = 0.009). (TIF) [file pone.0187878.s010.tif]
